# Identification of differentially methylated regions in rare diseases from a single-patient perspective

**DOI:** 10.1186/s13148-022-01403-7

**Published:** 2022-12-16

**Authors:** Robin Grolaux, Alexis Hardy, Catharina Olsen, Sonia Van Dooren, Guillaume Smits, Matthieu Defrance

**Affiliations:** 1 Interuniversity Institute of Bioinformatics in Brussels, Université Libre de Bruxelles, Brussels, Belgium; 2 Center of Human Genetics, Hôpital Erasme, Université Libre de Bruxelles, Brussels, Belgium; 3 Clinical Sciences, Research Group Reproduction and Genetics, Brussels Interuniversity Genomics High Throughput Core (BRIGHTcore), Vrije Universiteit Brussel (VUB), Universitair Ziekenhuis Brussel (UZ Brussel), Brussels, Belgium; 4 Clinical Sciences, Research Group Reproduction and Genetics, Centre for Medical Genetics, Vrije Universiteit Brussel (VUB), Universitair Ziekenhuis Brussel (UZ Brussel), Brussels, Belgium; 5 Interuniversity Institute of Bioinformatics in Brussels, Vrije Universiteit Brussel (VUB), Brussels, Belgium

**Keywords:** DNA methylation, Differentially methylated regions, Rare diseases, Imprinting, Multilocus imprinting disturbance, Statistical method, Epivariation, Beckwith–Wiedemann syndrome, Neurodevelopmental disorders, Congenital disease, Single patient, Optimization

## Abstract

**Background:**

DNA methylation (5-mC) is being widely recognized as an alternative in the detection of sequence variants in the diagnosis of some rare neurodevelopmental and imprinting disorders. Identification of alterations in DNA methylation plays an important role in the diagnosis and understanding of the etiology of those disorders. Canonical pipelines for the detection of differentially methylated regions (DMRs) usually rely on inter-group (e.g., case versus control) comparisons. However, these tools might perform suboptimally in the context of rare diseases and multilocus imprinting disturbances due to small cohort sizes and inter-patient heterogeneity. Therefore, there is a need to provide a simple but statistically robust pipeline for scientists and clinicians to perform differential methylation analyses at the single patient level as well as to evaluate how parameter fine-tuning may affect differentially methylated region detection.

**Result:**

We implemented an improved statistical method to detect differentially methylated regions in correlated datasets based on the Z-score and empirical Brown aggregation methods from a single-patient perspective. To accurately assess the predictive power of our method, we generated semi-simulated data using a public control population of 521 samples and investigated how the size of the control population, methylation difference, and region size affect DMR detection. In addition, we validated the detection of methylation events in patients suffering from rare multi-locus imprinting disturbance and evaluated how this method could complement existing tools in the context of clinical diagnosis.

**Conclusion:**

In this study, we present a robust statistical method to perform differential methylation analysis at the single patient level and describe its optimal parameters to increase DMRs identification performance. Finally, we show its diagnostic utility when applied to rare disorders.

**Supplementary Information:**

The online version contains supplementary material available at 10.1186/s13148-022-01403-7.

## Background

DNA methylation (DNAm) of cytosines (5-mC) plays an important role in cell biology, most notably in tissue-specific regulation of gene expression. Other roles include X-chromosome inactivation, regulation of splice junctions, and genomic imprinting [1, 2] Differential methylation of cytosines, or epivariation, has been linked to a wide array of diseases such as cancer, aging, metabolic, cardiovascular, neurodevelopmental, and autoimmune disorders [3–9], as well as other variables like the body mass index (BMI), smoking status or ethnicity [10–13]. Differential methylation can occur either at single cytosines (DMCs) or affect several loci within a region, resulting in differentially methylated regions (DMRs). Depending on their origin, primary and secondary epivariations can be differentiated. Primary epivariations arise from stochastic errors in the establishment or maintenance of a methylation state by the DNA methyl transferase proteins family. Secondary epivariations, by contrast, derive from genetic alterations such as copy number variations (CNVs) or single nucleotide variations (SNVs) at the differentially methylated locus or inactivating variants in trans-acting factors with a key role in the establishment or maintenance of methylation state of that locus [14]. Both primary and secondary epivariations are found in patients suffering from rare diseases, a worldwide public health issue estimated to affect between 260 and 445 million people [15]. On the one hand, primary epivariations are the main molecular event causing some imprinting disorders [16], rare cases of cancer [17, 18], and neurodevelopmental diseases [19]. On the other hand, secondary epivariations are a known alternative mechanism in rare diseases and the detection of these sequence variants has gained popularity in the diagnostic process. That is the case in the group of neurodevelopmental disorders known as the Mendelian disorders of the epigenetic machinery (MDEMs), for which detection of episignatures (i.e., group of DMCs acting as a blueprint for the disease) has been shown to enable patient diagnosis [20–28], or in imprinting disorders [29–34], where DMRs are localized at imprinting control centers. Episignatures and DMRs at imprinting loci are usually linked to a single disease. However, it has been shown that MDEMs’ episignatures sometimes share overlapping DMCs [35] and there have been increasing reports of patients showing multi-locus imprinting disturbances (MLIDs). MLIDs represent rare cases of imprinting disorders characterized at the molecular level by several defects at imprinting regions [36]. Patients suffering from MLIDs often share overlapping phenotypes based on the imprinted regions showing defects [30, 34, 37–41]. As a consequence of this molecular and phenotypic heterogeneity, aggregating patients in groups is not always trivial.

Classical methods to identify differentially methylated regions and episignatures are usually based on inter-group comparisons, requiring a large number of samples in each group to reach statistically significant results [42, 43]. Those methods cannot be systematically applied in the context of rare diseases due to either the cohort size or the intra-group heterogeneity. It is especially the case when the disease affects only a handful of patients, hence making it difficult to gather cohorts large enough to satisfy canonical group-comparison method assumptions. In addition, group comparison loses the ability to capture inter-patient heterogeneity, such as in MLIDs. Therefore, single patient-based analyses could be used to address those issues and support the personalization of diagnosis.

In the literature, only two methods have been described for single case–control DNAm analysis. The first method is divided into two steps. First, the Crawford-Howell (C-H) adaptation of the t test is used to detect differential methylation at individual CpGs. Then, individual scores are aggregated in a DMR score using the Fisher aggregation method [44]. The second method [19] has been developed following two empirical rules: (i) at least 3 probes that each have methylation levels above the 99.9th percentile of the control distribution for that probe and are ≥ 0.15 above the control mean; (ii) at least 1 probe with a methylation level ≥ 0.1 above the maximum observed in controls for that probe.

Although both methods allow the detection of biologically relevant DMRs, they present some limitations. In the first method, the statistical method for individual probe testing described by Crawford-Howell is suggested to be used when the normative sample size (i.e., the size of the control population) is less than 50 [45]. Above that threshold, the Z-score is preferred. In addition to this limitation, the Fisher aggregation method used to combine individual scores (i.e., *P* values) assumes independence between variables. However, this assumption does not hold in most large high-throughput biology datasets that show a correlation between variables. Indeed, it has been shown that closely located CpGs tend to be co-methylated [46–48]. In the second method, the empirical rules, while relevant, do not allow the ranking of candidate regions by a confidence score such as a *P* value [19], and therefore it lacks the flexibility of applying a threshold for DMR calling. Finally, there is no evaluation of how the choice of the used parameters (e.g., number of probes, difference in methylation, cohort size) may affect DMR calling.

Therefore, in this paper, we propose a statistical method based on the Z-score followed by the Empirical Brown method that takes into account covariance between variables [49] to identify DMRs in a single-patient setting. First, we characterize the behavior of CpGs methylation status in various regions of biological interest and show that CpGs display a high correlation in those regions, thus justifying the use of Brown’s aggregation method to assign a DMR score. Second, we investigate how different parameters such as the size of the control population, the amplitude of the methylation difference, and the size of the regions affect the performance of this method for DMRs identification. In addition, we show the diagnostic utility of this method in the context of MLIDs and other neurodevelopmental disorders and congenital anomalies (ND-CAs), as well as its potential to identify new epivariants in existing datasets from a single-patient perspective.

## Results

### Characterizing CpGs methylation within a normal population

DNA methylation analyses are highly dependent on the control population used. Therefore, we decided to characterize the behavior of CpG methylation within our control population of 521 unaffected individuals. In the same way as DNA sequence variants, it is easier to infer the significance of an epivariant when it is in a region with a known function [50]. Thus, we focused on several regions known for their biological functions typically investigated in DNAm analysis: predicted CpG islands (CGIs); known imprinted regions; FANTOM5 enhancers; cis-regulating elements from the Encode project; genes associated with rare diseases from the Orphanet database (see Methods).

**Fig. 1:**
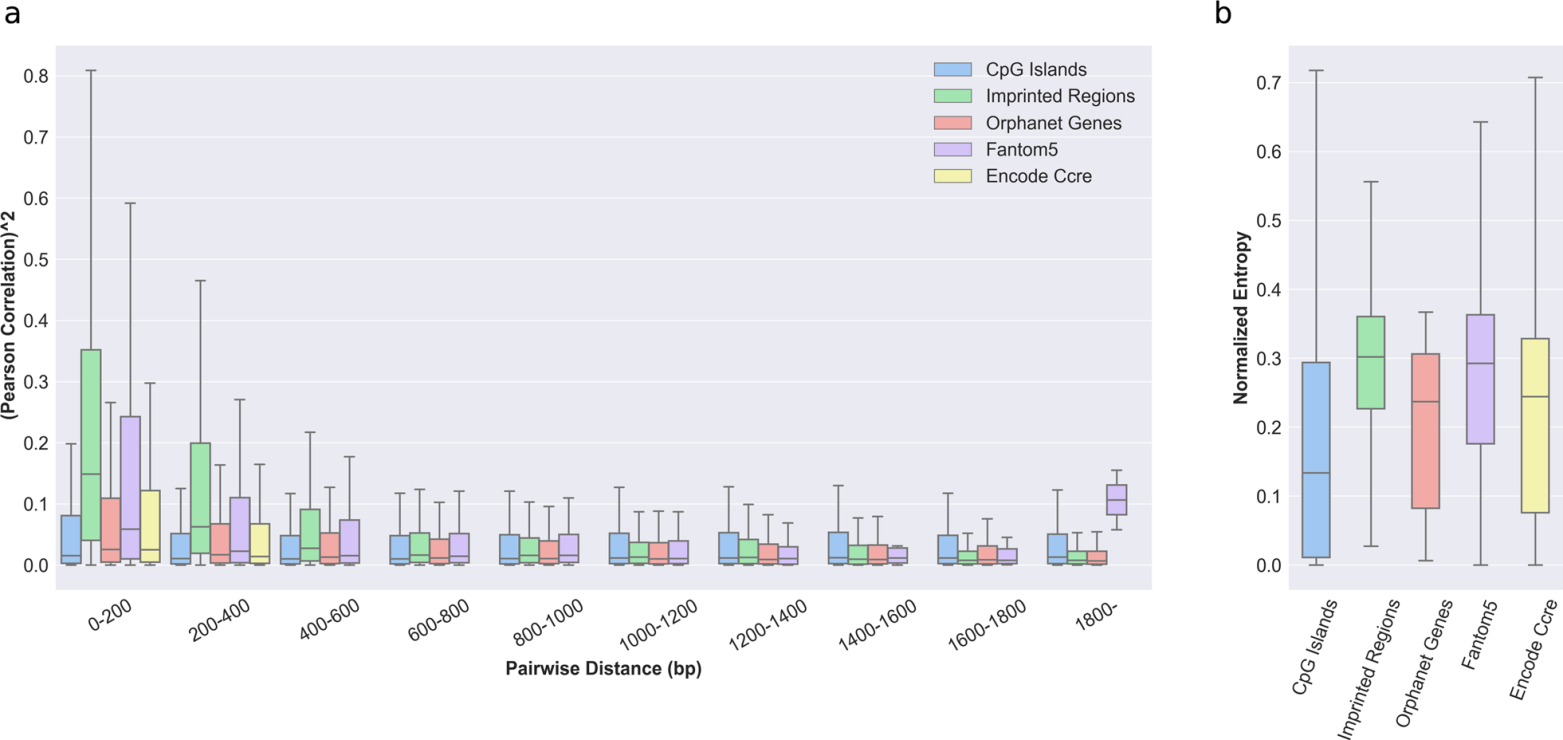
**a** Distribution of the pair-wise squared Pearson correlation for CpG pairs in different regions of biological interest as a function of the distance between the 2 CpG forming the pair. Correlation levels decline with distance in all regions of interest except in FANTOM5 enhancers where long-distance CpGs have a higher mean correlation than close-distance ones. **b** Distribution of the normalized Shannon’s entropy (between 0 and 1) per CpGs in regions of biological interest. CpGs within CpG Islands show the lowest mean entropy, whereas CpGs in the other regions have similar levels. Boxplot bars are limited to 1.5 × the interquartile range

First, we wanted to assess whether methylation between pairs of CpGs is correlated within those regions. Indeed, the canonical way to identify DMRs aims at aggregating *P* values of CpGs tested for differential methylation individually. As discussed previously, Fisher’s aggregation method has been the method of choice. However, this method assumes independence between variables. Thus, we computed the Pearson correlation across all the samples for CpG pairs in regions of biological interest as a function of the distance between the two CpGs forming the pair (Fig. 1a). It has been shown that closely located CpGs are co-methylated [46–48]. We confirmed that there is a larger proportion of highly correlated CpGs in the 0–200 bp range and that this proportion decreases with distance. However, we only noticed a sharp drop in mean correlation levels in imprinted regions and FANTOM5 enhancers whereas mean correlation levels in the other regions of interest stayed comparable. Interestingly, we saw a significant increase in correlation between CpG pairs in FANTOM5 enhancers that were separated by + 1800 bp. Enhancers can sometimes be separated by thousands of base pairs. We can only hypothesize that this change is related to a common DNAm regulatory mechanism for regions interacting together [46–48]. Furthermore, we showed that mean correlation levels were higher in imprinted (Mean *r* = 0.33) and enhancer regions (Mean *r* = 0.31), lower in CGIs (Mean *r* = 0.19), and close to zero in Orphanet genes (Mean *r* = 0.05) (Additional file 5: Fig. S1a).

In a second step, we investigated how methylation levels vary at the single CpG levels.

To do this, we calculated the standard deviation and Shannon’s entropy of the CpGs beta values (i.e., methylation percentage) within the same loci of interest (see Methods). Those two measures are complementary and indicate the stability of a given CpG’s methylation state within the normal population. The higher the entropy, the less stable the methylation level for the tested element. Our results indicate that CpG methylation levels are stable within the tested regions. Indeed, the mean standard deviation of the beta value is under 4% in all groups (Additional file 5: Fig. S1b). We also showed that overall mean entropy levels are low with CGIs having the lowest mean entropy (Mean entropy < 0.2), which highlights a high level of consistency in methylation (Fig. 1b). At the epigenome level, the mean entropy was 0.16, and the mean standard deviation was 3%. We could not detect any significant changes in those parameters at the chromosome level (Additional file 5: Fig. S2a and S2b). This highlights a stable distribution of methylation levels at the CpG level within our control population.

### Optimizing parameters for DMR identification in single patients

**Fig. 2:**
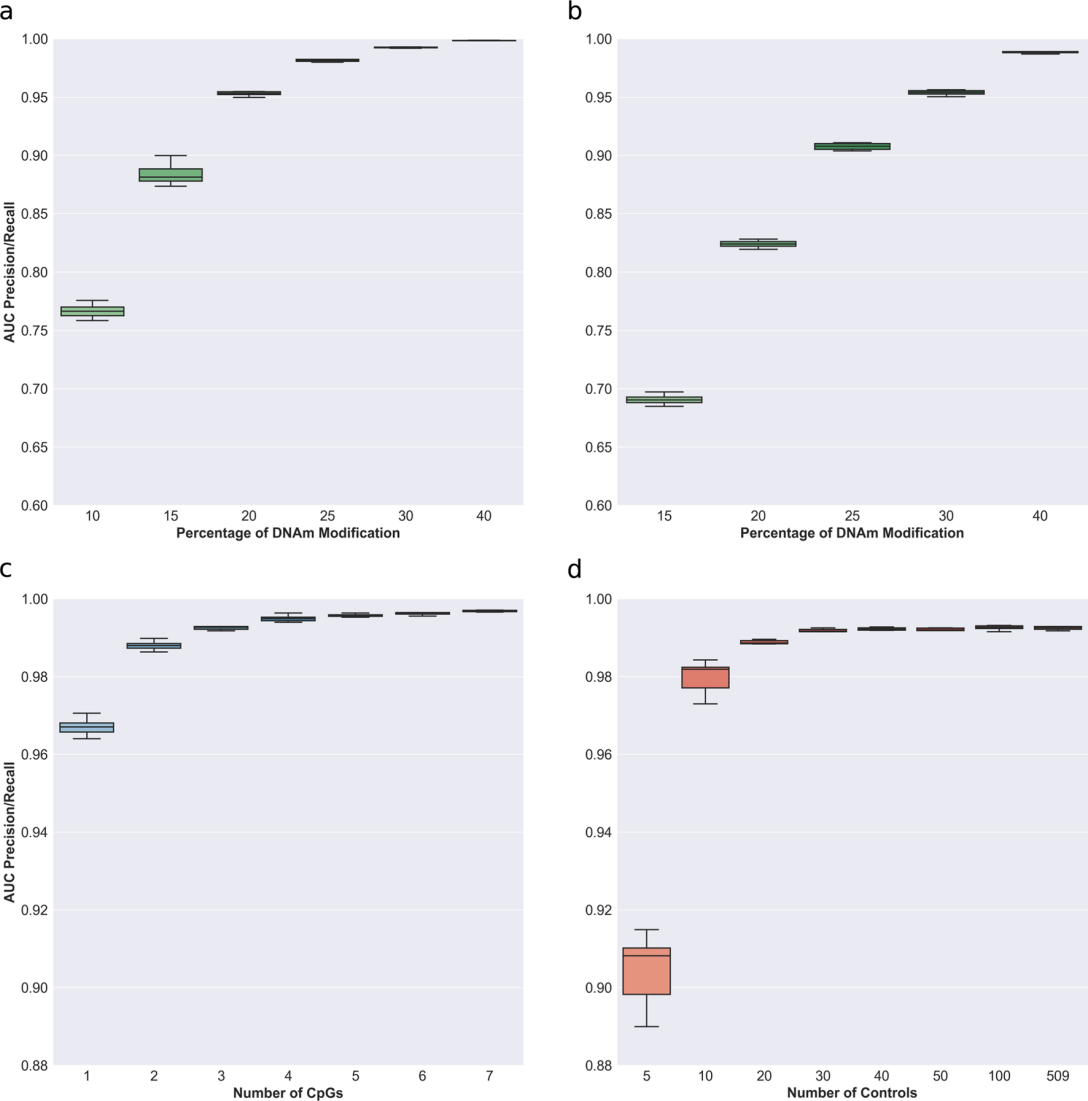
Areas under precision and recall curves to identify inserted DMRs (i.e., true positives). **a** Performances as a function of true methylation differences with a background noise of 5%. **b** Performances as a function of true methylation differences with a background noise of 10%. **C** Performances as a function of the number of CpGs for the background noise of 5% and 30% true methylation difference. **d** Performances as a function of the control population size for the background noise of 5% and 30% true methylation difference

As mentioned in the introduction, our method to assign a confidence score to differentially methylated regions (DMRs) consists of the Z-score, because our normative population was large (*N* = 521) and prevented the use of the Crawford-Howell method, in addition to Brown’s aggregation method to take into account the interdependence of the methylation level between adjacent CpGs. After defining the statistical bases of our method, we sought to assess how different parameters associated with DMR detection would influence the score of a region. Because of the difficulty to establish which signal is false in real data, we decided to use a semi-simulated approach based on a population of unaffected patients (see Methods). This strategy enabled us to define true DMRs and false signals that we considered as background noise and allowed the usage of standard performance metrics such as the area under the precision–recall curve (AUC) to evaluate the influence of several parameters. First, we tested how the difference in methylation levels between a sample of interest and the control population would affect the outcome of the scoring method. We performed this analysis on two datasets where we introduced either a low noise (5%, Fig. 2a) or high noise (10%, Fig. 2b) level. Then, in those noisy datasets, we assessed how the method performed to detect increasing true methylation differences. As expected, performances were poor when trying to detect a small methylation effect of only 5% relative to the noise (signal of 10%, low noise conditions mean AUC = 0.77; signal of 15%, high noise: mean AUC = 0.69). The method performed better when the methylation effect increases. Indeed, at 10% of relative methylation difference, the mean AUC for the low noise data and the high noise data were 0.89 and 0.83, respectively, and a mean AUC over 0.95 was obtained with a methylation defect of 15% for the data with low noise against 20% for the noisier one. In the subsequent analyses, we decided to use the low noise setting (5% of noise level) and introduced a 30% shift in methylation as a true signal to evaluate the influence of other parameters. Precision/recall curves as well as the AUC of the true and false positive rate are available in the supplementary data (Additional file 5: Figs. S3, S4).

Next, we assessed how the number of modified CpGs within a window would affect its score. Indeed, in the literature, it is commonly accepted to use windows of 1000 bp containing a minimum of 3 CpGs when looking for DMRs [19, 44, 51, 52]. However strong arguments for the choice of this parameter are lacking. Therefore, we evaluated the detection of DMRs using windows of increasing size, from 1 to 7 CpGs (Fig. 2c, Additional file 5: Fig. S5). While the AUC for precision and recall was high for all window categories (> 0.96), performances tended to increase with the number of CpGs and approached a plateau around 0.995 when the number of CpGs per window was ≥ 4.

Finally, we tested how the size of the control population would influence performance by comparing the semi-simulated data against a population of increasing size, from 5 to 509 samples (Fig. 2d, Additional file 5: Fig. S6). Although the overall performance was good (AUC > 0.89), we could observe significant improvements when the size of the control population increased significantly until 30 controls, the highest differences were seen from 5 to 10 controls (mean AUC from 0.904 to 0.980) and from 10 to 20 controls (mean AUC from 0.980 to 0.988). Larger control populations displayed a lower increase in performances with this high signal–noise ratio (30% signal, 5% noise) setting.

### Identification of DMRs in Beckwith–Wiedemann patients

**Fig. 3:**
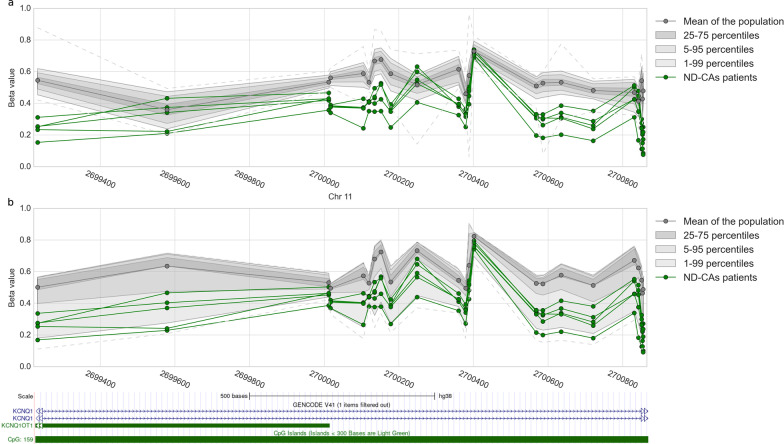
Methylation profile of the region KCNQ1OT1:TSS-DMR (hg38: chr11: 2,699,200–2,700,855) in the 5 BWS patients and the controls. **a** Controls (*N* = 521) from the literature used for Z-score calculation. **b** Controls (*N* = 27) produced from the same study used for the C–H test

After defining optimum parameters, we sought to evaluate the performance of the method for DMR identification on real patient data. We performed the methylome analysis on 5 patients suffering from Beckwith-–Wiedemann syndrome (BWS) that also showed multilocus imprinting disturbances (BWS-MLID, GEO accession number GSE133774, and GSE153211). Because controls (*N* = 27) from the same batch were available we decided to compare the scoring of DMRs using the Crawford-Howell method with batch-matched controls and the Z-score with a larger population of controls (*N* = 521) from another batch (GEO accession number: GSE152026). This allowed evaluation of whether in the context of single-patient analysis, one should gather a small control cohort (*N* < 50) obtained at the same facility or whether using a larger cohort of publicly available controls would yield better results. Nevertheless, we used a modified version of BMIQ [53] described in [9] to rescale methylation value distribution between patients and controls to reduce batch effects (see Methods). Rescaling efficiency was evaluated by looking at the probes’ methylation level distribution (Additional file 5: Fig. S7). Both our patient and control populations came of European descent; therefore, we did not expect to find DMRs related to ethnicity. To limit the number of false positives that may occur due to DNAm changes associated with ethnicity, age, BMI, and smoking status, we compiled a list of CpGs influenced by those covariates and removed DMRs that included them (see Methods). To compare the two tests, we investigated the aggregated *P* value of known imprinted regions and checked whether the regions detected with our method were also retrieved in the original papers [30, 39] (Additional file 1: File S1, Table 1). Across the 5 samples and out of the 43 imprinting loci tested in the original paper, we found 22 to be under the 0.05 corrected *P* value significance threshold with the Crawford-Howell method, versus 49 with the Z-score, 19 DMRS identified with one method were also significant using the other (representing 86% of the 22 found with C-H and 39% of the 49 found with the Z-score). Those numbers were lower at the 0.01 threshold (Number of regions: C-H = 19, Z-score = 46) for 17 DMRs deemed significant by both methods. One of the typical molecular defects of BWS involves loss of methylation at the KCNQ1OT1:TSS-DMR locus and normal methylation at H19/IGF2 IG-DMR; this pattern was identified in the patients in the original papers through molecular testing. Using our single-patient approach, the KCNQ1OT1:TSS-DMR locus was considered as significantly differentially methylated (*P* value < 0.01) in all patients only when using the Z-score, suggesting a higher sensitivity in comparison to the Crawford-Howell test. Visualization of the profile of methylation levels in that region showed that this result is due to the high variability of the small control population used for the C-H test (Fig. 3). In addition to this locus, several other known imprinting regions were found as significantly differentially methylated in the patients using the Z-score, thus confirming the MLIDs diagnostic previously established, and the capacity of the method to identify regions of interest (Additional file 2: File S2).

Then, we performed a scan of the microarray-based epigenome of the BWS patients to identify new DMRs outside of canonical regions. We analyzed only windows containing at least 4 CpGs (see Methods) as this provided the optimal performances on simulated data and applied a strict threshold of 10% on the median difference between the patient CpGs methylation level and the mean methylation in the control population. Except for one patient (GEO accession number: GSM4635795) where we found 107 DMRs, we identified less than 10 DMRs in the other patients for a total of 143 additional significant DMRs (Additional file 3: File S3, Additional file 5: Fig. S8 a-f). Out of those, 2 regions were found hypermethylated in all patients, encompassing the ABCD1P4 (NCBI entry: 26,957) pseudogene and the AC093787.2 long non-coding RNA promoters. Additionally, one DMR in the CpG Island within the protein-coding gene TNNT3 (NCBI entry: 7140) was found in 4 of the patients and 4 DMRs were found in two patients, two of those in the protein-coding genes COL18A1 (NCBI entry: 80,781) and ANK1 (NCBI entry 286). Interestingly, among the DMRs identified outside canonical imprinting regions, some were located in genes with known relationships to congenital and neurodevelopmental diseases, and further investigation would help to better characterize the impact of DMRs in such genes. To further evaluate the influence of age on the predicted DMRs, we performed the same analysis using different age-based subgroups of the control population. We couldn’t detect any major differences in the number of DMRs identified with the different control subgroups (Additional file 5: Fig. S9a and S9b). We finally used the DNAm age clock [9] to control methylation age in the patient population (see Methods) and to check if methylation alterations could affect age estimation. We found that all patients had a methylation age close to their biological age (< 8.5 years) (Additional file 1: File S1, Table 2).

### Identification of DMRs in ND-CAs patients

**Fig. 4:**
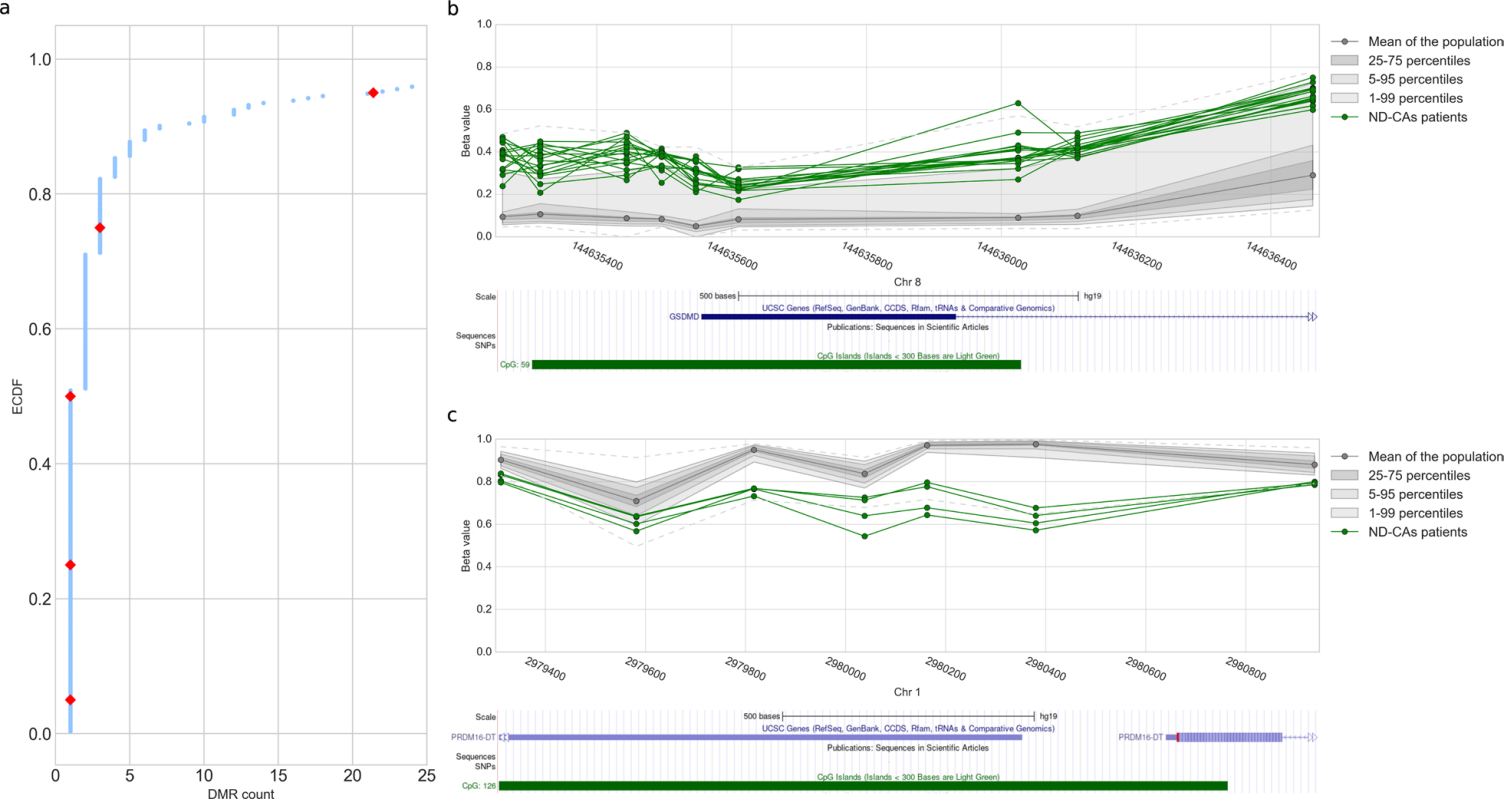
**a** Empirical cumulative distribution function (ECDF) representing the percentage of samples having less than a certain number of DMRs. Percentiles 5, 25, 50, 75, and 95 are represented in red. Samples with > 25 DMRs are not shown. **b** Recurrent hypermethylation of the GSDMD locus (hg19: chr8: 144,635,260–144,636,462) and associated UCSC Genome Browser view. **c** Recurrent hypomethylation at the PRDM16 locus (hg19 chr1:2,979,311–2,980,937) and associated UCSC Genome Browser view

To further evaluate our method, we applied the same analysis procedure to methylation data from 489 individuals suffering from neurodevelopmental disorders (NDs) and congenital anomalies (CAs) described in [19]. DMRs were originally identified in that cohort using the empirical method described in the introduction. Using our method, we found a total of 4261 DMRs in 293 patients (i.e., 60% of the patients tested), with most patients having less than 3 DMRs (percentile 75) (Fig. 4a). Similarly to the original paper, we removed samples with more than 10 DMRs. Doing so yielded 520 identified DMRs in 268 patients (i.e., 55% of the patients tested), 53 of those DMRs were already described in the original paper (i.e., 37% of DMRs identified in [19]) (Additional file 4: File S4). Among the 520 identified DMRs, 272 were present in at least two samples (i.e., 52% of the total in patients with less than 10 DMRs), mapping to 79 genes. At the gene level, we identified 32 DMRs in genes affected in more than two samples (representing 41% of all the affected genes). The most affected gene was GSDMD which shows significant hypermethylation in 17 patients (Fig. 4b), the second one was ECEL1P2 a pseudogene hypomethylated in 15 patients. GSDMD has been linked to neonatal-onset multisystem inflammatory disease (NOMID) in mice [54]. According to the rare disease database, NOMID symptoms include cognitive disabilities. No existing data point to disease association in the case of ECEL1P2. Interestingly, we could detect a DMR in the gene PRDM16 (Fig. 4c), a gene shown to be involved in cardiomyopathy [55], which was consistent with the symptoms experienced by the patients (i.e., GEO ID: GSM2366439, GSM2366759, GSM2366459, GSM2366724).

## Discussion

In the context of rare disorders affecting the epigenome, classical case–control studies are not always applicable. In addition, it has been shown that individuals with overlapping phenotypes suffering from multilocus methylation disturbances (MLMDs) show unique methylation patterns that could be used to further refine the clinical diagnosis [16, 21, 23, 56–58]. Previously, two methods have been proposed to detect aberrant methylation in cases using a single-patient approach with one of them based on statistical testing [19, 44]. In this paper, we built on those previous methodologies to propose an apprehensible single-patient-based method for DNA methylation analyses. First, we confirmed previous findings that methylation levels of CpGs within close distance are correlated [46–48] and observed a constant decrease in correlation with distance (Fig. 1a). We further showed that there were positive mean correlation levels between CpGs located within CpG islands, known imprinted regions, FANTOM5 enhancers, cis-regulating elements from the Encode project, and to a lesser extent in genes associated with rare diseases from the Orphanet database (Additional file 5: Fig. S1a). Those results indicate that the assumption of independence made by Fisher’s method does not hold and should be replaced by a method taking this interdependence into account when aggregating scores of individual CpGs into DMRs. We suggest the use of Brown’s aggregation method implemented in [49]. We further showed that CpGs in a population of unaffected individuals have high stability as illustrated by the low entropy and standard deviation observed (Fig. 1b, Additional file 5: Fig. 1b). That stability was seen throughout the entire epigenome (Additional file 5: Fig. S2a and S2b). This low variability can have an impact on DMRs identification capacity. Indeed, extremely low standard deviation values can be caused by a bad sampling of the normative population, which can lead to extremely significant scores for a CpG even if the difference in methylation is low. Thus, we used semi-simulated data to quantify how methylation difference, amongst other parameters, would influence the performance of DMR identification. The method showed satisfying performances when the methylation difference in the DMRs was at least 10% both for low and high noise data, but better performances were achieved at a 15% difference and above (Figs. 2a and 2b). Therefore, we advise applying a threshold of at least 10% on the median difference in methylation between the controls and the case when considering DMR significance. In addition to the effect size, we investigated the influence of the number of CpGs per window. Common DMRs identification methods use windows of at least 3 CpGs [19, 44]. Precision–recall AUCs starting at one CpG were already in a very good range, and we observed increasing performance until a peak that plateaued at 4 CpGs (Fig. 2c). We concluded that every window size tested (≥ 1 CpG) is acceptable in terms of performance but warn about the analysis burden that smaller window size generates. We thus decided to use ≥ 4 CpGs for the subsequent analyses. Then, we tested for the minimum number of samples that should be included in the control population (Fig. 2d). Based on our results, we suggest using a control population of at least 30 samples. However, due to sampling bias, we believe that a larger control cohort will generally yield fewer false positives. Nevertheless, our semi-simulated data present some limitations. Indeed, we could not account for batch effects that are present when using a different cohort as controls, and the way we modeled DMRs may not reflect the full field of biological variations occurring in various syndromes. In addition, our analysis was based on a strong signal of 30% to find the best value for the size of the control population and the number of CpGs per window. We seldom encountered DMRs with a signal as strong as 30% in patients’ data and thus speculate that the measured performances using semi-simulated data are probably overestimated. However, trends in those performances are still a good indication that a larger control population size and number of CpGs per window will yield better results, hence our suggestion to use a control population as large as possible, and focus on windows containing at least 4 CpGs. We also compared the use of a batch-matched cohort (*N* = 27) against a larger cohort (*N* = 521) from another batch, using methylation data from 5 patients diagnosed with Beckwith–Wiedemann syndrome and MLIDs. In the context of the two control populations used here, we showed that using the Z-score with a larger cohort outperformed the Crawford-Howell t test with a smaller—although batch-matched—control population in the ability to retrieve the hypomethylation of the KCNQ1OT1 region (Fig. 3). However, we want to underline the necessity to correct the batch effect before this comparison. We rescaled the global distribution of methylation levels using an adapted version of the BMIQ software [9, 53] (Additional file 5: Fig. S7). This method allows the use of a golden standard to rescale new samples and thus is very well suited to single-patient analysis, where individual samples can all be normalized against the same standard. Using this method allowed us to improve greatly the outcome of DMRs analysis for the non-matching batch cohort. Furthermore, it has been shown that different covariates may affect DNAm. Accounting for those covariates is trivial when using multivariate linear models, as one can simply include them in the design matrix [43]. However, this is not possible in a single-patient analysis. We believe that a careful match should always be made between the control cohort and the patient to avoid covariate effects as much as possible and that this task will be easier in the future with the greater availability of methylation data. However, at the time of this study, only one large population of control produced with the EPIC microarray was available (GEO accession number: GSE152026). To account for age, we showed that our patient methylation age was similar to their biological age and that controlling for age in the control population had little effect on the number of DMRs detected (Additional file 5: Fig. S9a and S9b). To control for other covariates such as smoking status, ethnicity, and BMI, we decided to compile a list of CpGs known to be correlated with different covariates and removed all DMRs containing them. Finally, we applied this method to detect additional DMRs in the same patients at the whole epigenome level (Additional file 5: Fig. S8). We were able to identify DMRs that were not reported previously. Among those new differentially methylated regions, two were present in all BWS-MLID patients and implicated genes that should be studied further in the context of BWS-MLID. In addition, we applied our method to a previously described cohort of 489 undiagnosed NC-DA patients. Similarly, we identified new DMRs of interest in several patients. Although additional research would be needed to assign any role to those DMRs in the symptoms experienced by patients, we believe that our method of analysis allowed a greater characterization of their DNA methylation landscape and showed promising results to understand the molecular mechanism at play.

In conclusion, we described an improved single-patient-based method to detect differentially methylated regions and discussed its optimal parameters to increase its utility and reliability in a diagnostic setting.

## Methods

### Cohorts

Illumina EPIC data were retrieved for GSE152026, GSE133774, and GSE153211. IDAT files were available for GSE133774 and GSE153211. We used R *minfi* package to preprocess them. Cross-reacting probes, probes containing SNPs, and probes with a detection *P* value > 0.01 were removed according to *minfi* functions, and samples were normalized using *minfi* quantile normalization. Probes from sexual chromosomes were removed from the analysis, resulting in 830,257 probes left. Beta values from Illumina 450 k data of GSE89353, GSE36064, GSE40279, GSE42861, and GSE53045 described in [19] were retrieved. Only the 370,065 overlapping probes were used for the analysis. Beta values were rounded to 3 digits. Rounded beta values were used for batch correction (see Batch correction). Logit-transformed Beta values (= M values) were used for all statistical analyses. For consistency with the annotation provided by the manufacturer, genome versions hg38 and hg19 were, respectively, used for the annotation of Illumina EPIC and 450 k data.

### Characterizing CpGs within a normal population

521 control patients from GEO datasets GSE152026 were used to characterize probes present on the Illumina EPIC array. Several annotation files in bed format were retrieved from the UCSC table browser using the hg38 version of the genome. Those annotations included Orphanet genes [15, 59], CpG Islands (This track was generated using a modification of a program developed by G. Miklem and L. Hillier (unpublished)) and Encode candidate cis-regulation elements (based on ENCODE data released on or before September 14, 2018) [60]. CpGs within imprinted regions were selected based on the research in [61]. FANTOM5 enhancers were downloaded from the Zenodo database [62].

Correlation between pairs of CpGs was calculated using Pearson’s correlation. We used Fisher’s z-transformation to calculate mean correlation: individual correlation coefficients were transformed in Z-scores before mean calculation, then mean Z-scores were transformed back into mean correlation. Shannon’s entropy was calculated using the *entropy* function in Python *scipy.stats* package, and the *histogram* function from the *numpy* package, by binning CpGs beta values in 10 bins from 0 to 1, and default parameters. Entropy was normalized to vary between 0 and 1.

### Semi-simulated data

Generation of control population datasets, windows, beta value shifts, *P* value per window, and performance parameters was made using in-house Python3 scripts and the libraries numpy, scipy, pandas, statsmodels as well as in-house R 4.1.1 scripts with packages reshape2 and data.table. We selected 10 random samples from the control population (GEO accession number: GSE152026) to be modified and compared to the rest of the controls. To evaluate the influence of the size of the control population the remaining control population was progressively divided into smaller datasets (*N* = 100, 50, 40, 30, 20, and 10). We defined windows of CpGs using the Illumina v1.0 B5 annotation from the Illumina website (https://emea.support.illumina.com/downloads/infinium-methylationepic-v1-0-product-files.html). ChrX, ChrY, ChrM, and individual probes with missing information about chromosomes, positions, or strands (hg38 version) were removed. Adjacent probes were aggregated into non-overlapping windows using a fixed number of CpGs and a maximum window size of 1000 bp. The number of CpGs per window ranged from 1 to 7 to assess the influence of window size, otherwise, it was 4. To avoid any effect due to genomic location, chromosomes were segmented into 1000 regions of equal size: 1/10 of these regions were selected for modification. Windows overlapping these regions were selected for modification. Selected windows with missing beta values (for at least one probe and at least one control) were removed (Additional file 5: Table S1). To mimic noise in our data, a shift in beta value was applied to the probes in all regions. The shift applied (x %) per probe was sampled from a Gaussian distribution with mean = x, and std = 0.5%, and was either low (x = 5%) or high (x = 10%). The same principle applies when the signal (5, 10, 20, 30, or 40% of methylation) was inserted for DMR identification and evaluating effect size. To avoid negative beta values and beta values over 1, we added or subtracted signal when the beta value was over and under 50%, respectively.

### Batch correction

A batch correction was applied through a rescaling of the distribution of beta values using the adapted BMIQ [53] function described in [9] with default parameters except nfit = 820,000 (BWS analysis) or nfit = 415,000 (ND-CAs analysis) and th1.v = c(0.10, 0.60). Rescaling is made in function of a reference sample. For the BWS analysis, reference was either defined by the mean beta value of samples from GSE152026 (when testing with the z-score) or GSE153211 (when testing with C-H). For the ND-CAs samples, the reference was the mean beta value of samples from GSE42861. All were normalized by those two references in the respective analysis.

### DNAm age calculation

DNAm age of the BWS patients was calculated using the software described in [9].

### DMR identification

Individual CpGs in BWS and ND-CAs samples were tested individually for differential methylation using either a two-tailed Z-score or a two-tailed Crawford-Howell t test [45] against a control population using the Python scipy.stats library. *P* values obtained from Z-score were adjusted for multiple testing by the Bonferroni method (using the array size as the number of tested CpGs). DMRs were defined by a rolling window approach of 1000 bp containing at least 4 CpGs, and overlapping windows were merged. *P* values for CpGs within the same window were aggregated using Brown’s aggregation method described in [49]. Significant DMRs were defined as having an aggregated *P* value > 0.01 and a median difference in methylation of 10% with respect to the controls. Statistical testing was always performed on M values and not beta values due to their statistical properties. A “black list” of CpGs known to be involved in BMI [11, 12], aging [9], smoking status [13], and ethnicity [10] was compiled (Additional file 1: File S1, Table 3). DMRs containing any of those CpGs were removed. To assess the effect of age on DMRs identification in BWS patients, we subdivided our control population into three age categories: under 25yo (mean age = 21.4yo), between 25 and 50yo (mean age = 37.8yo), and over 50yo (mean age = 56.8yo). The mean age of the total control population was 38.7yo.

## Supplementary Information

**Additional file 1**. Score of imprinted regions of BWS patients; DNAm age; “blacklist” of CpGs. 

**Additional file 2**. DNAm profile of imprinted regions in BWS patients.

**Additional file 3**. List of DMRs identified in ND-CA patients.

**Additional file 4**. List of DMRs identified in BWS patients.

**Additional file 5**. Supplementary Figures and Tables. Boxplots for mean Pearson correlation, standard deviation and entropy; table of semi-simulated DMRs; AUC and precision/recall curves; normalization of BWS patients; DMRs identified in BWS patients; influence of age on DMR identification.

## Data Availability

The data supporting the findings are available within the article and its supplementary materials. Public datasets used in the study are described in the Method section.
