# Supplementary figures and images for: Identification of differentially methylated regions in rare diseases from a single-patient perspective
Source: Clin Epigenetics. 2022 Dec 16;14:174. doi: 10.1186/s13148-022-01403-7 (PMC9758859; doi:10.1186/s13148-022-01403-7)

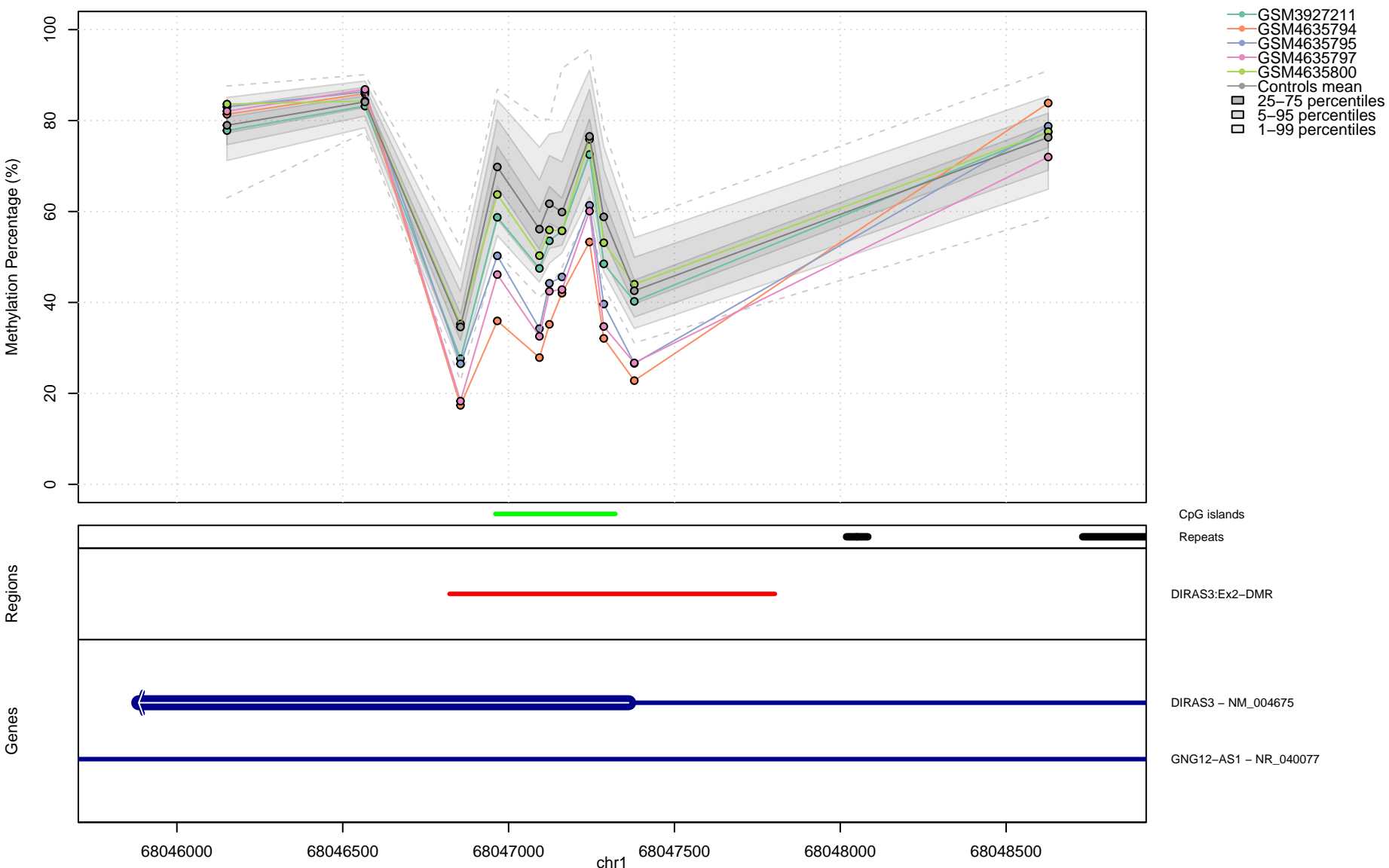

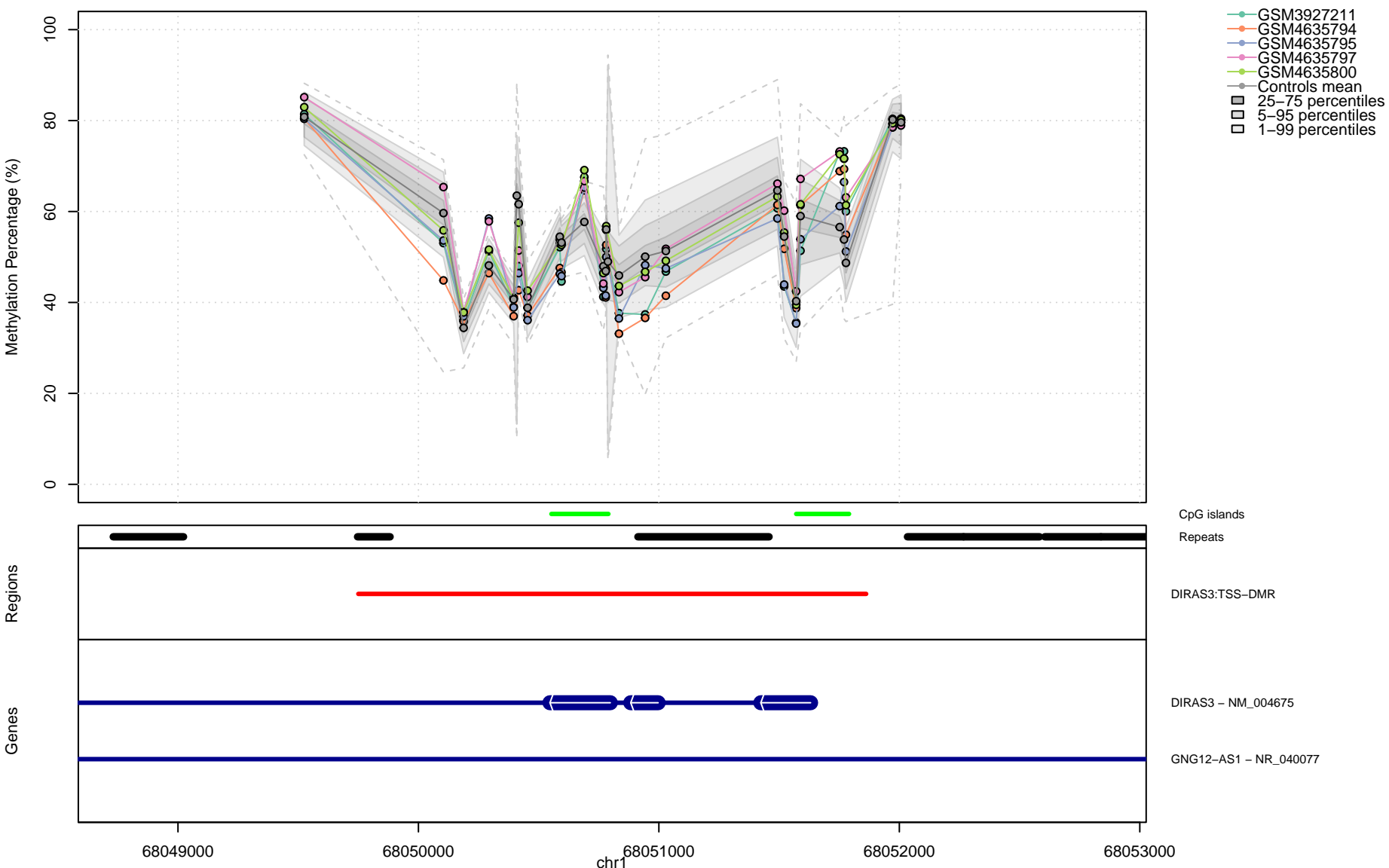

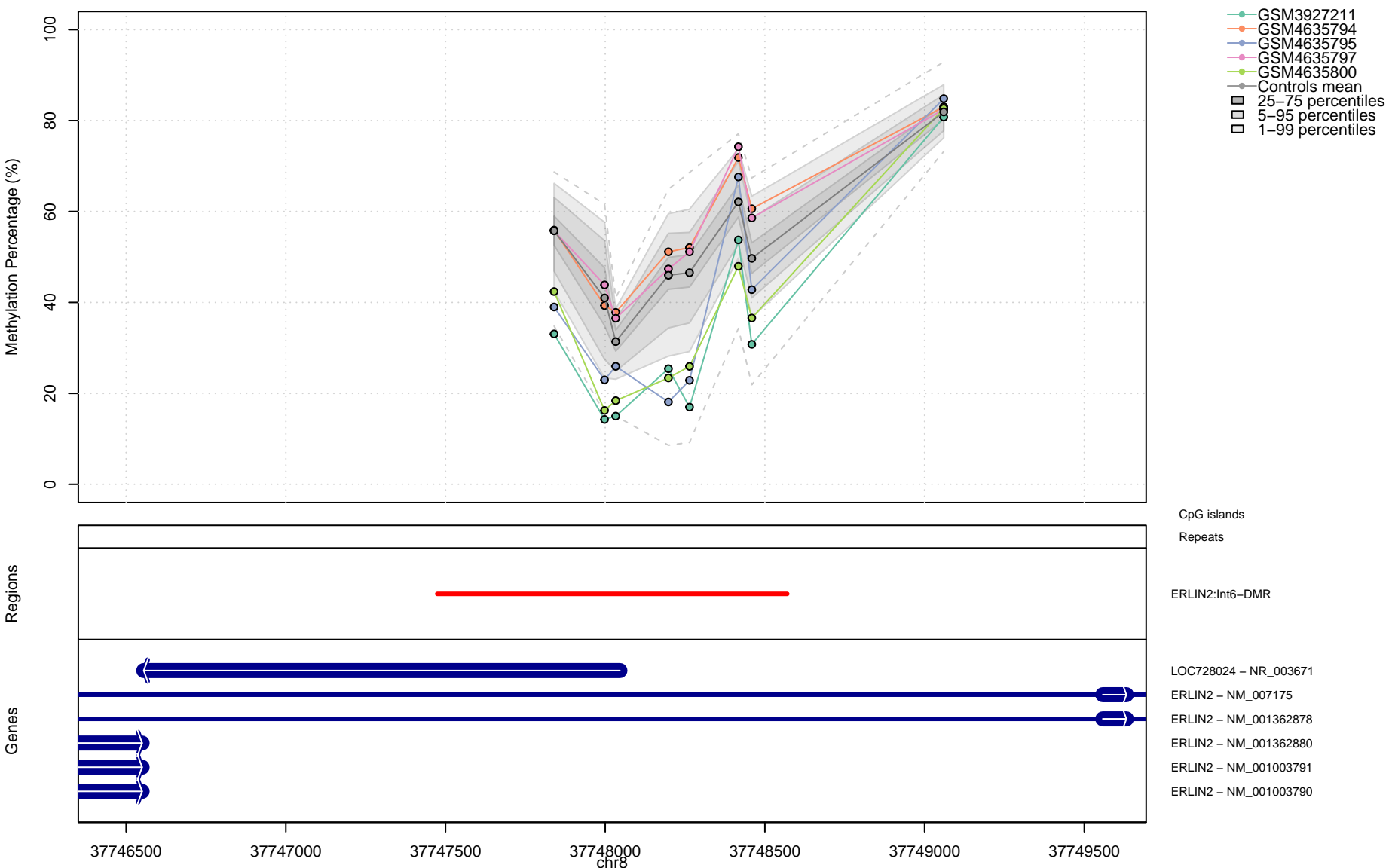

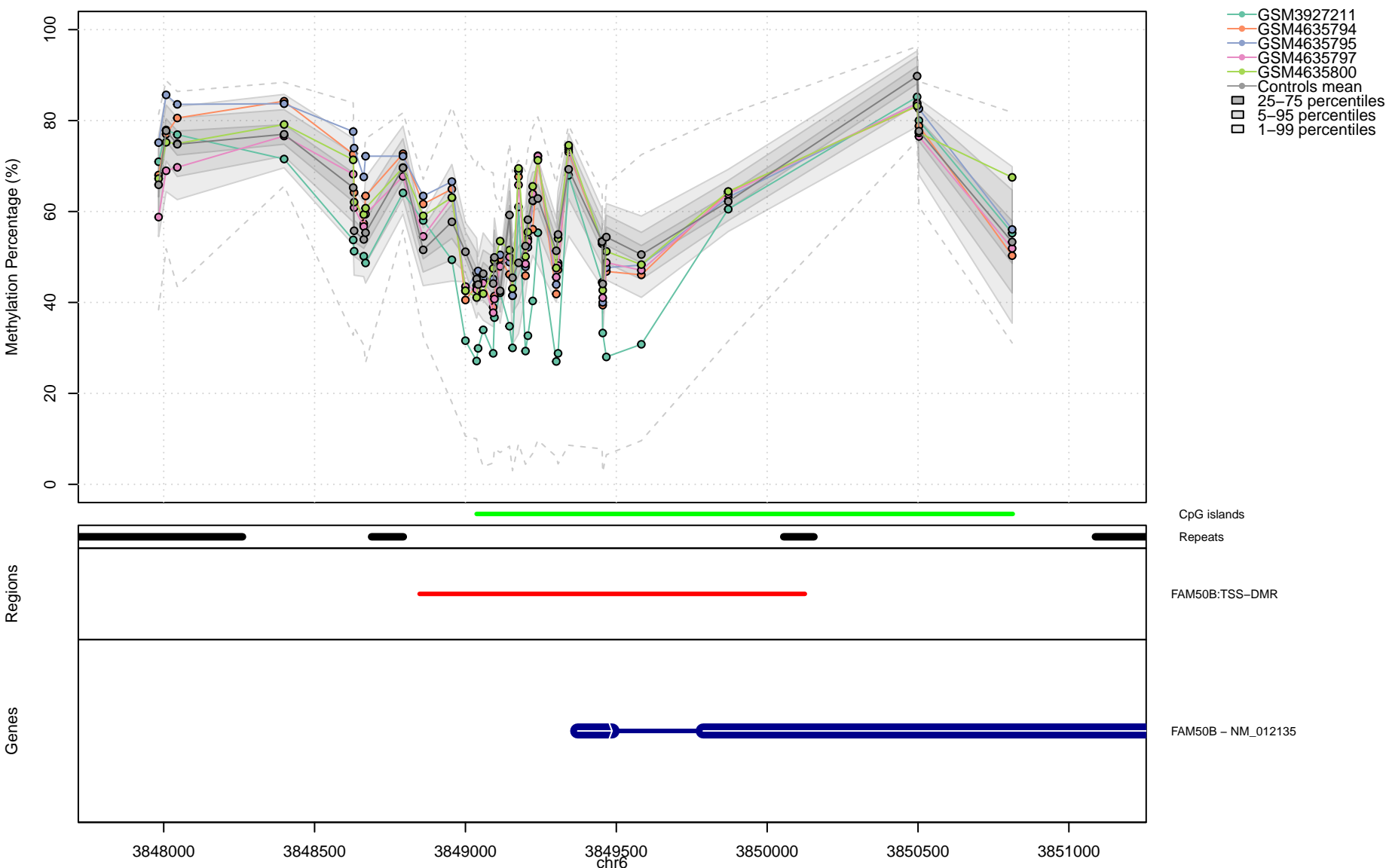

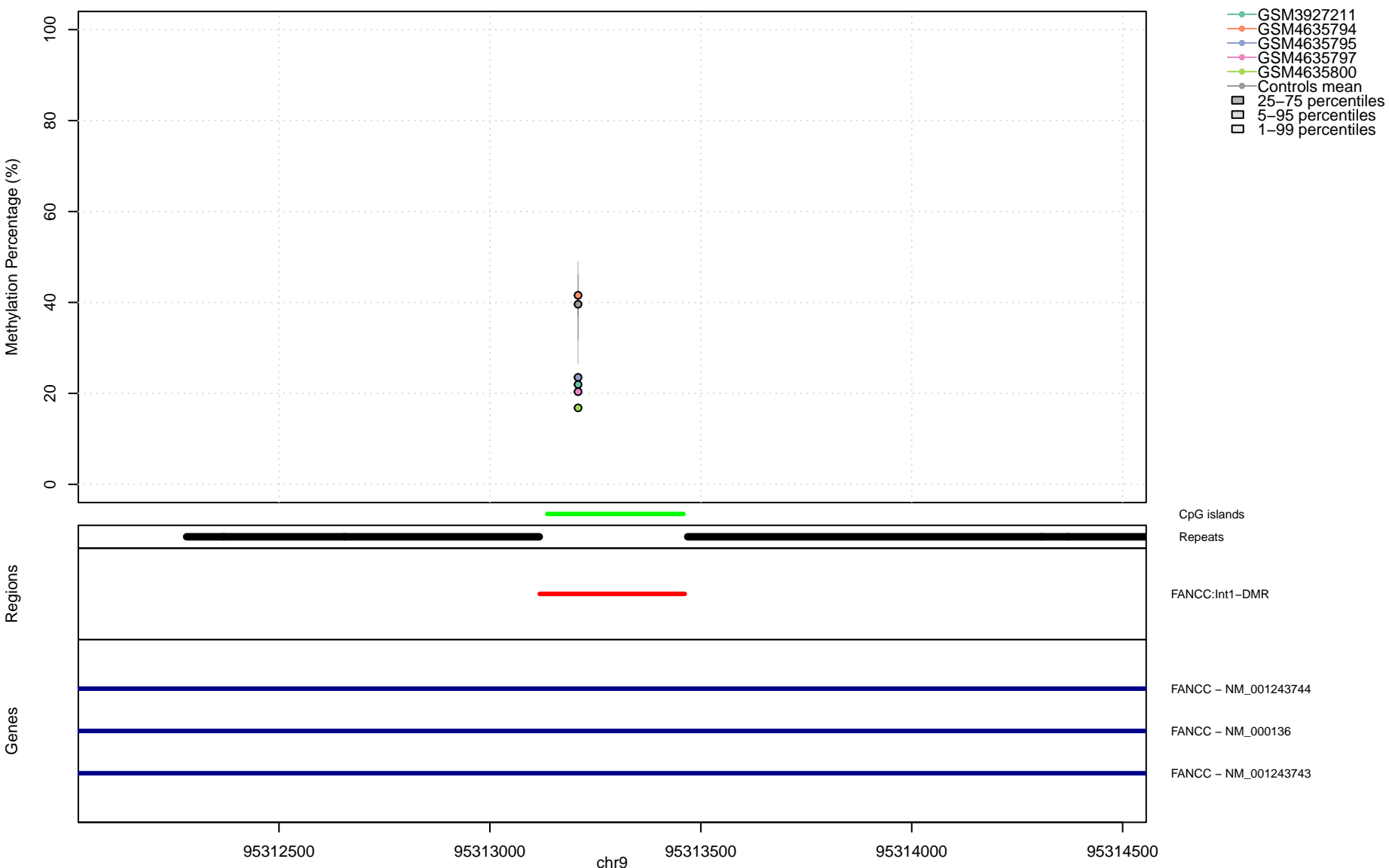

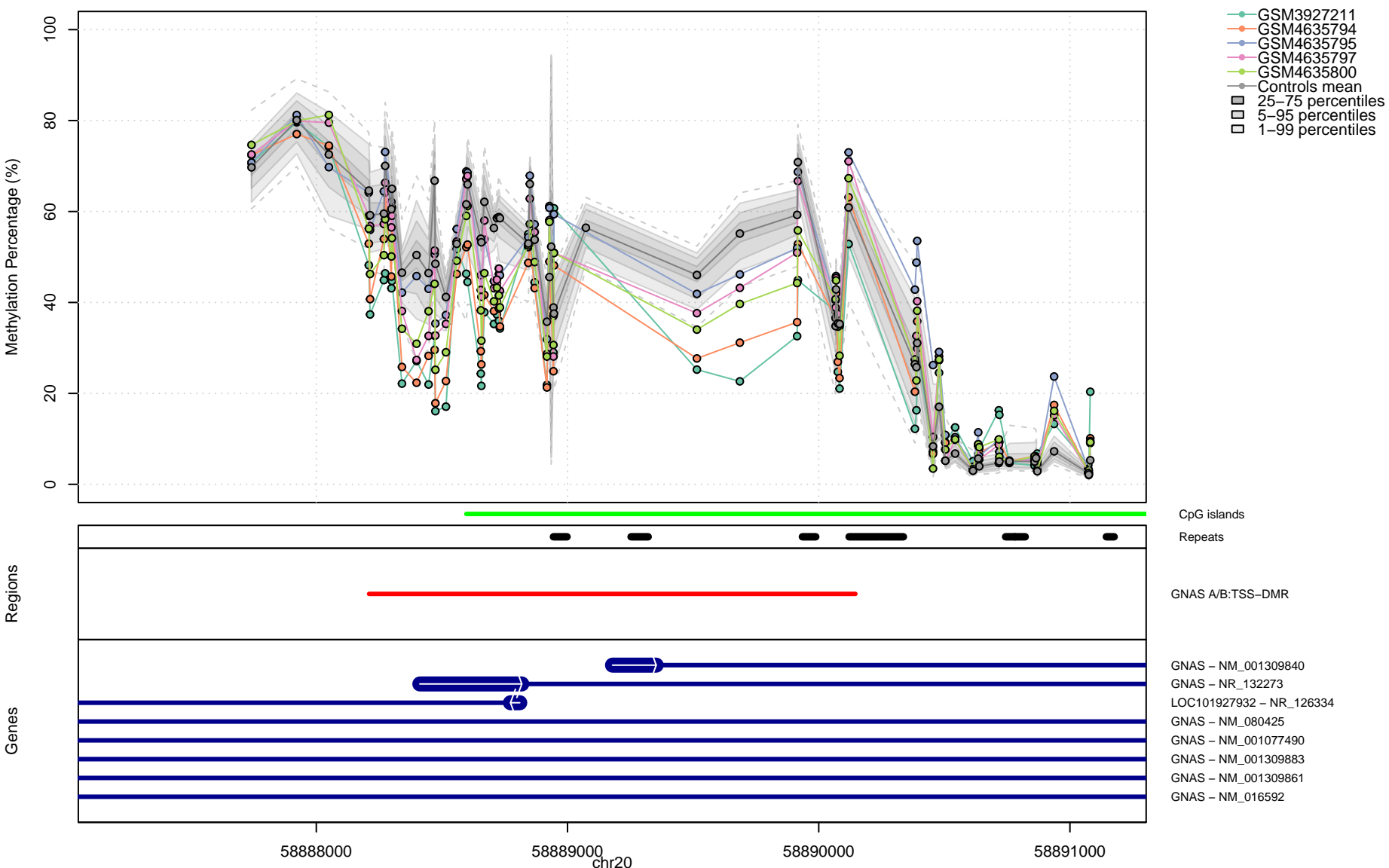

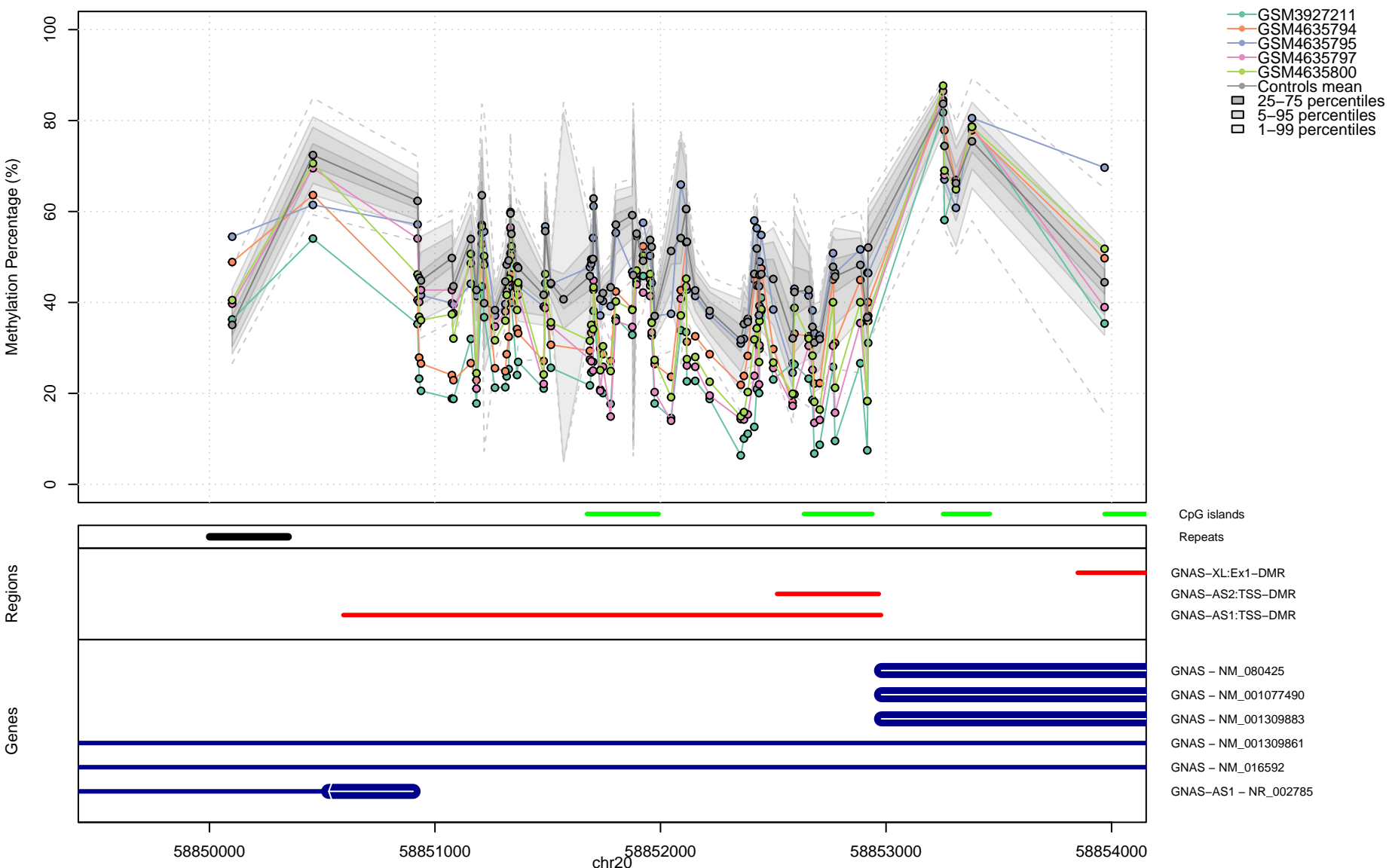

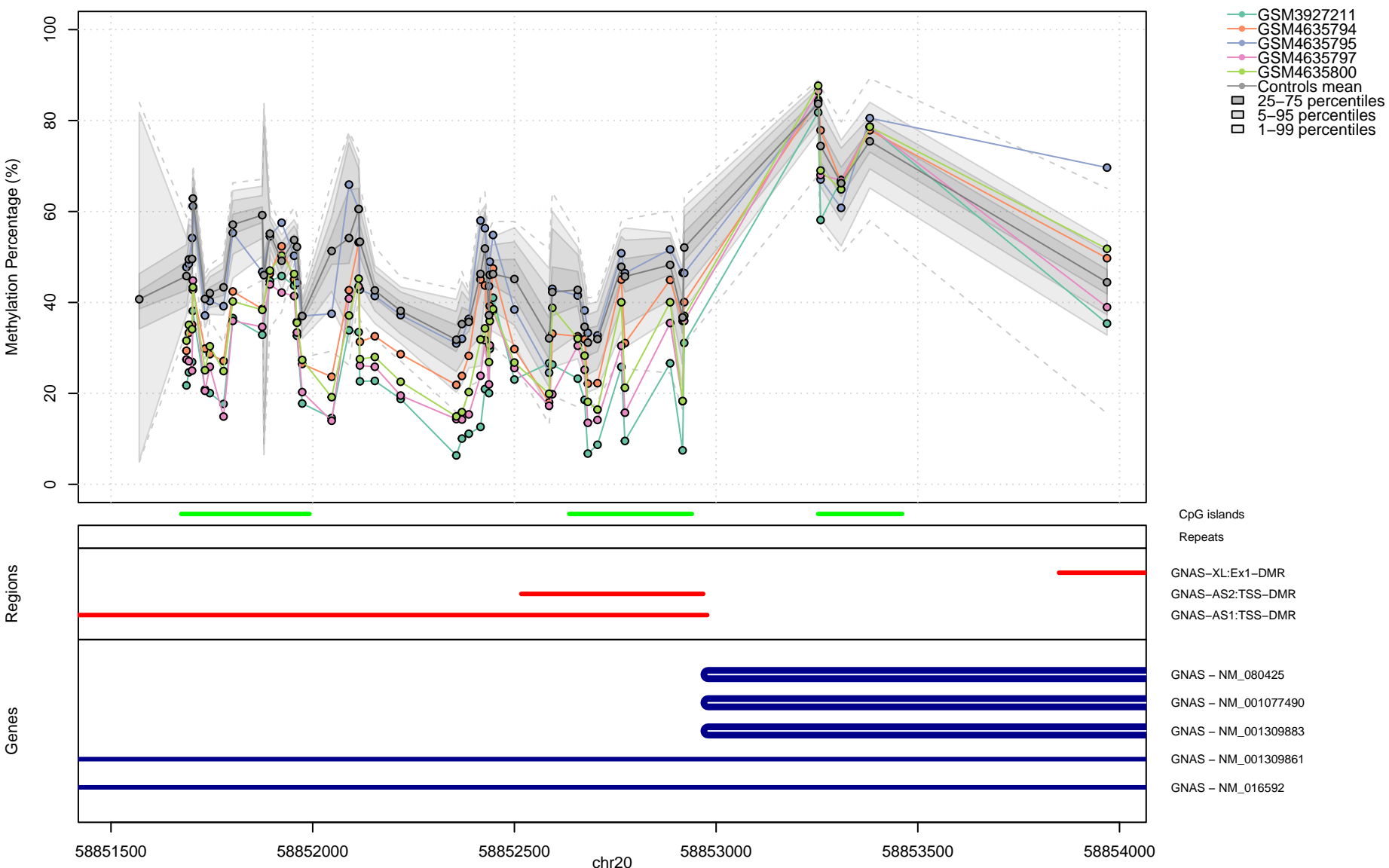

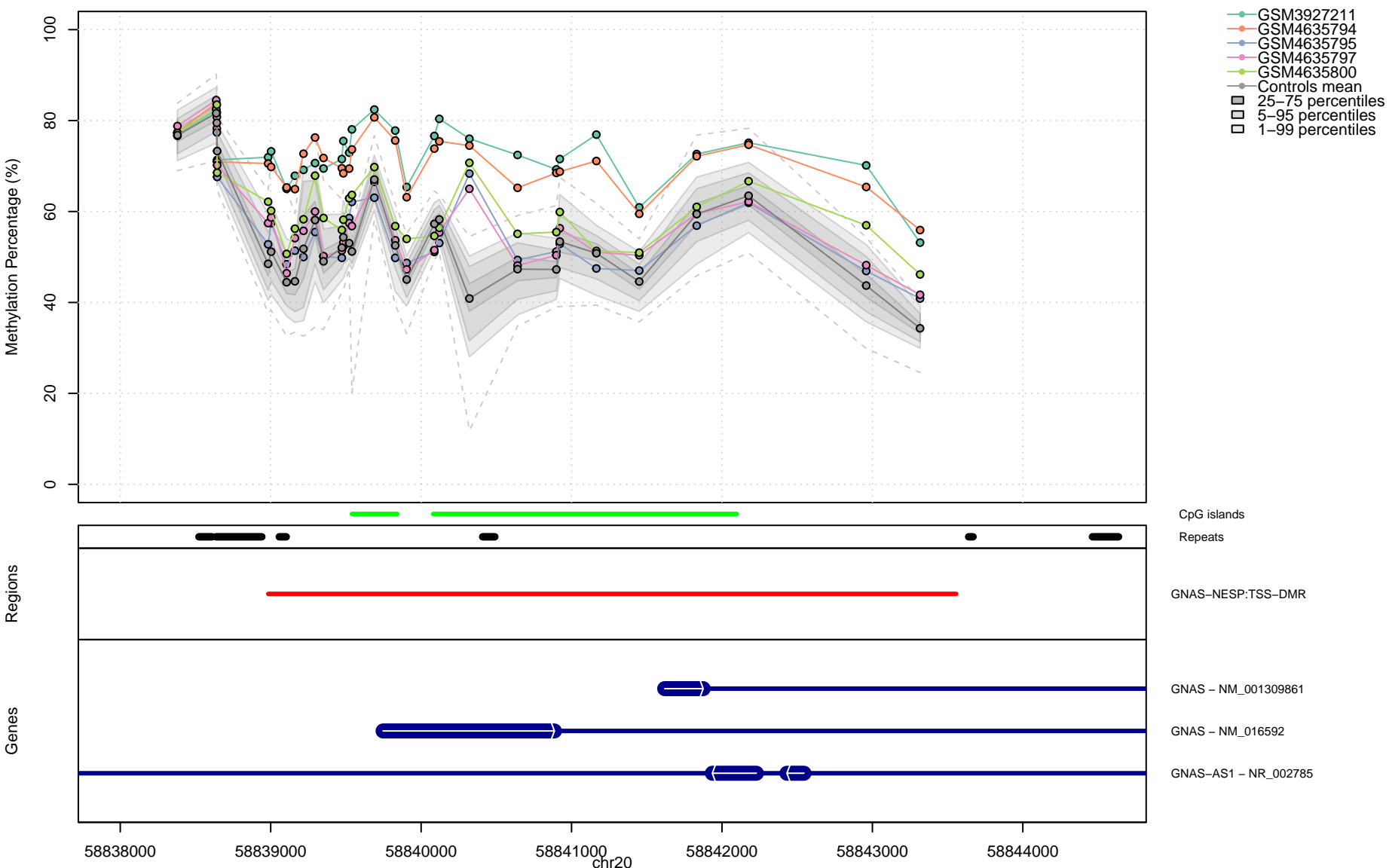

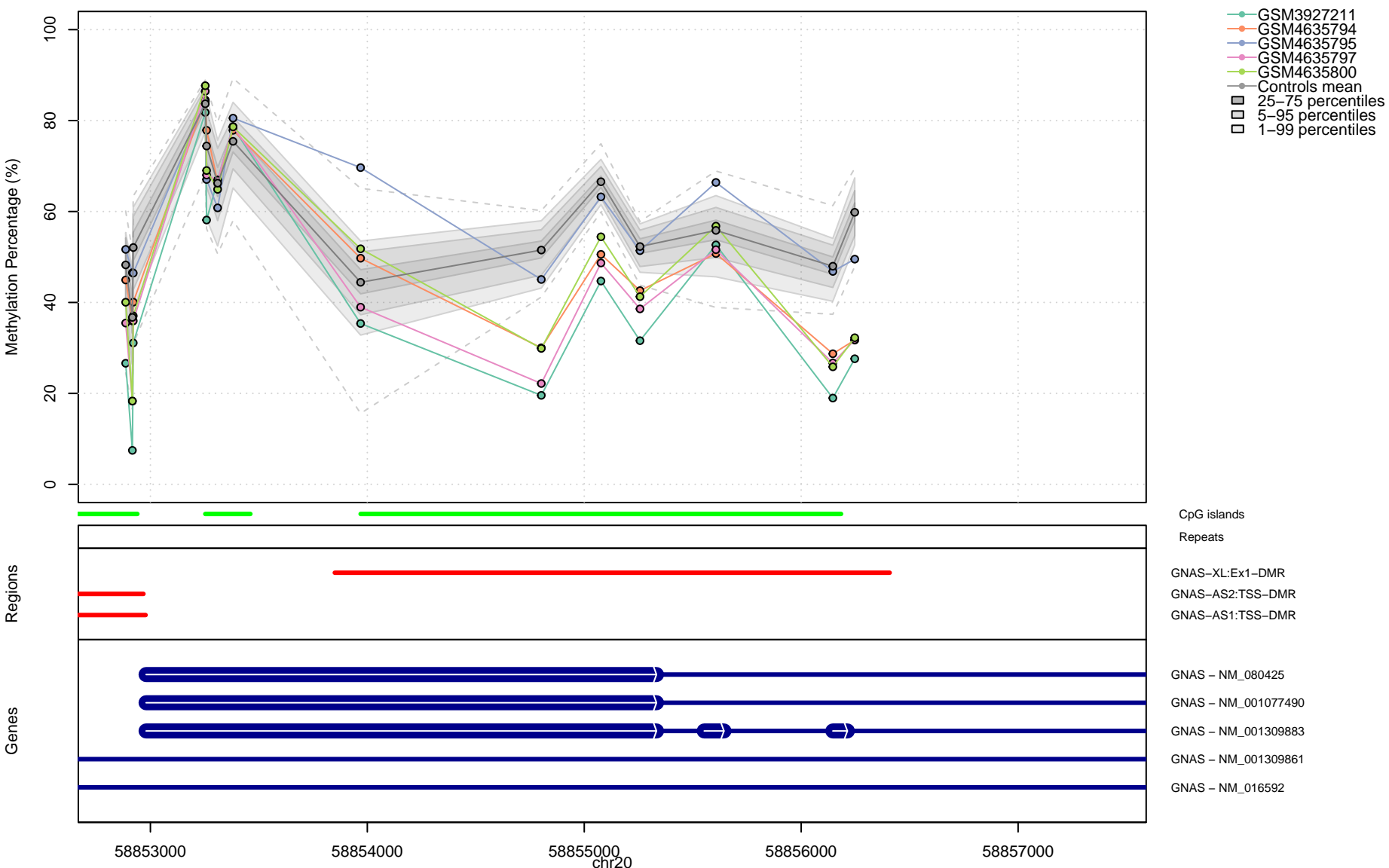

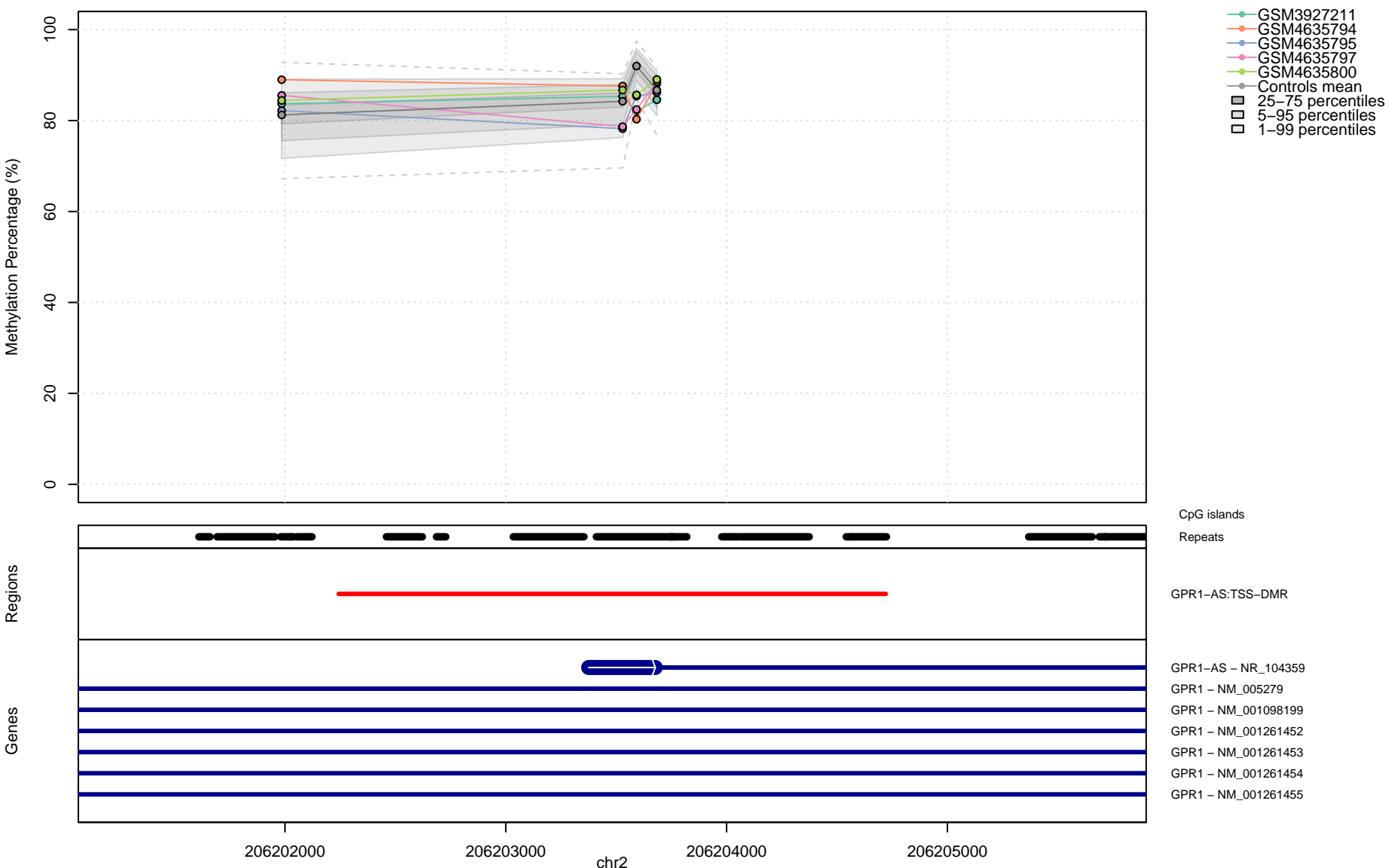

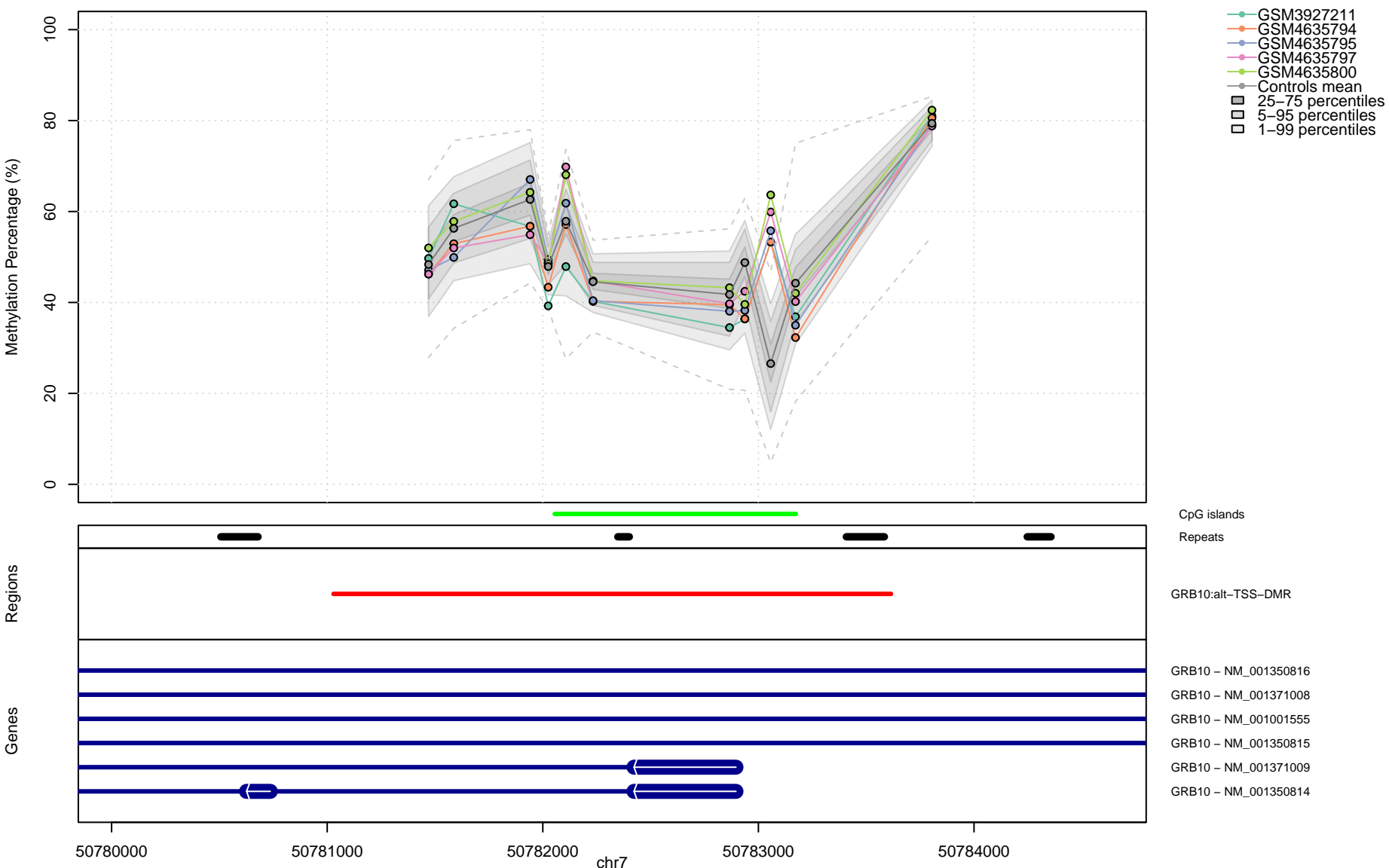

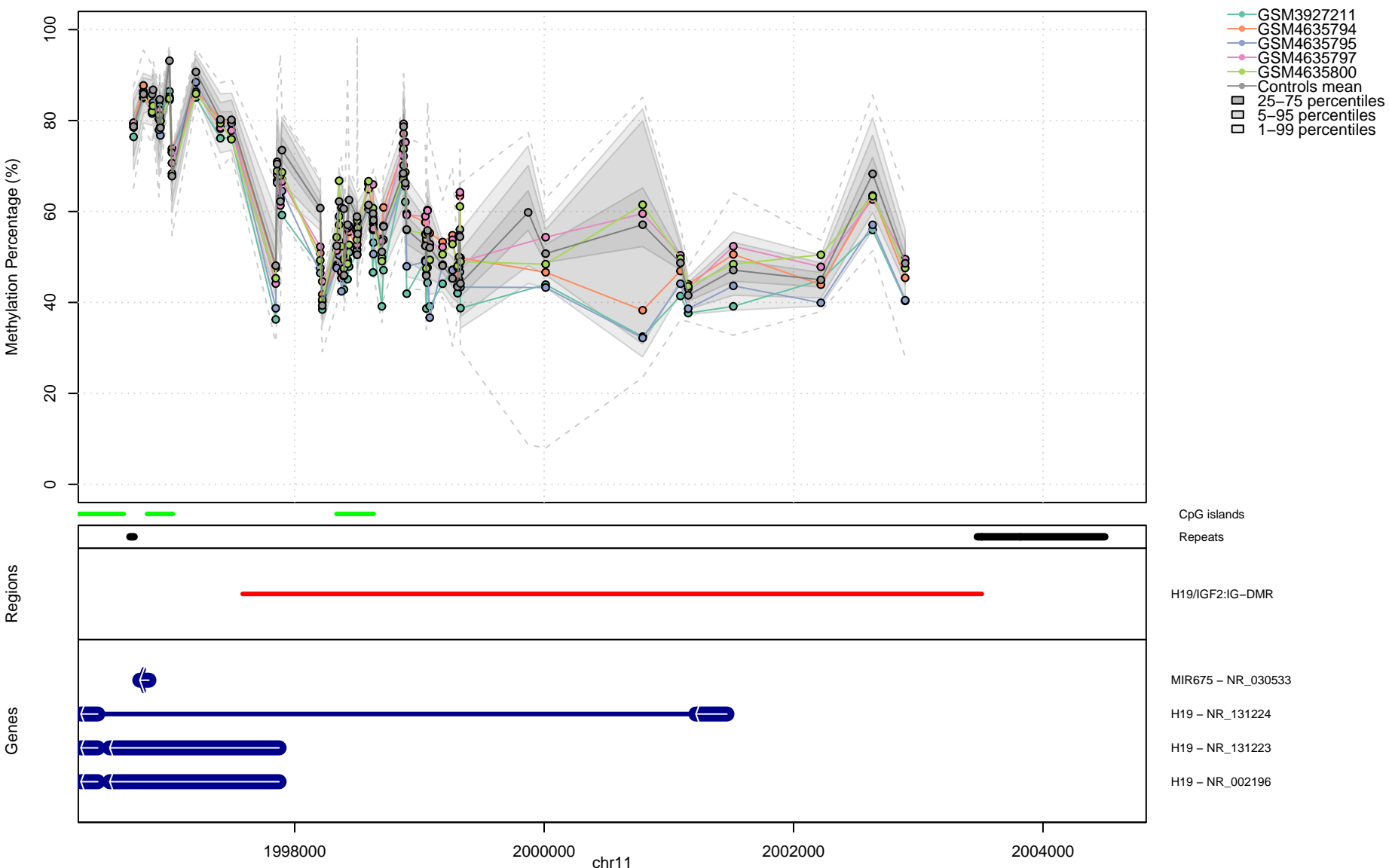

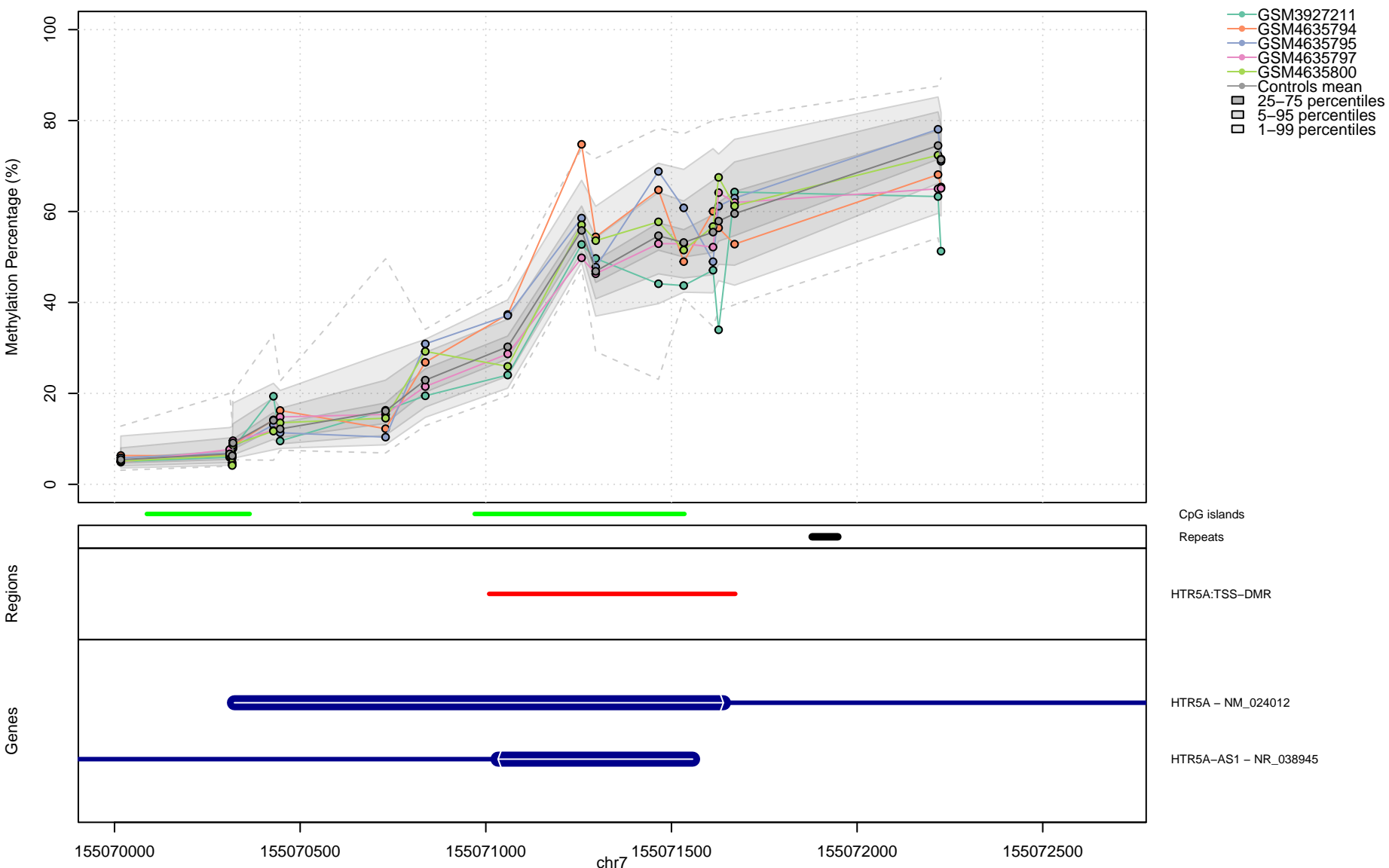

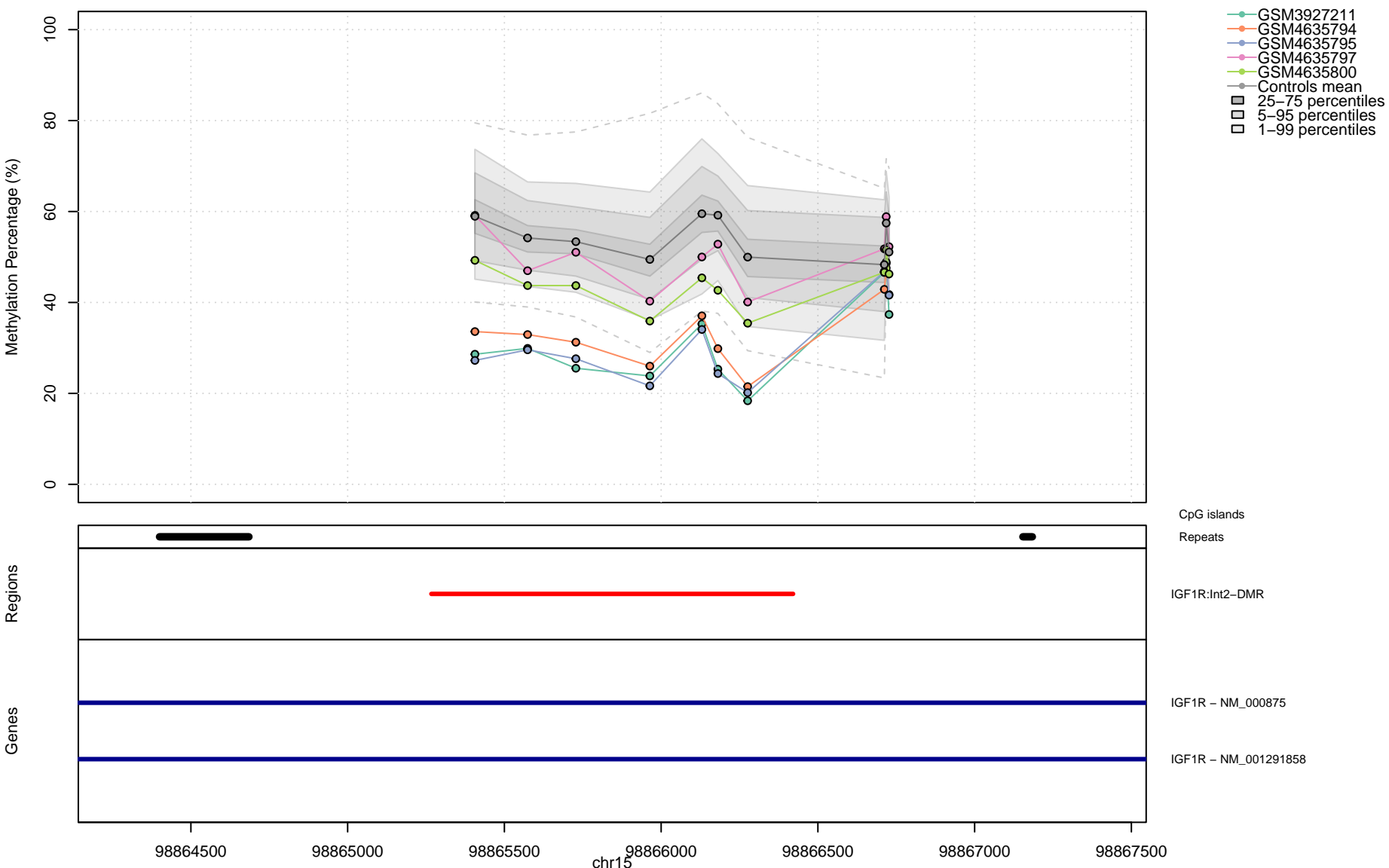

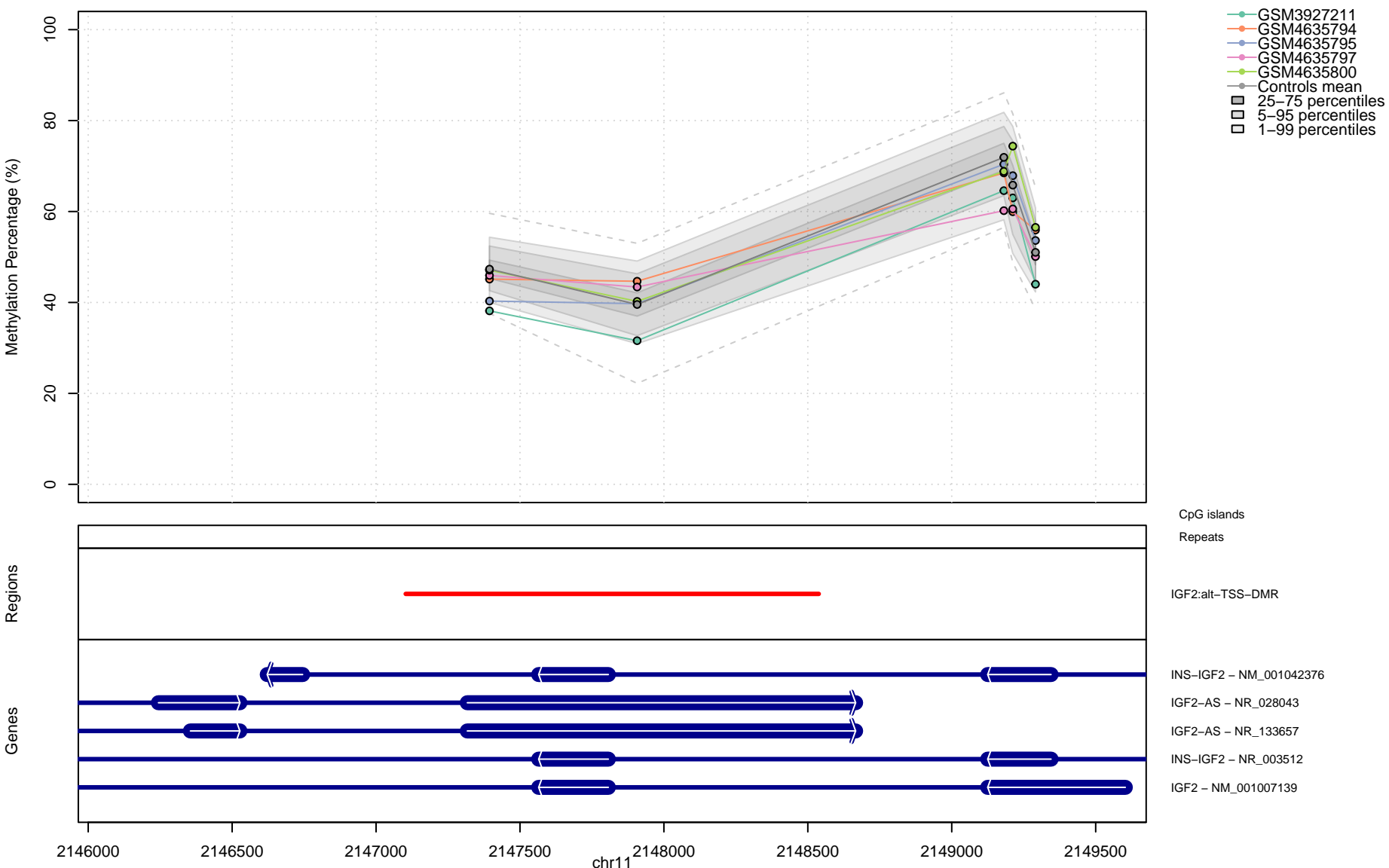

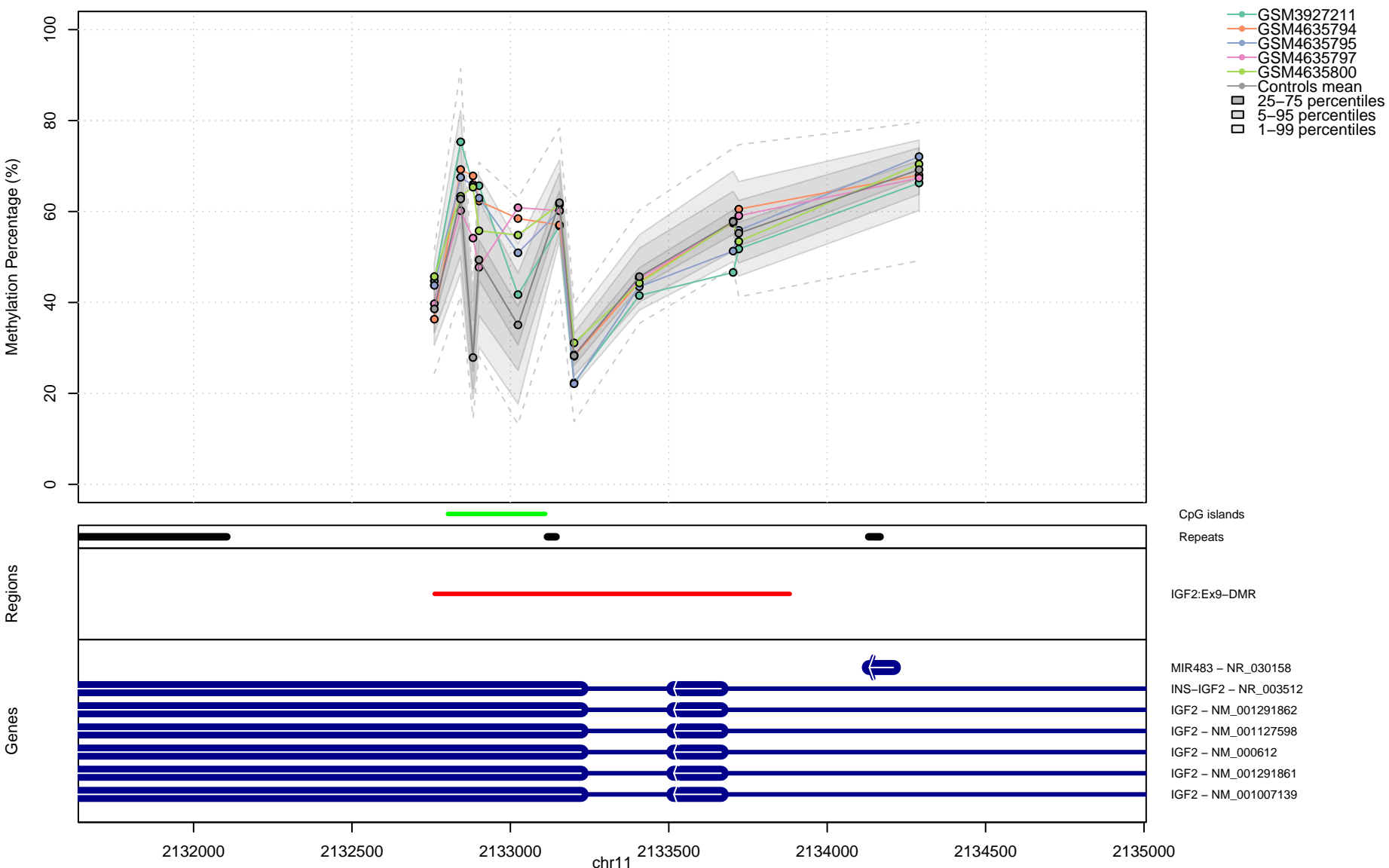

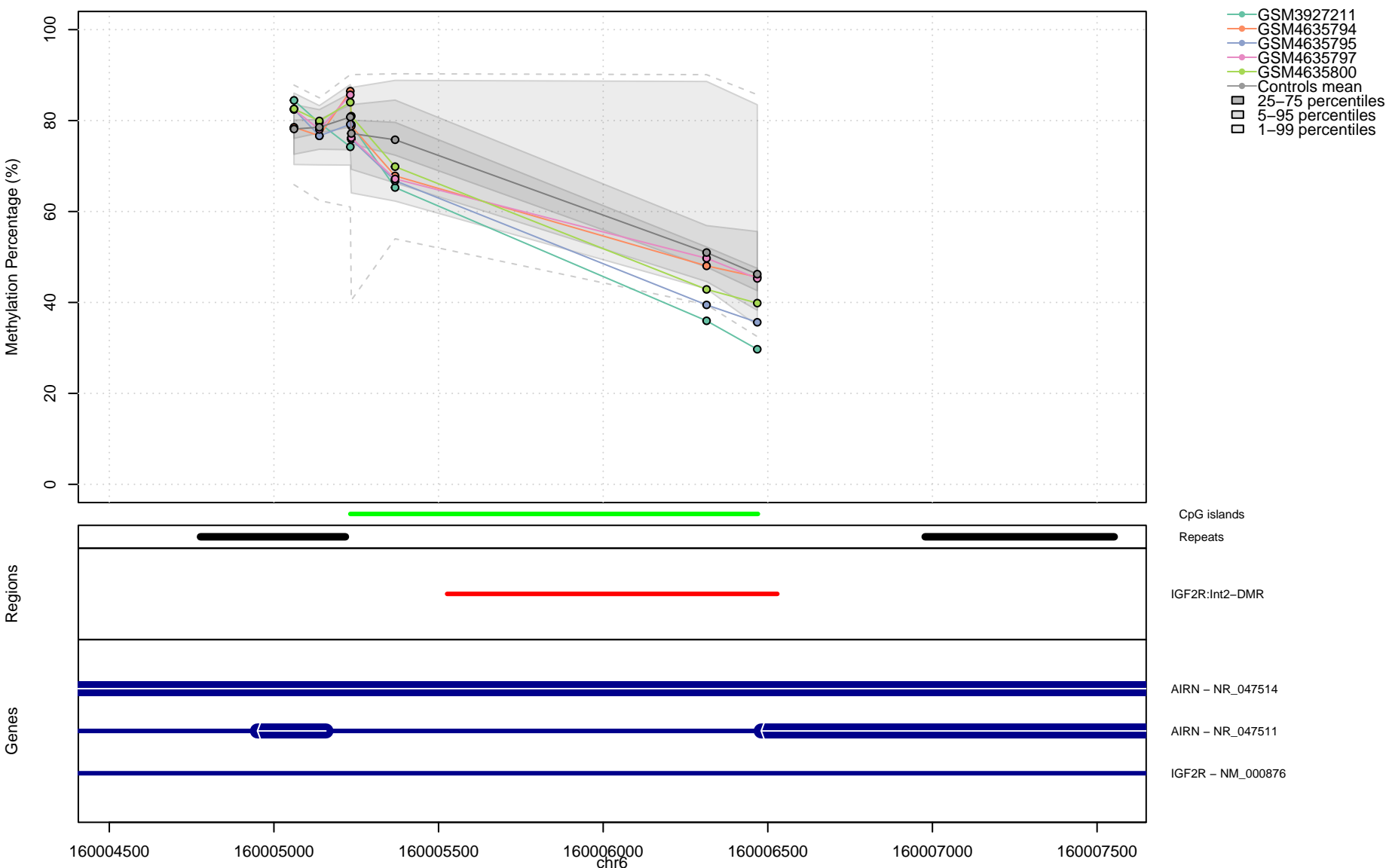

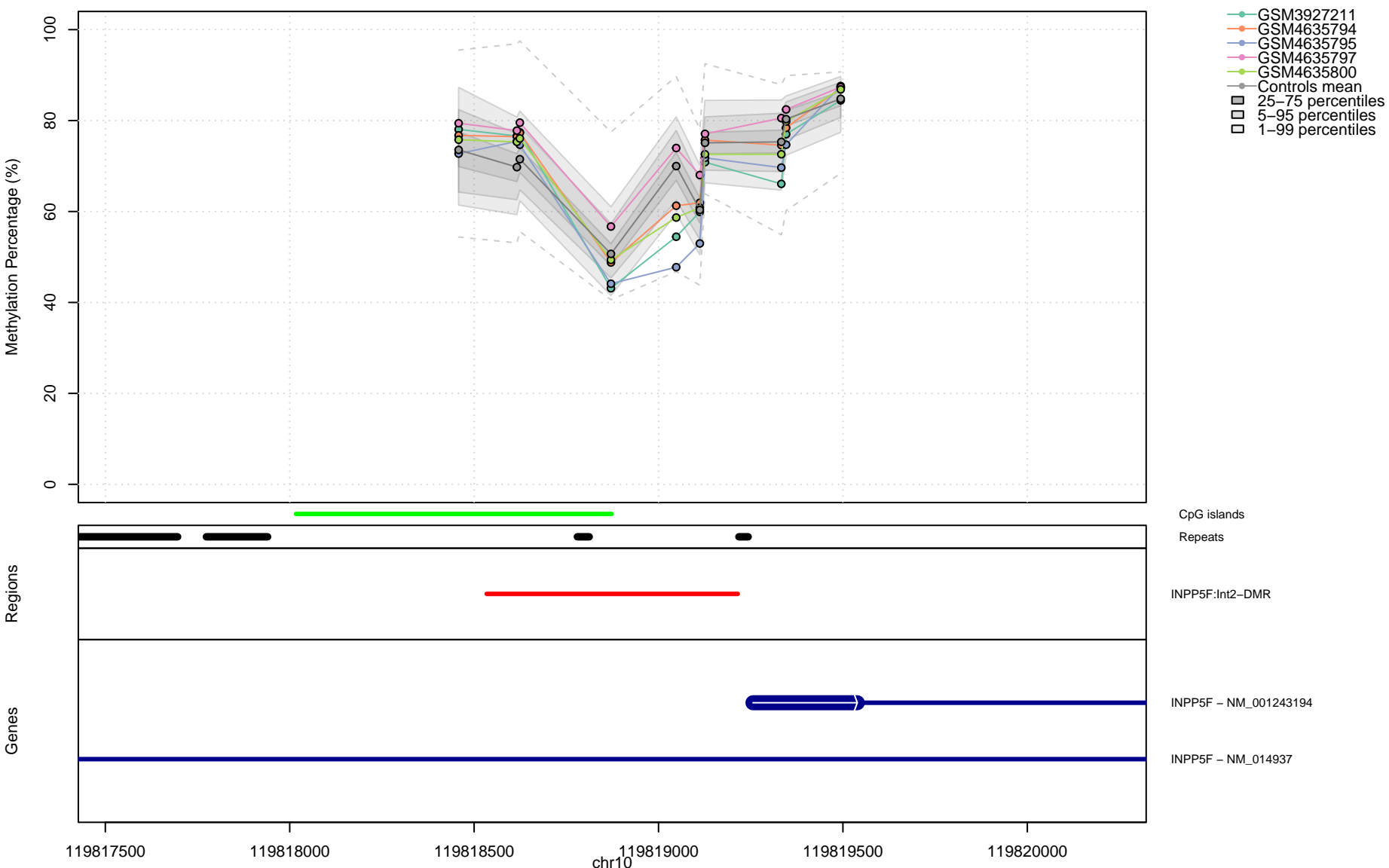

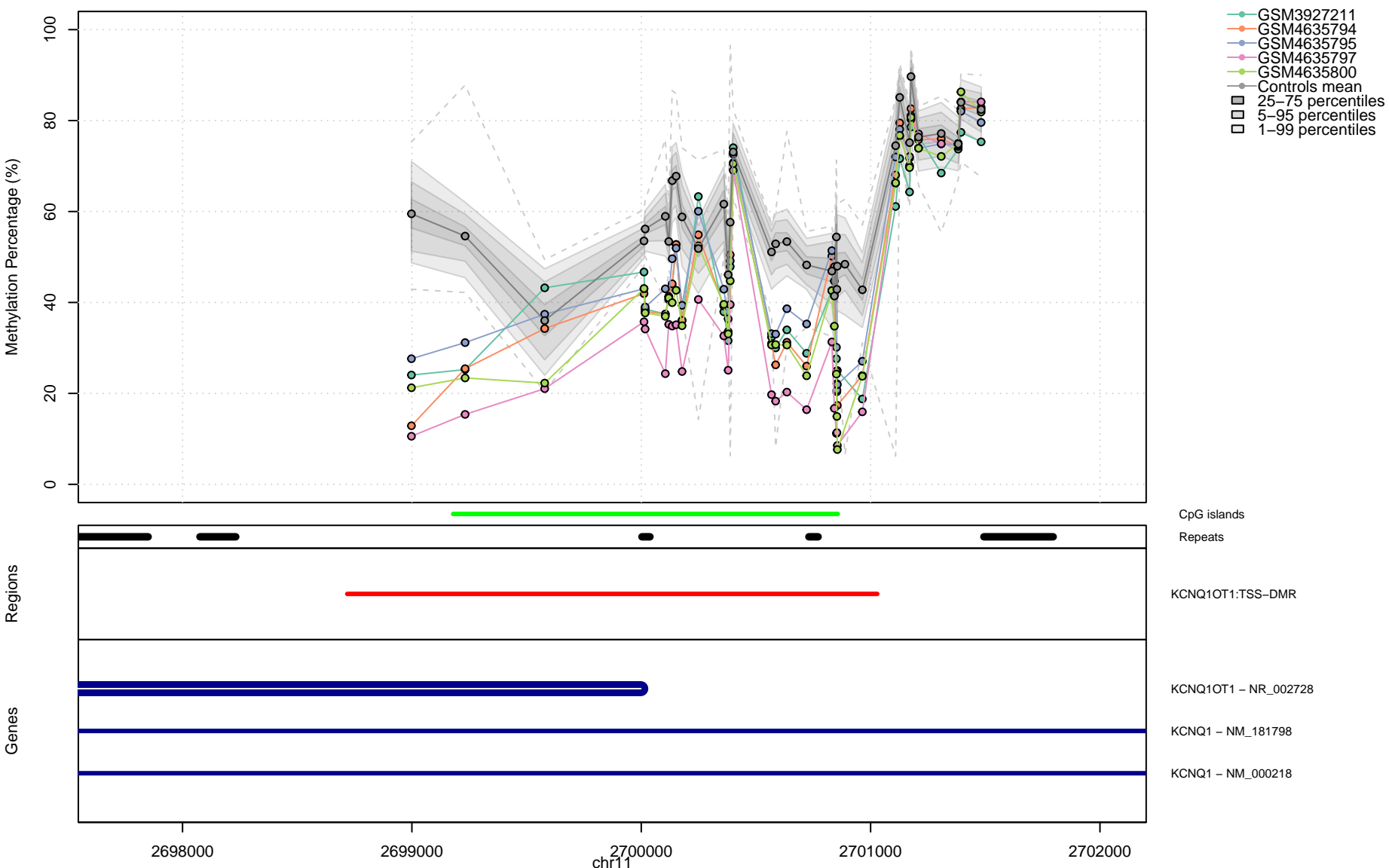

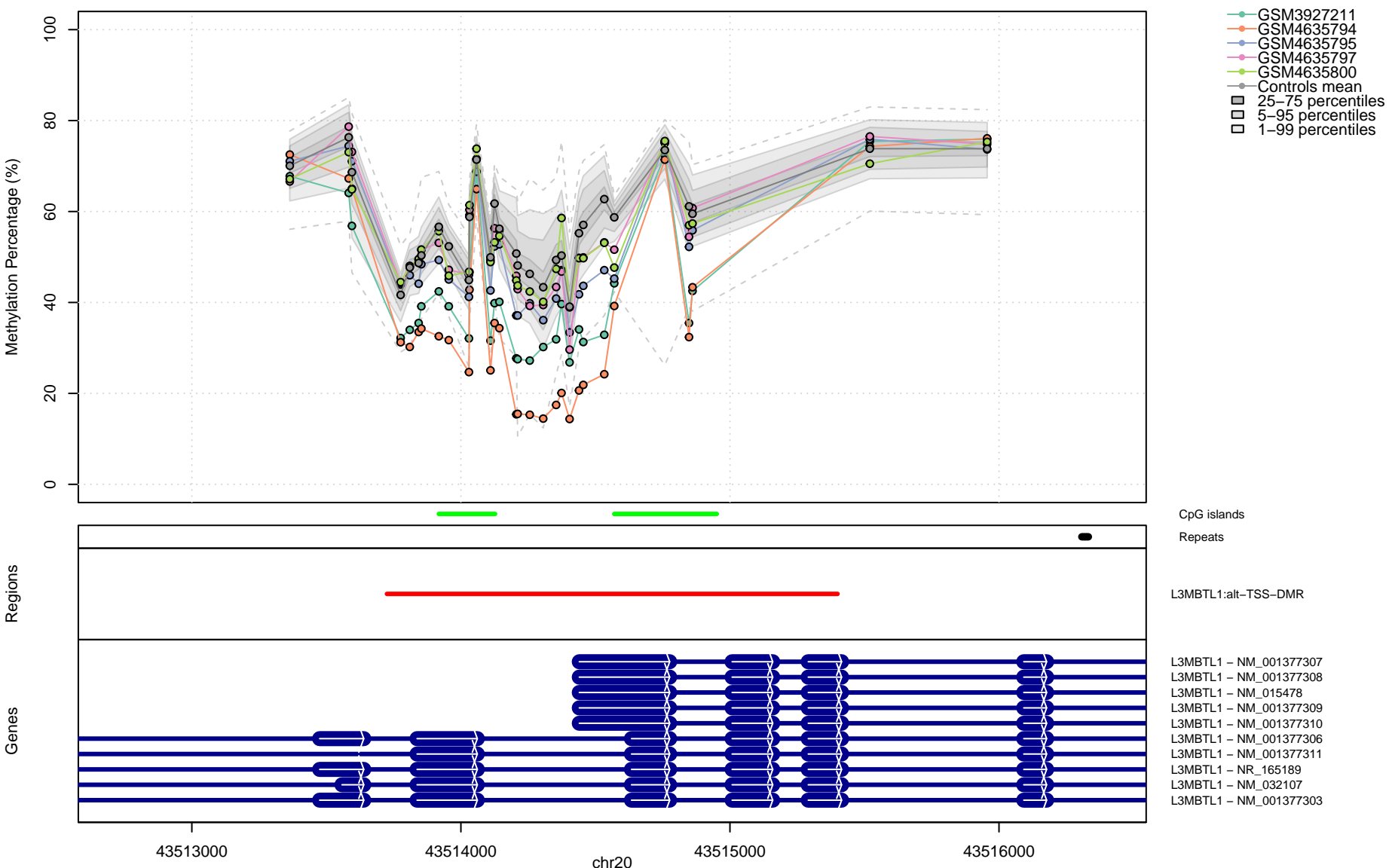

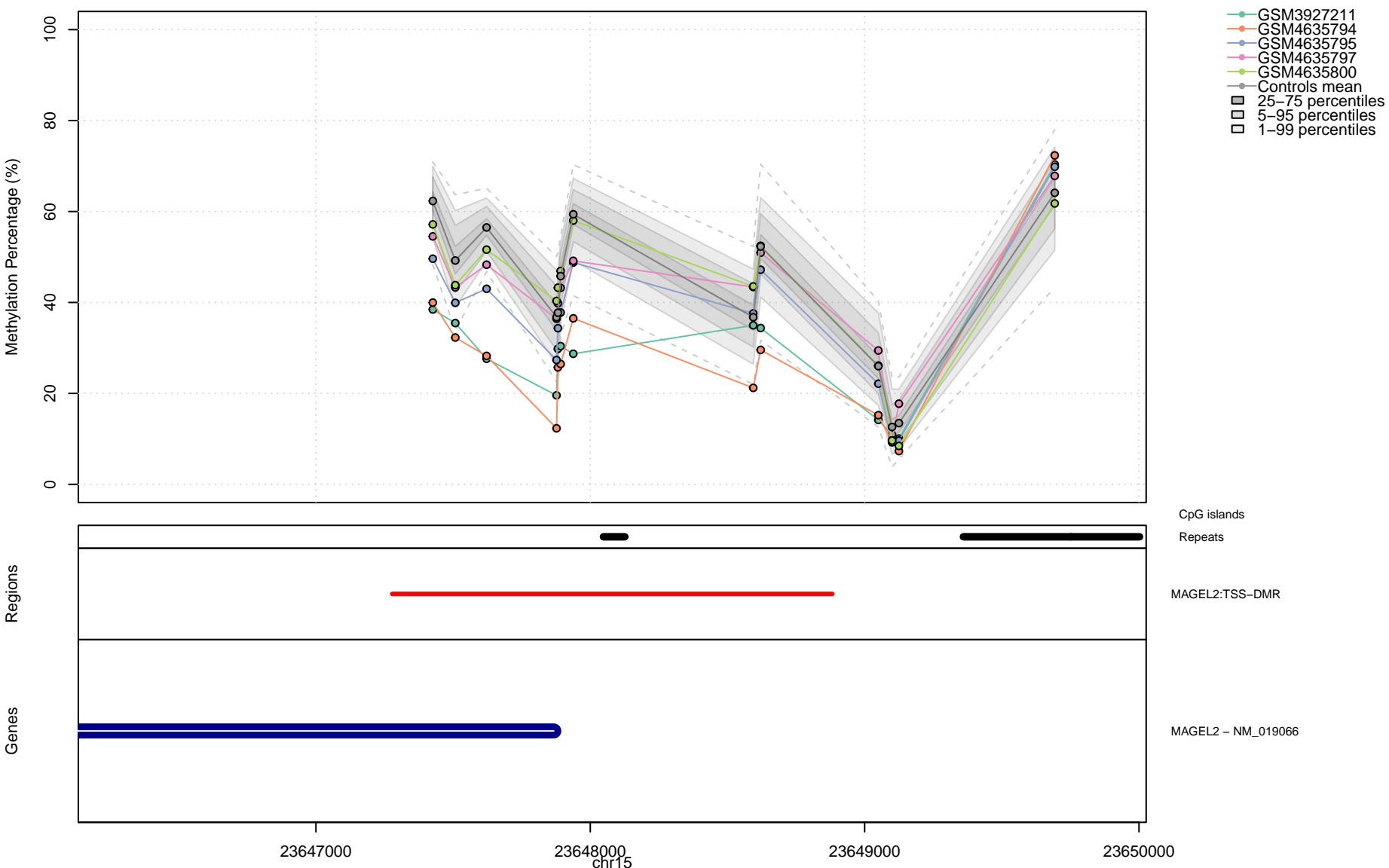

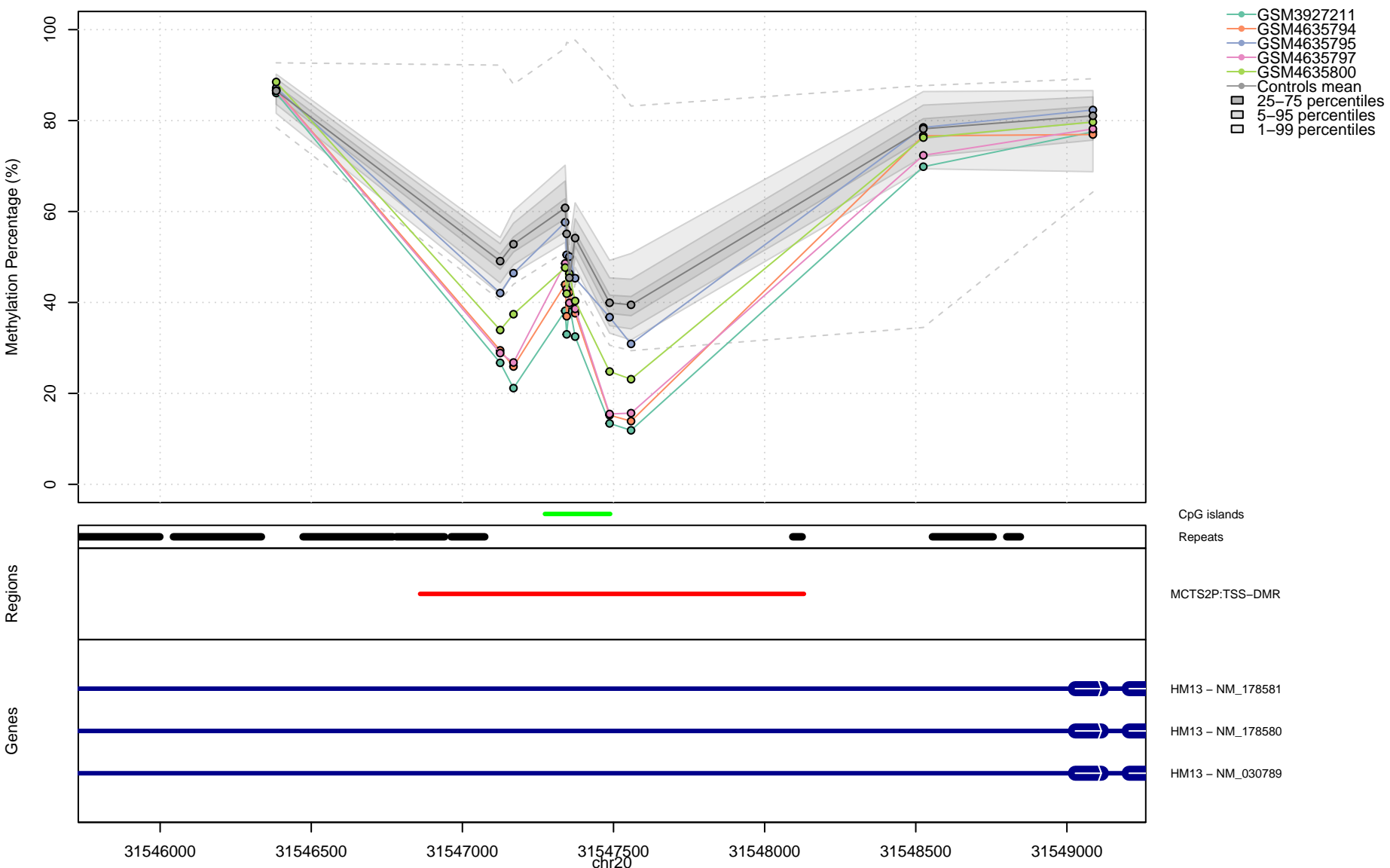

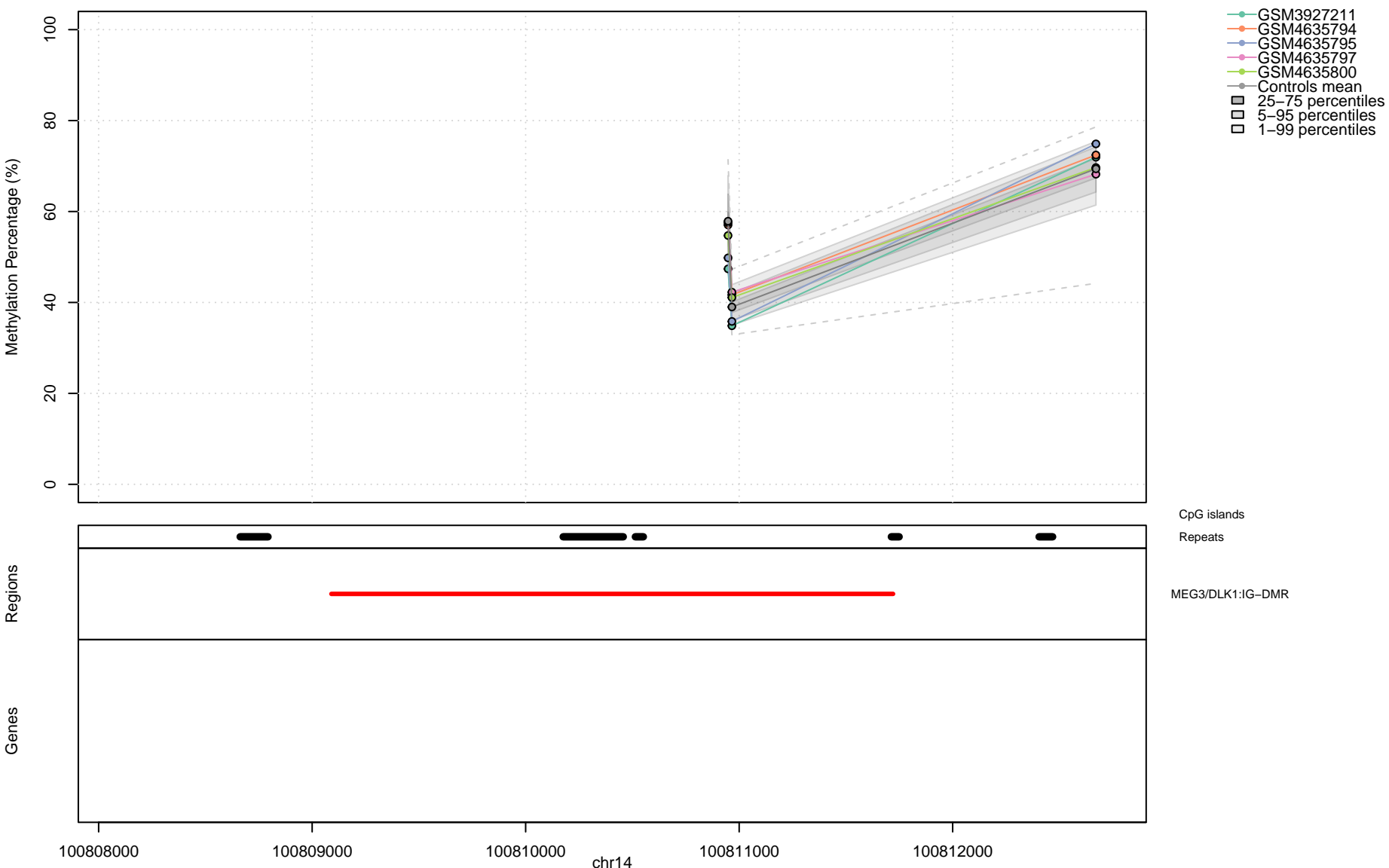

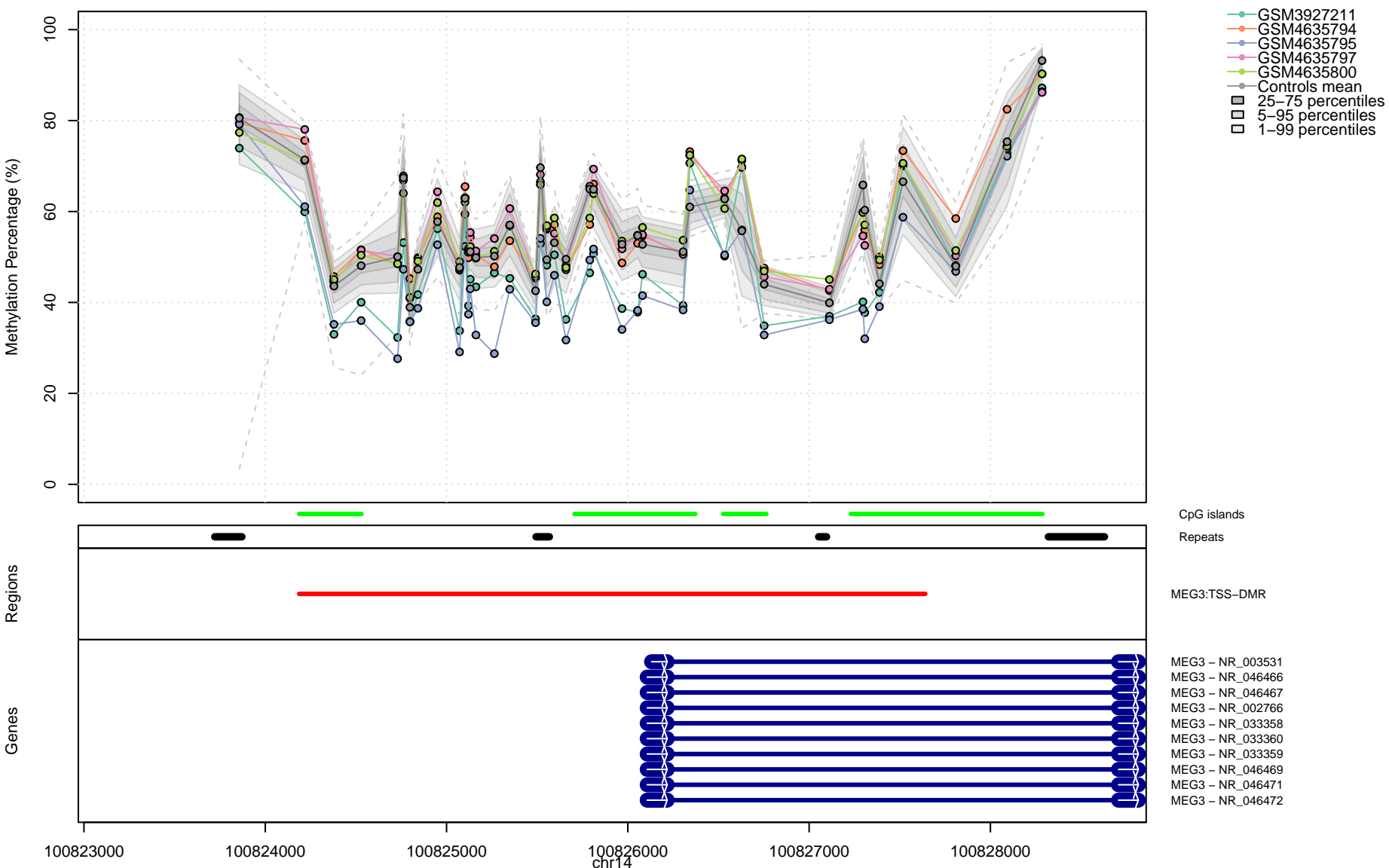

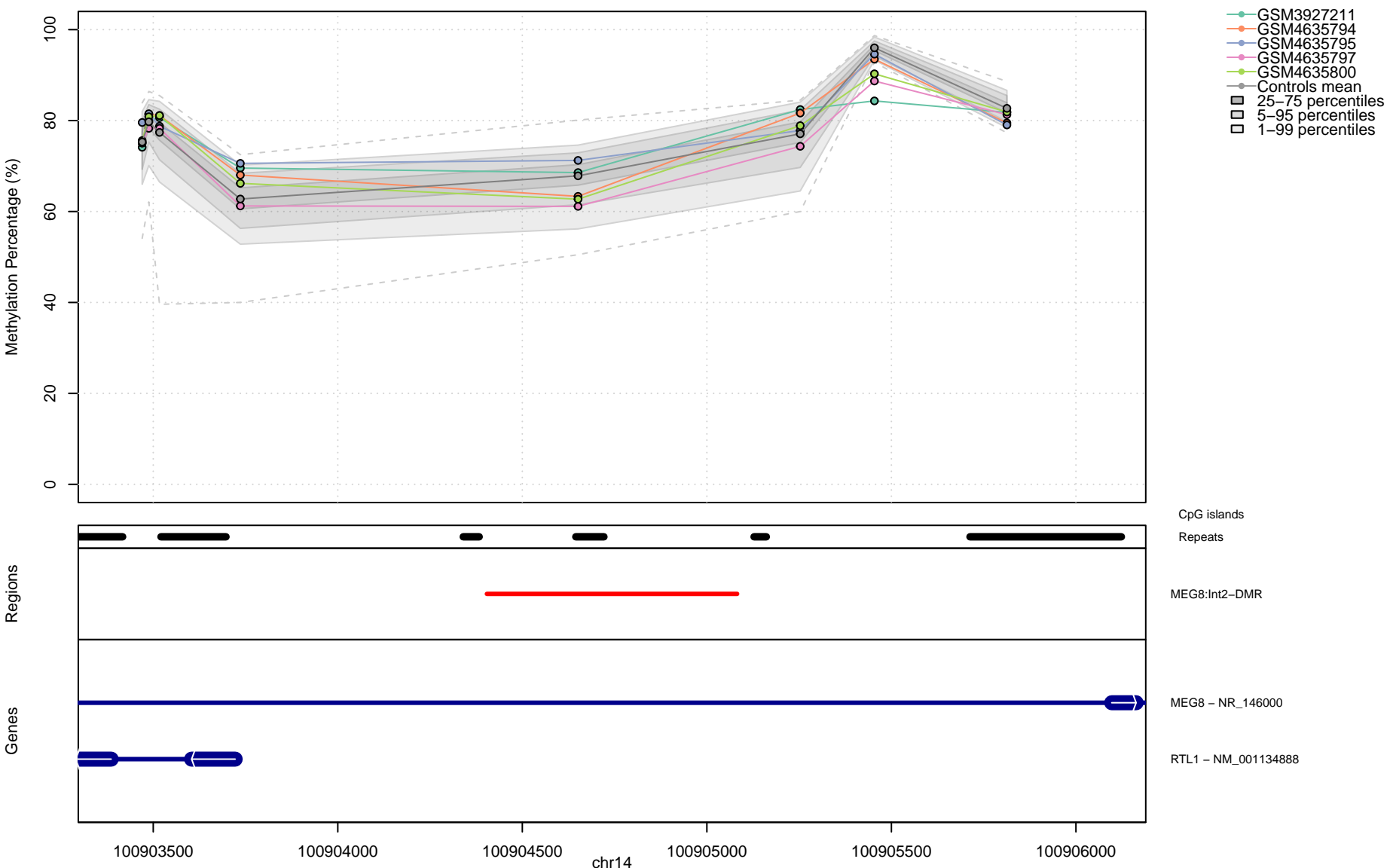

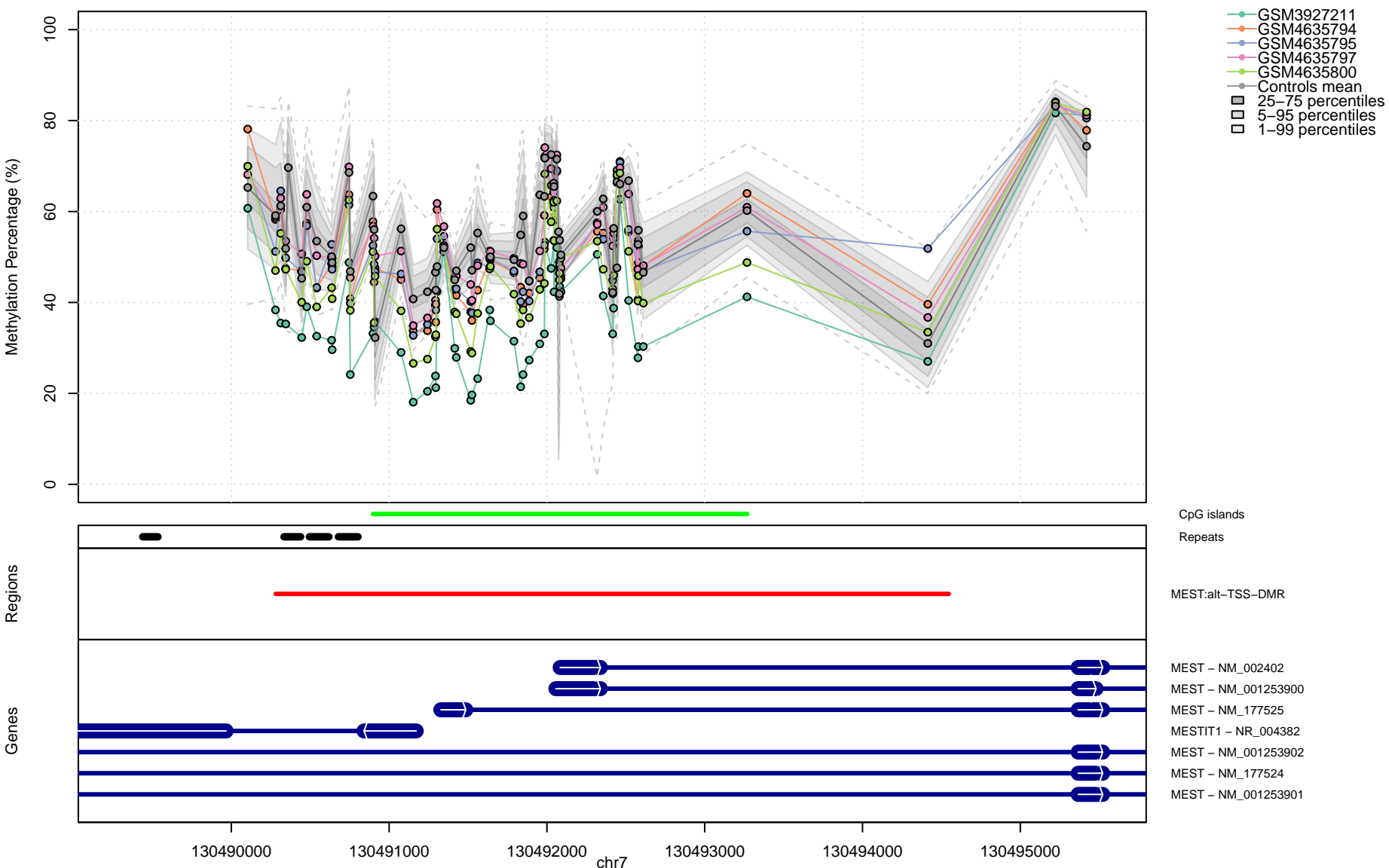

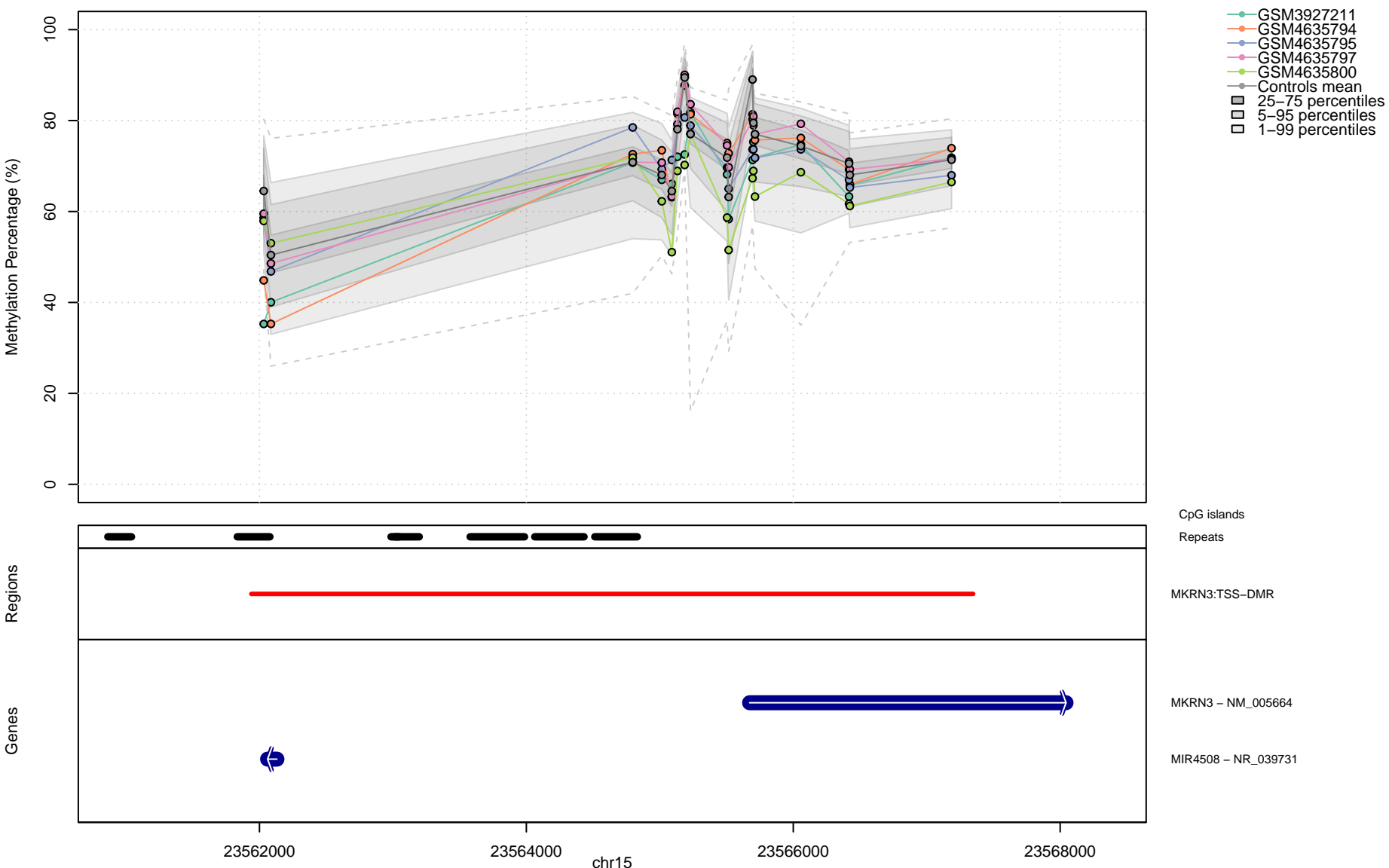

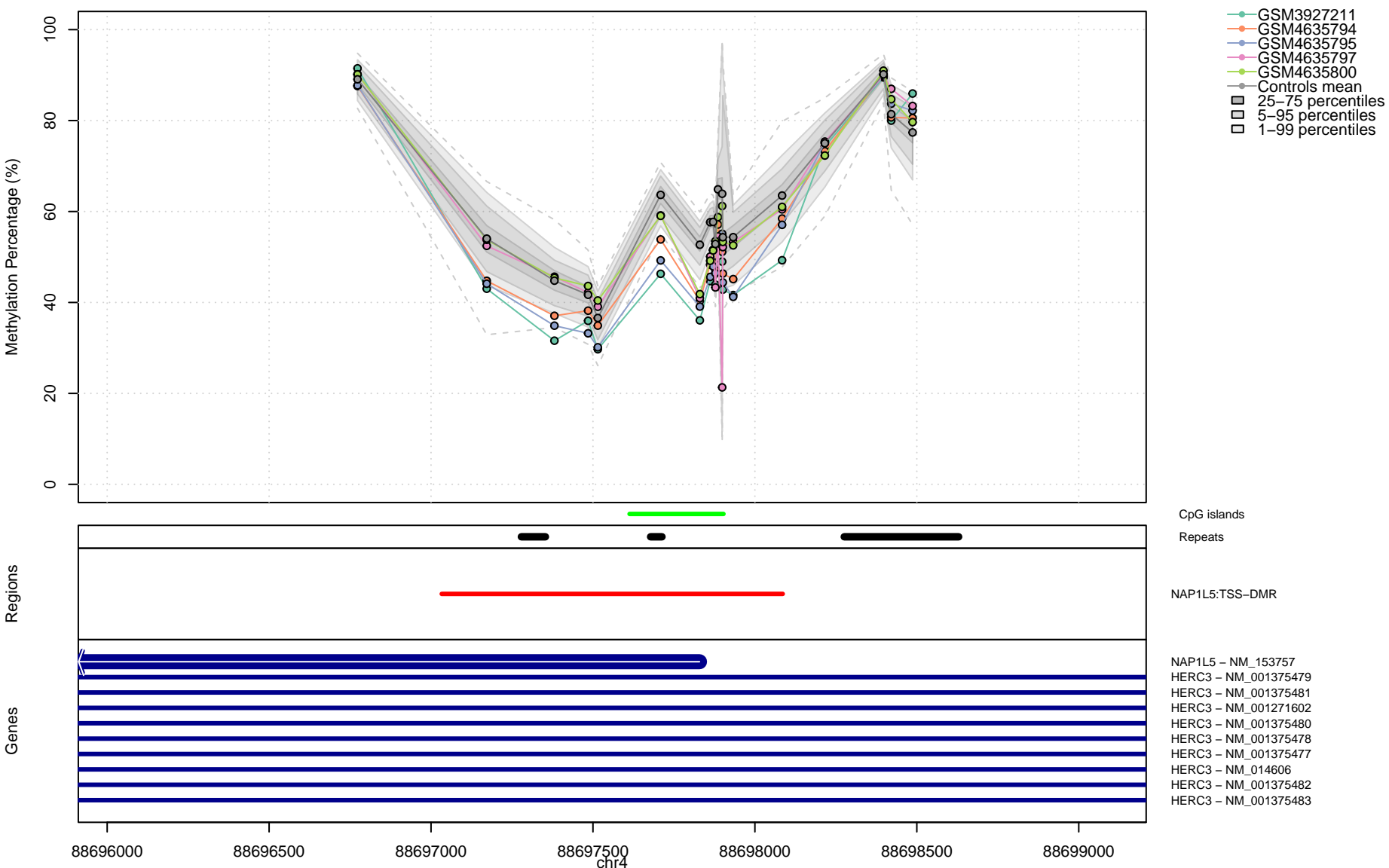

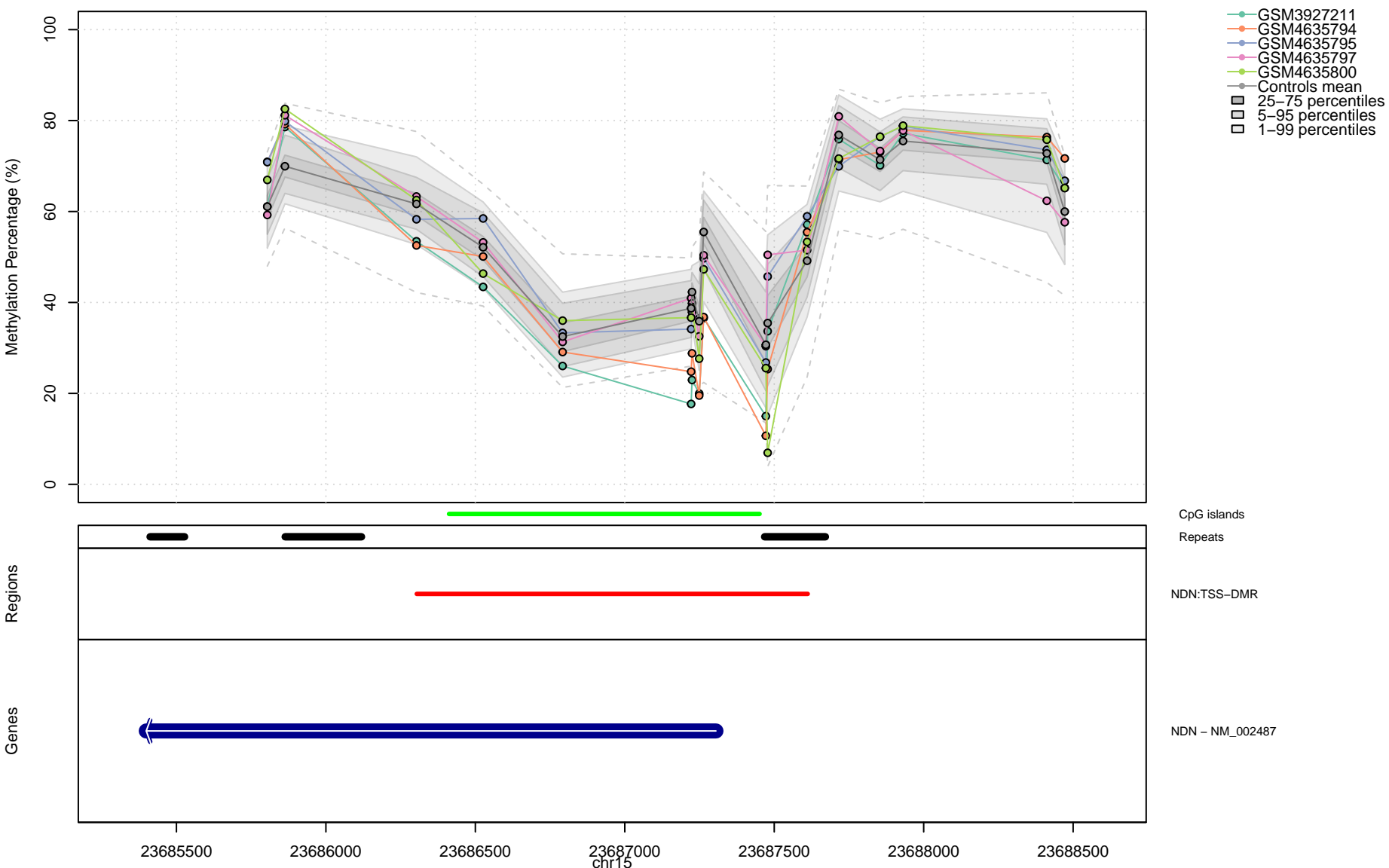

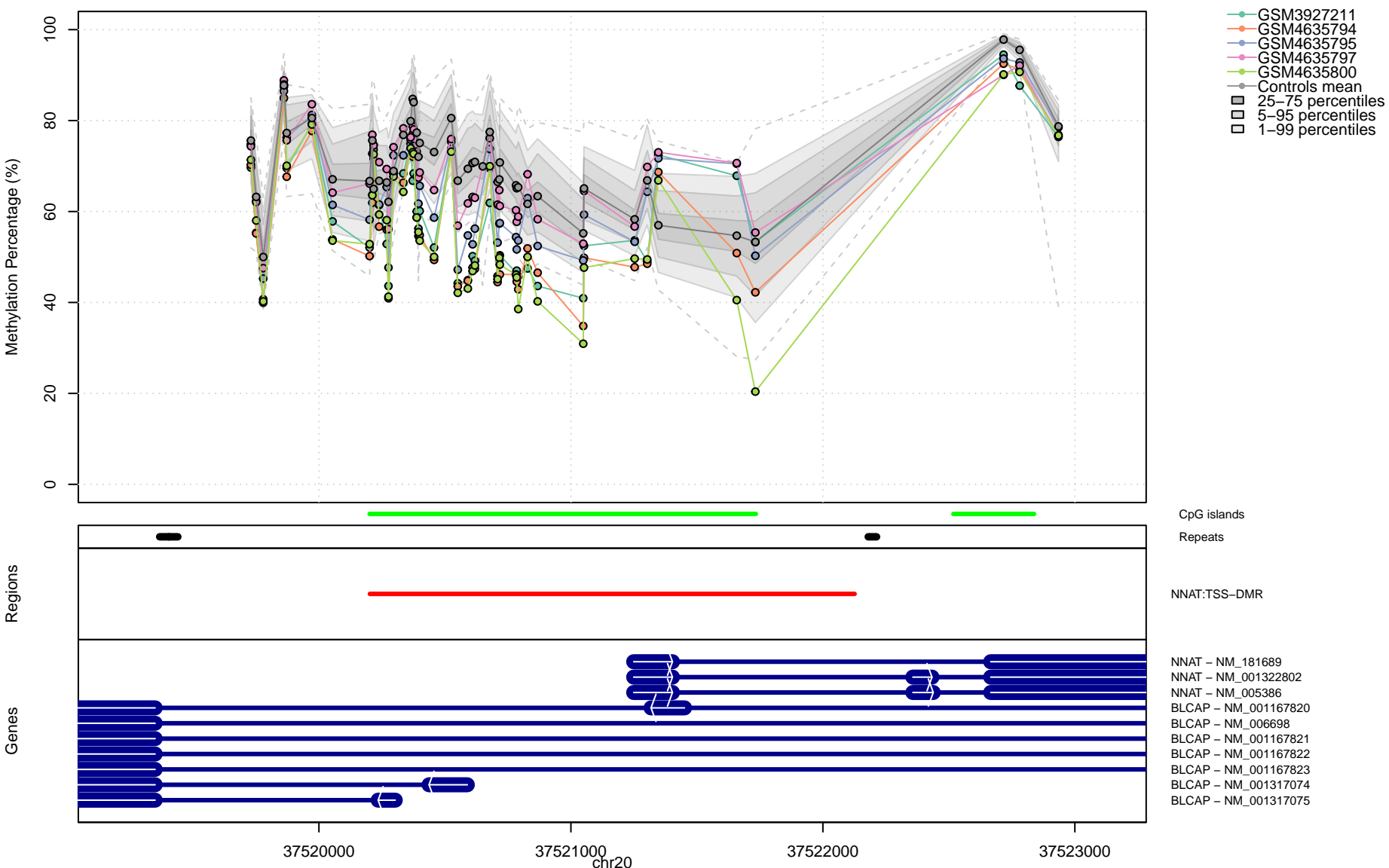

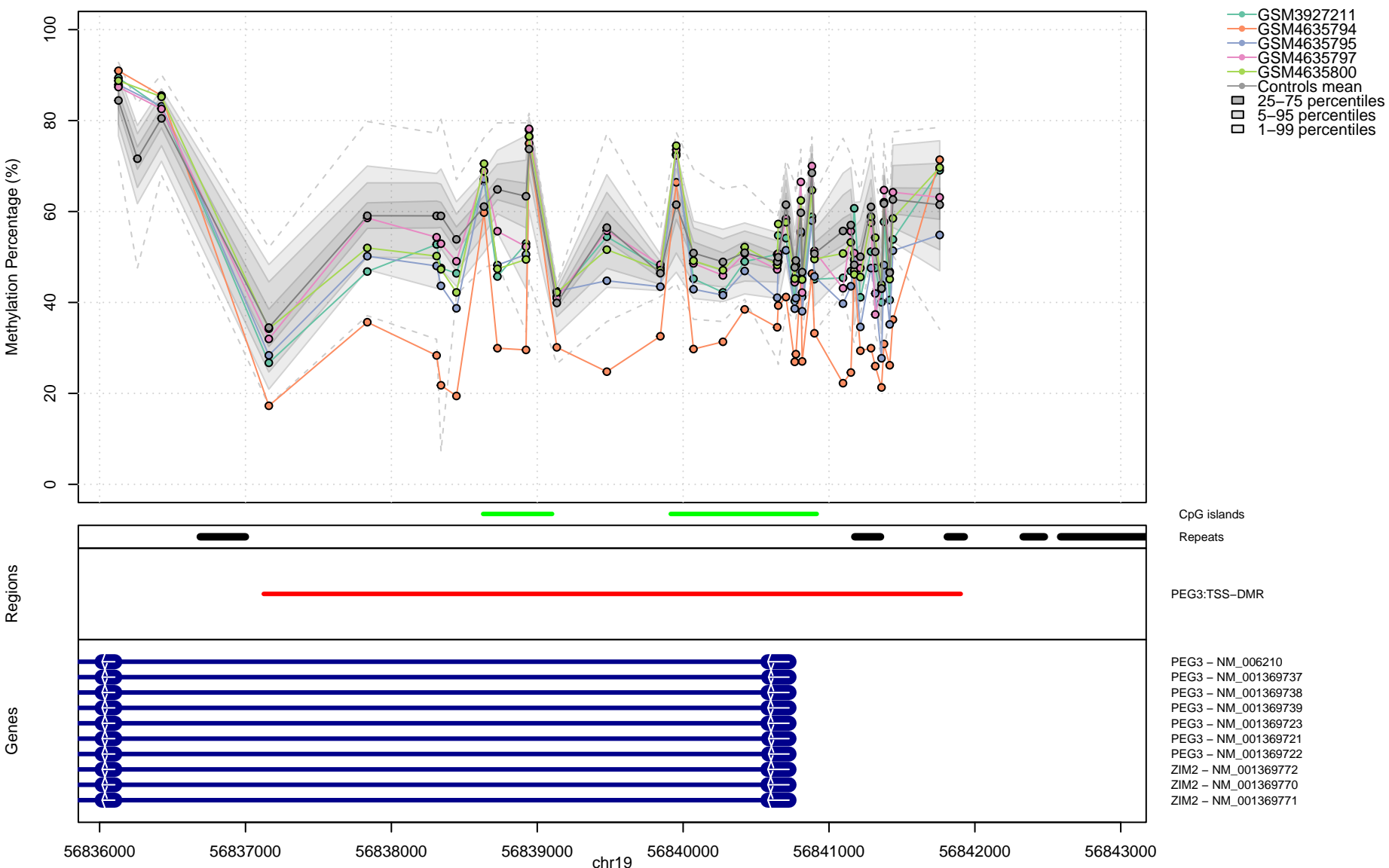

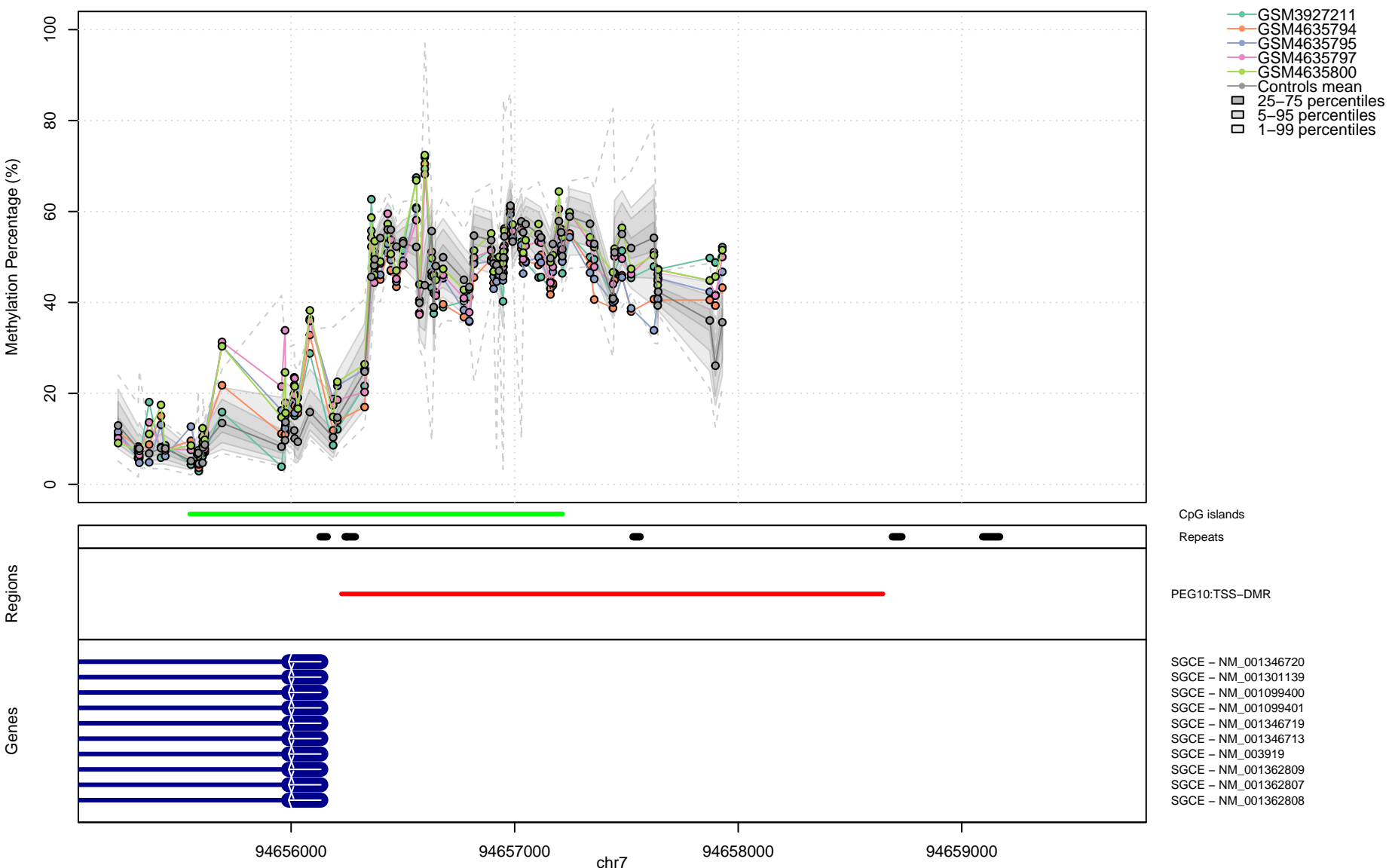

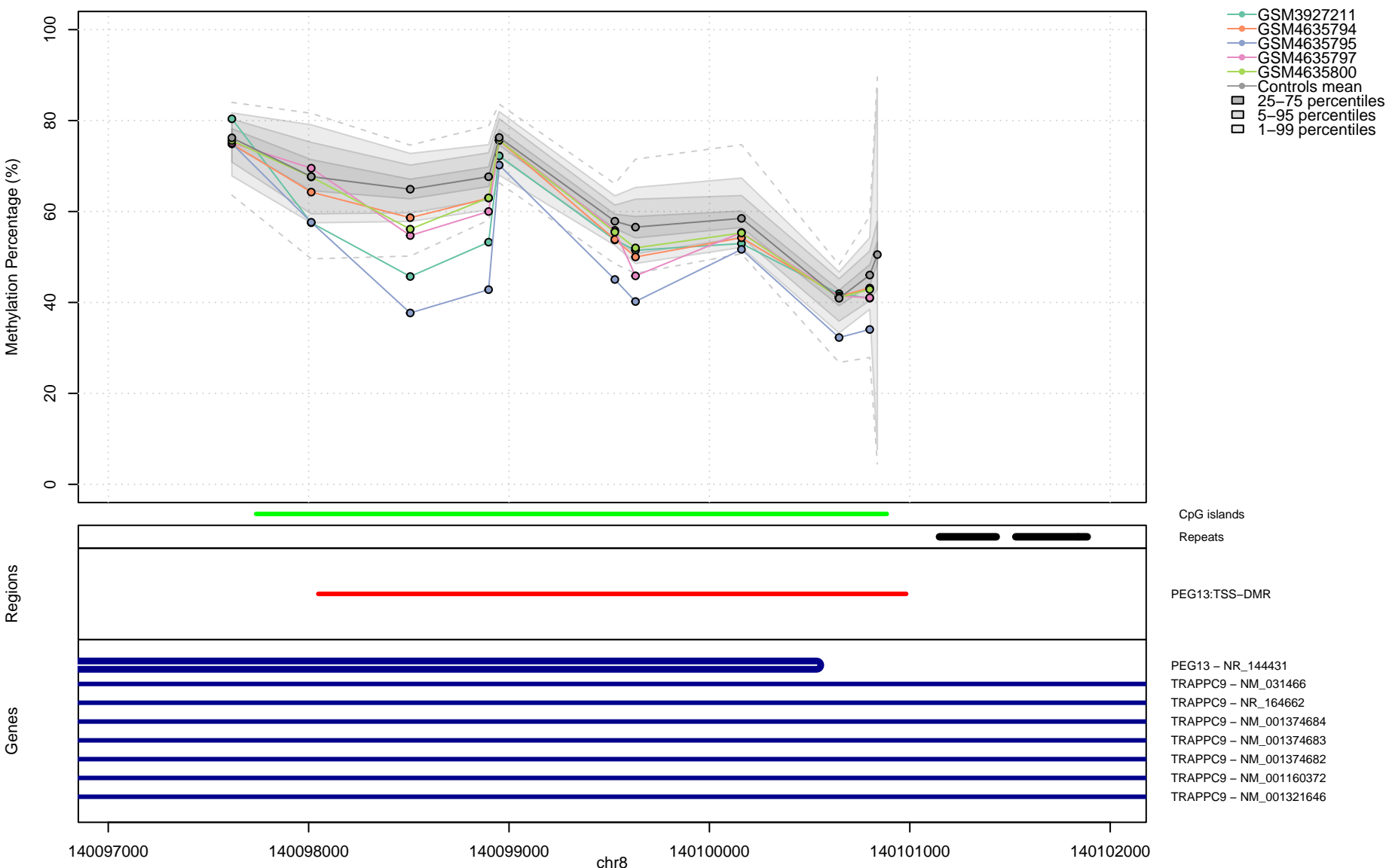

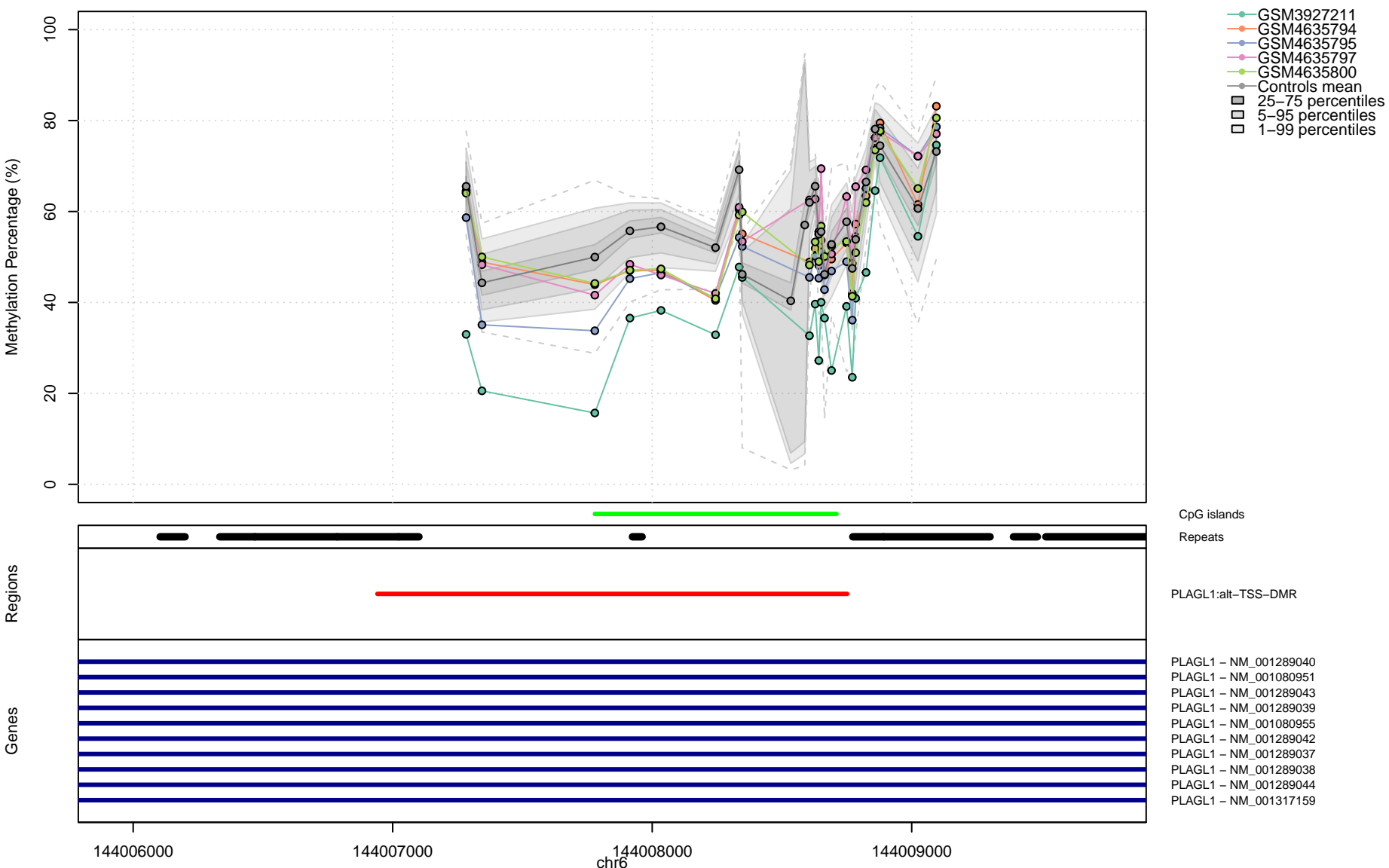

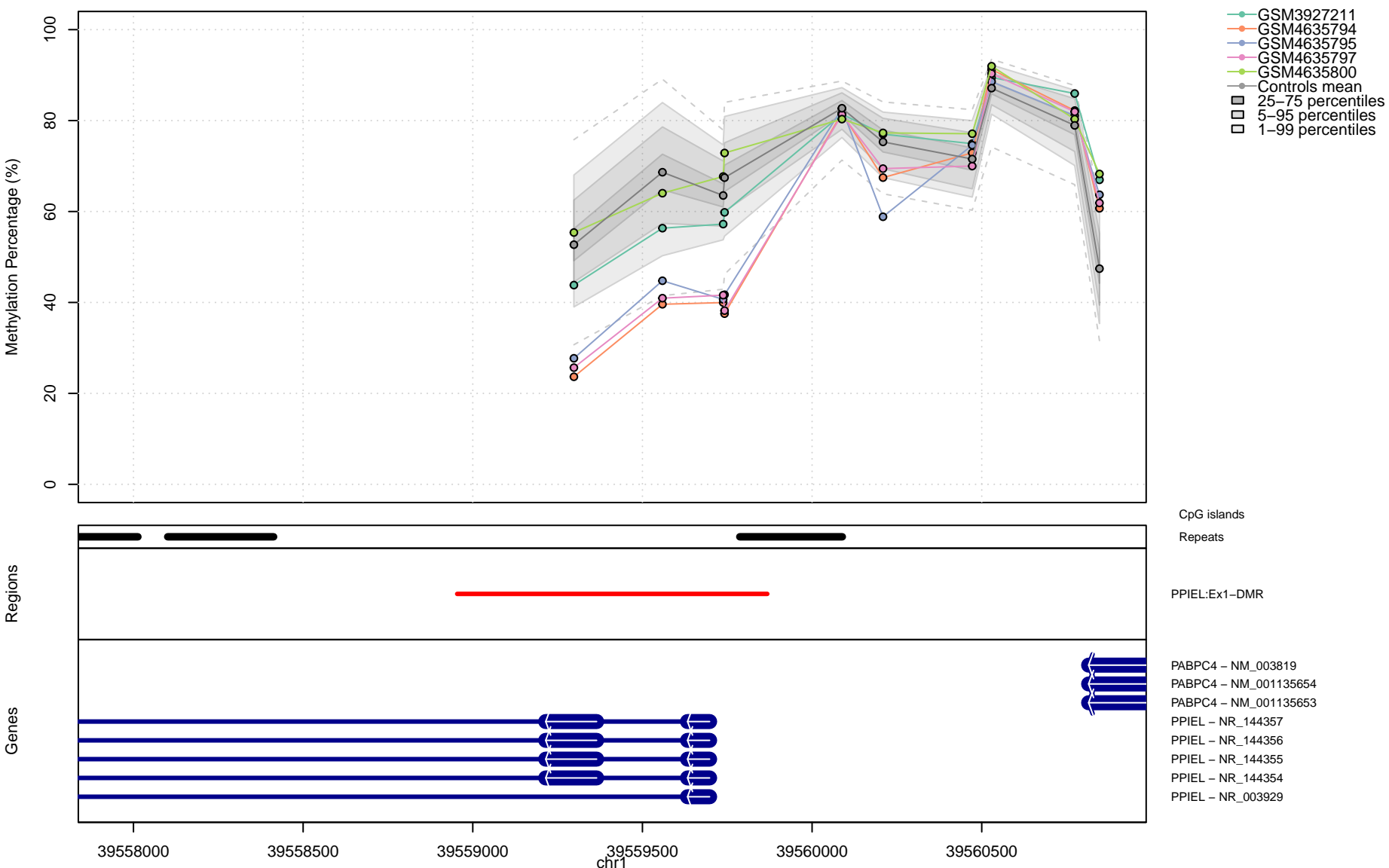

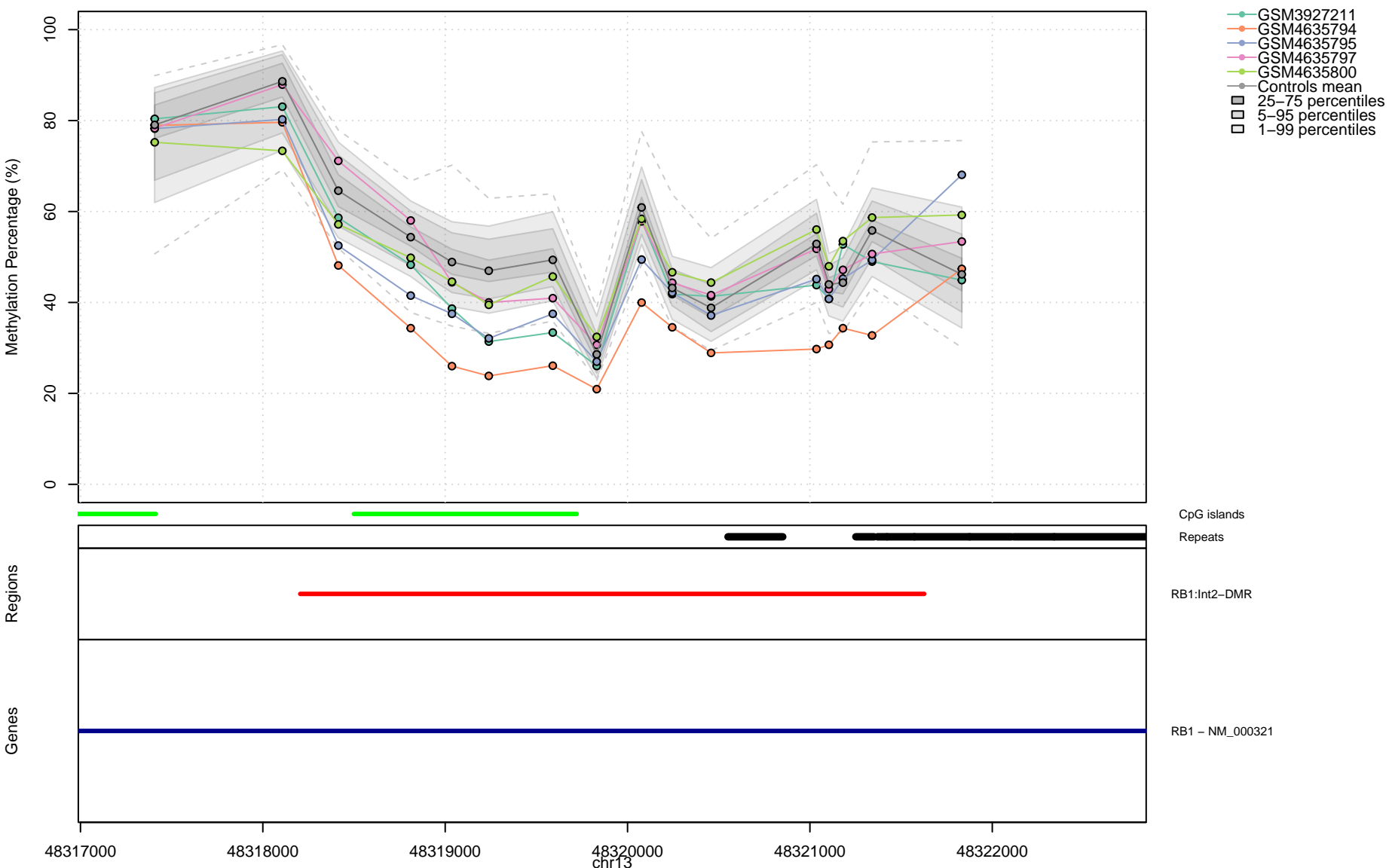

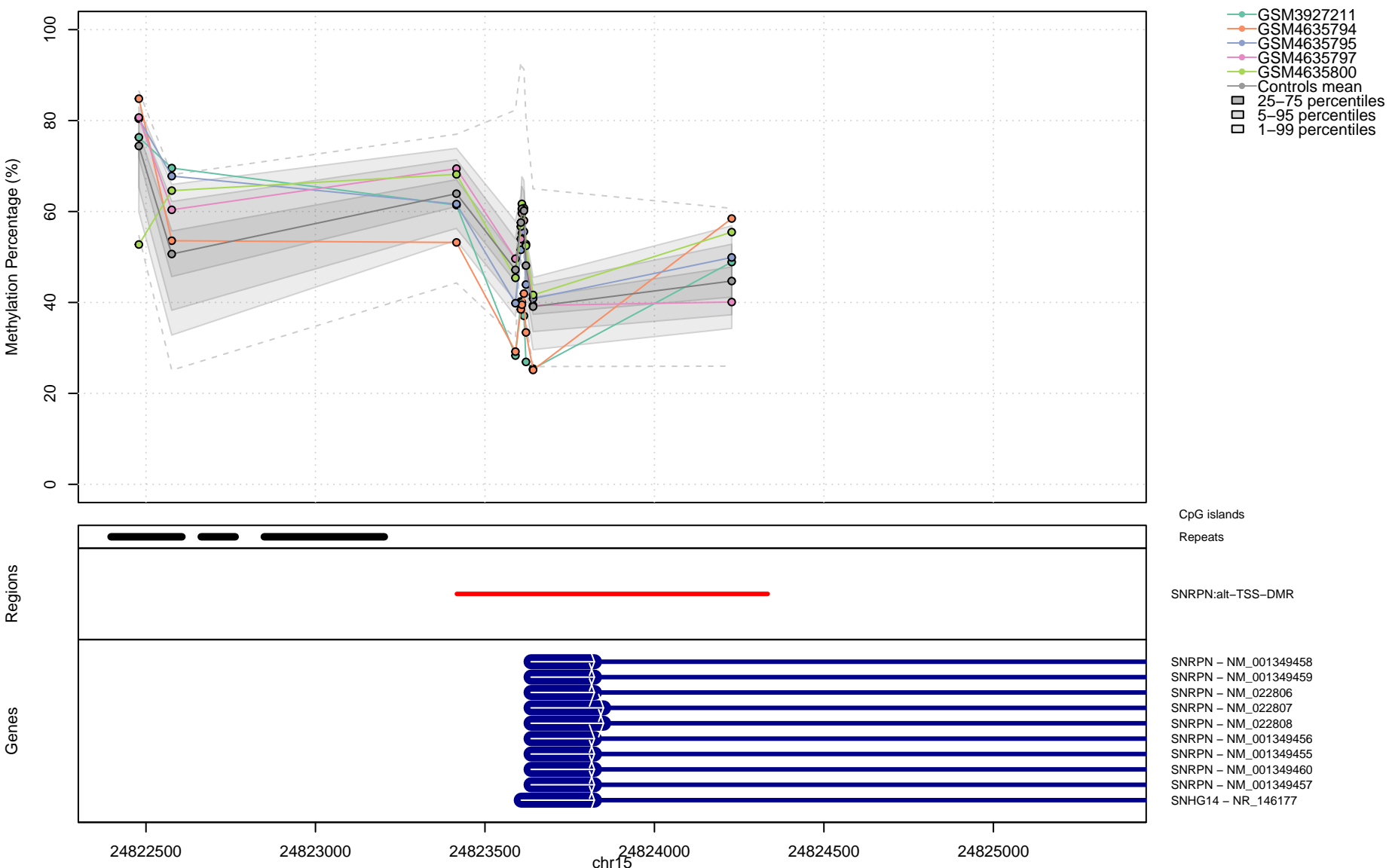

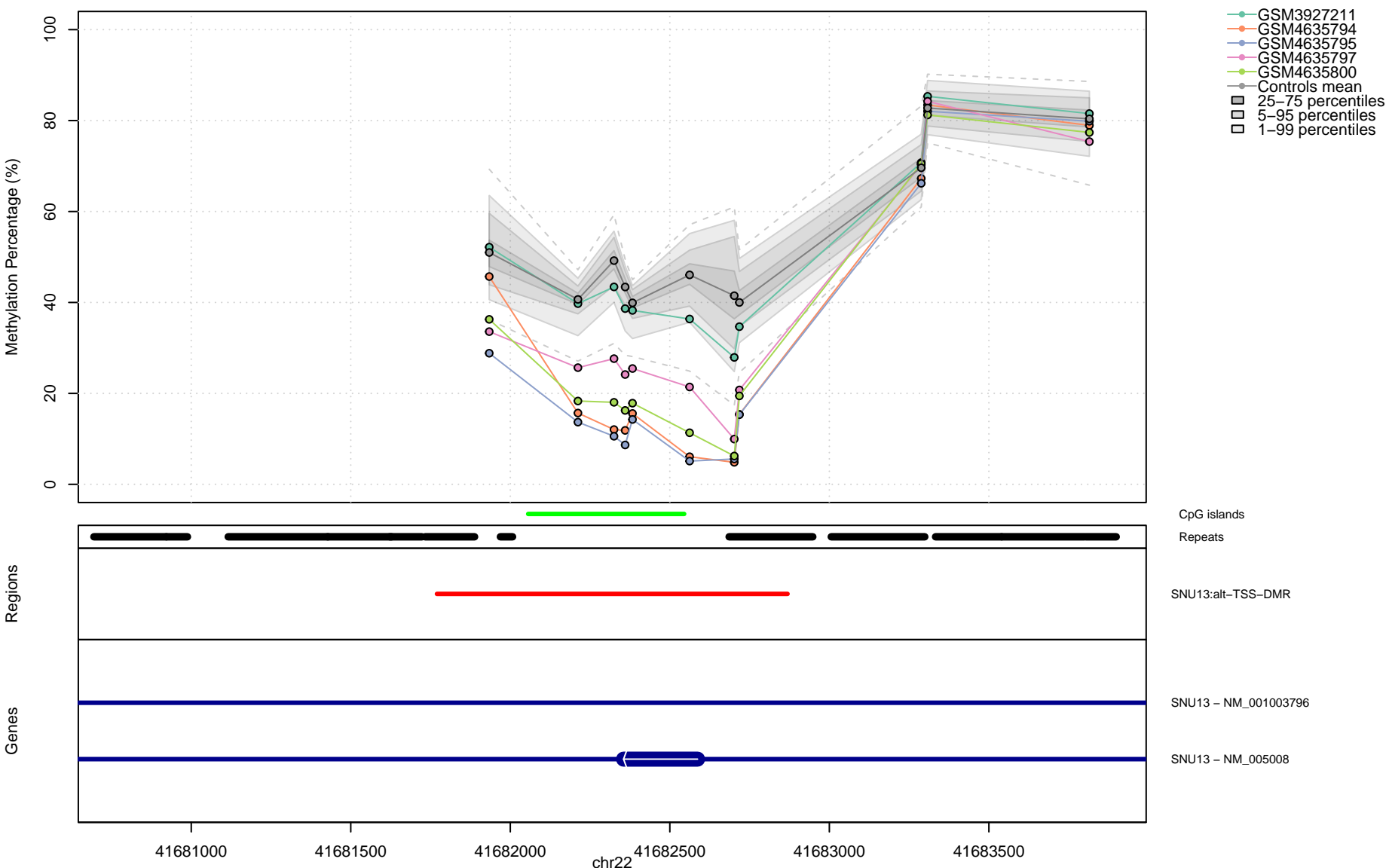

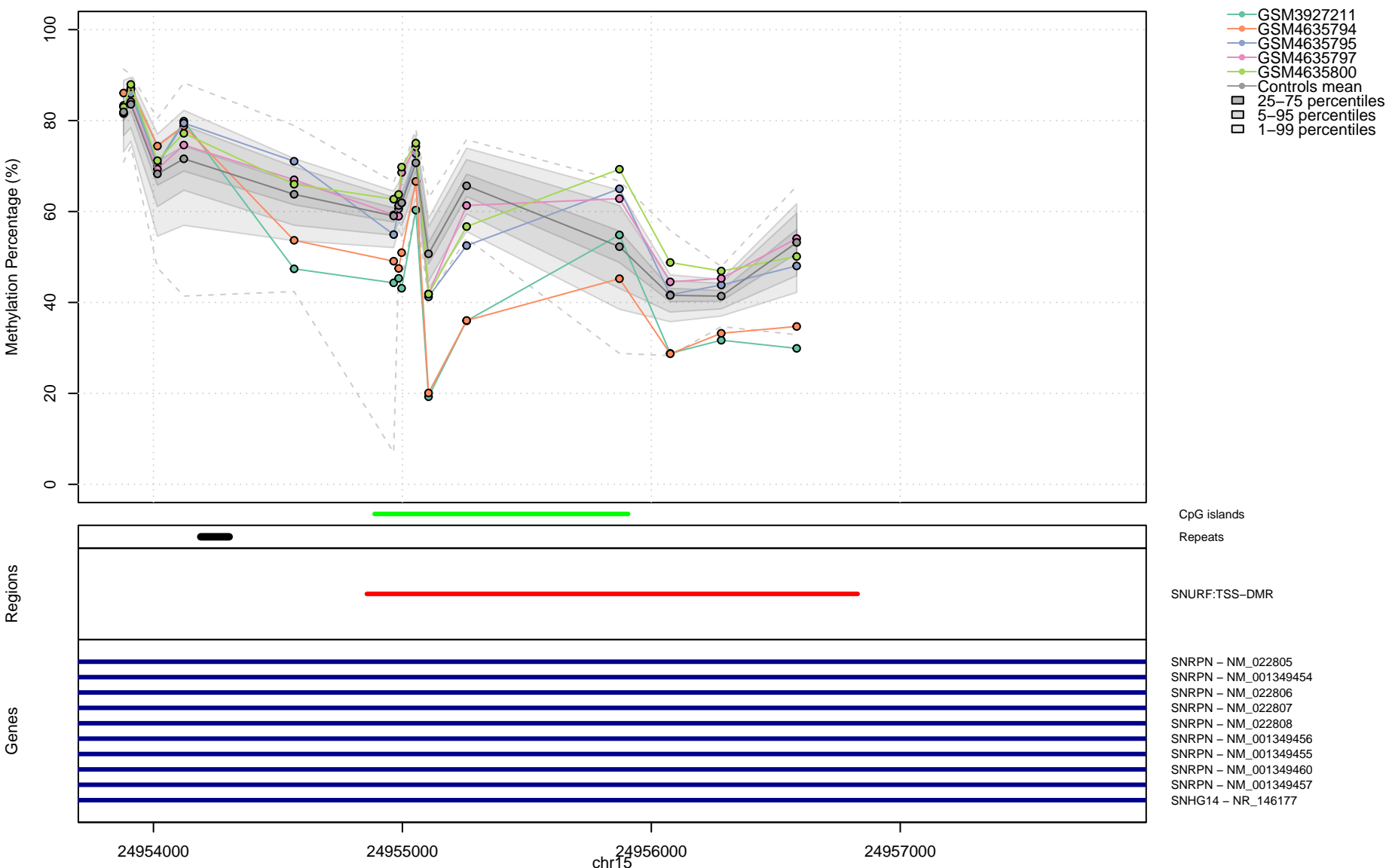

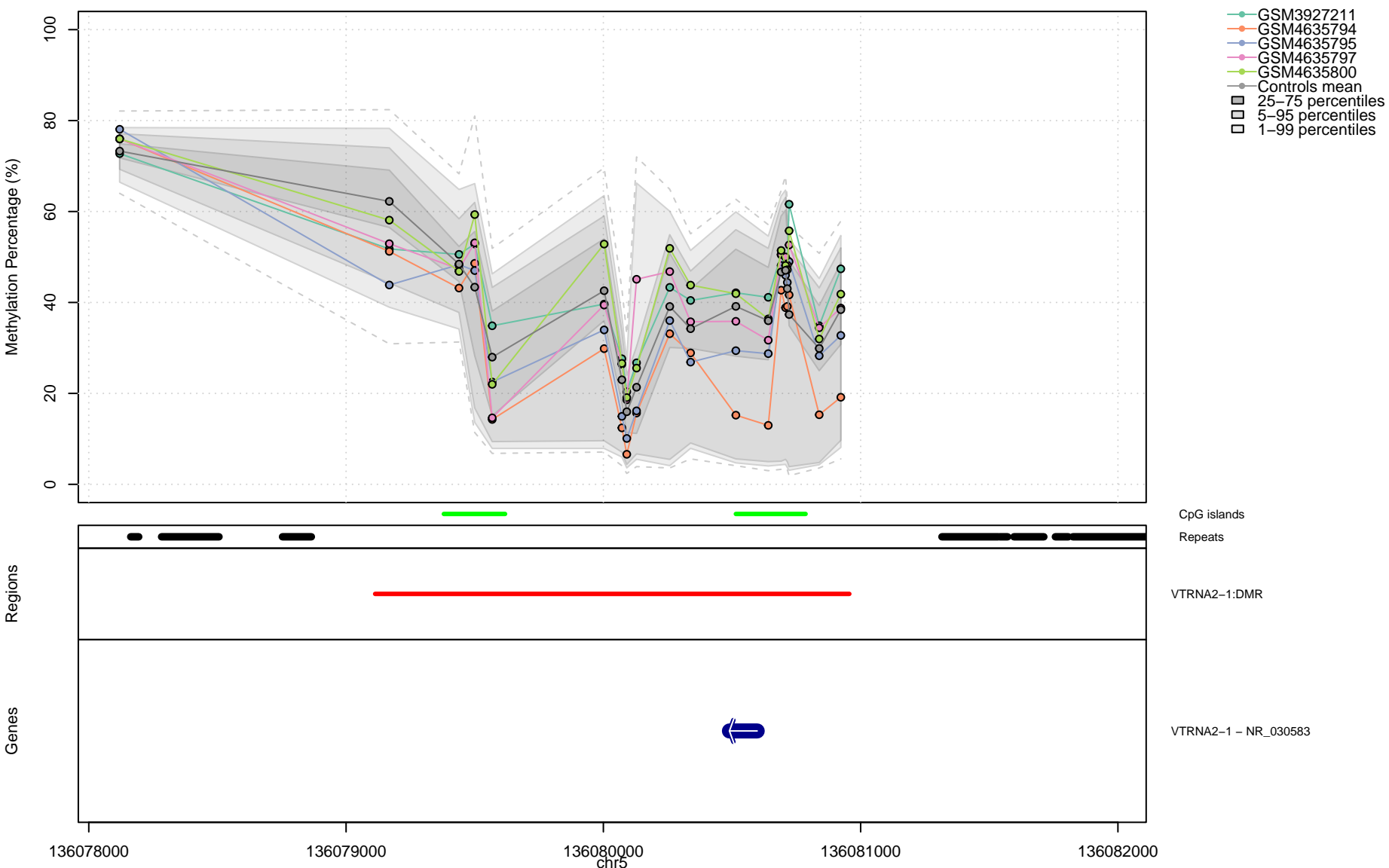

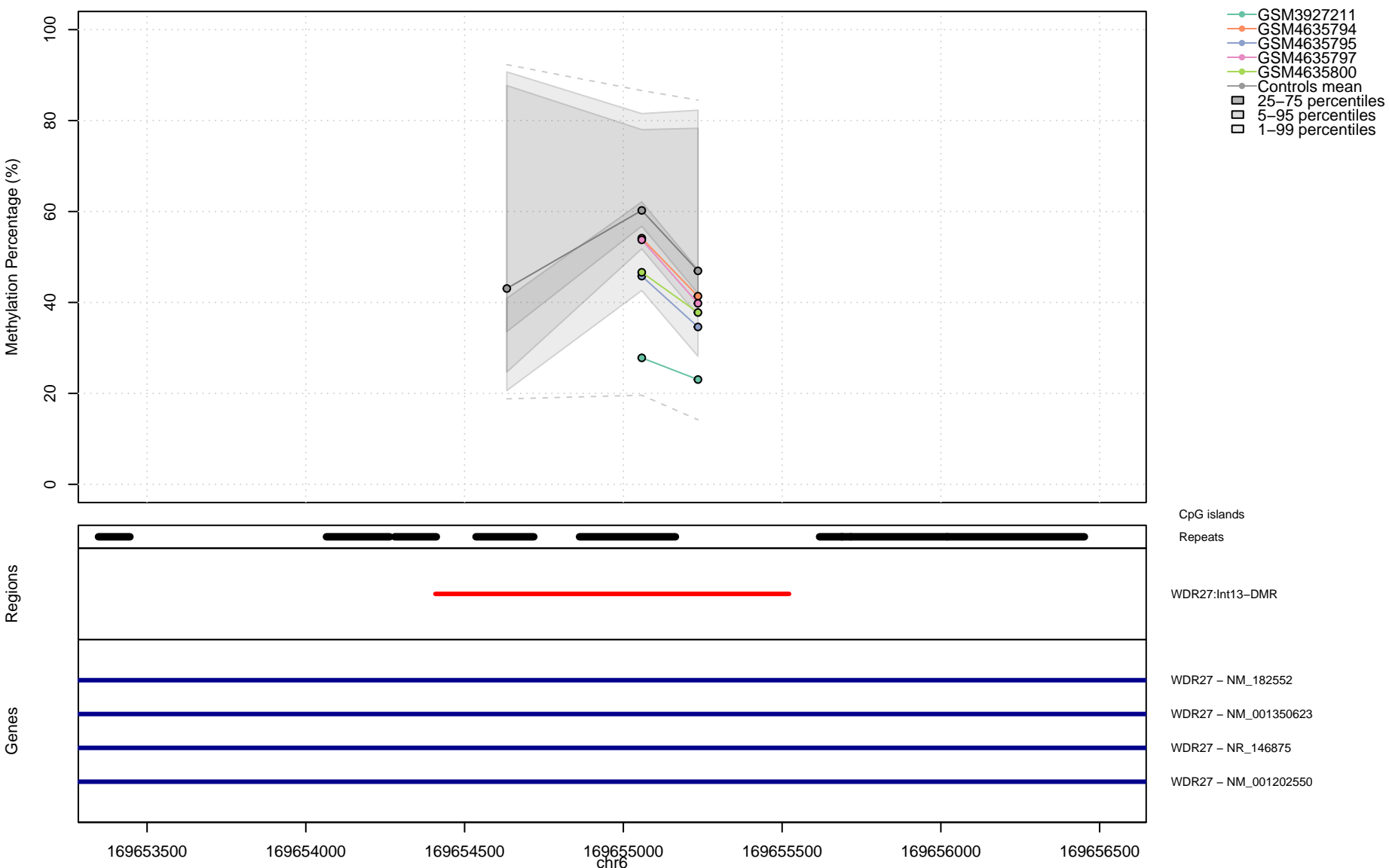

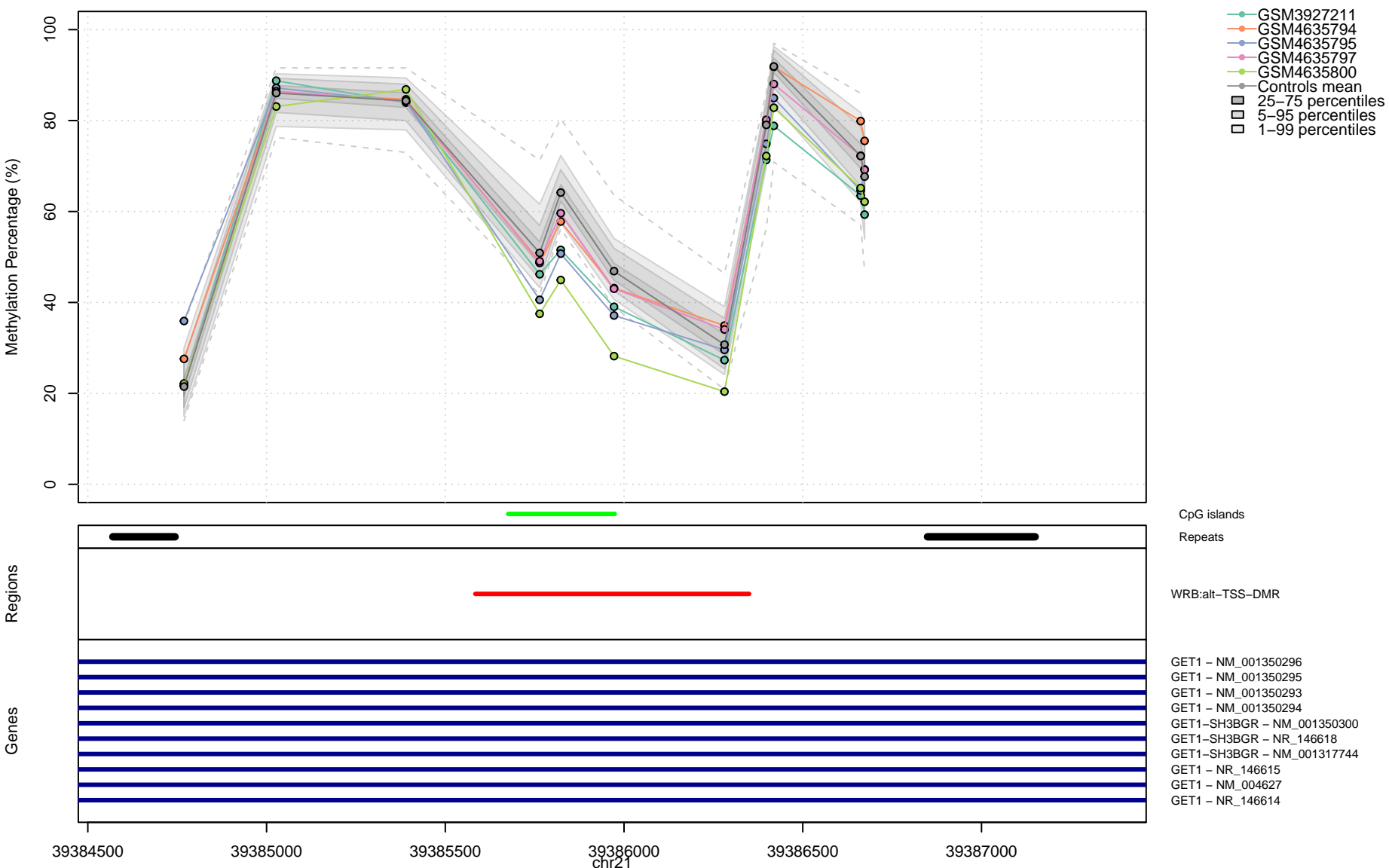

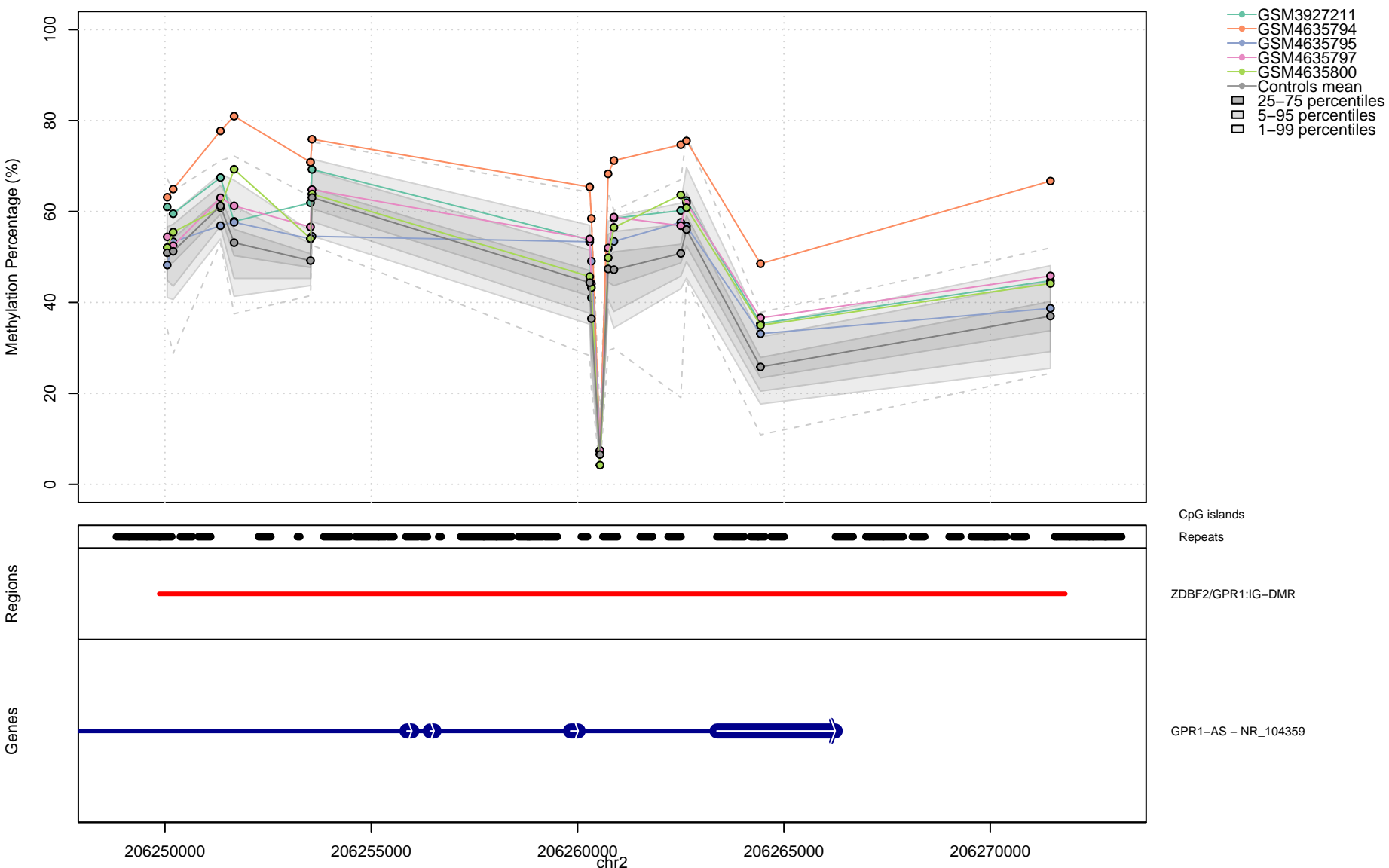

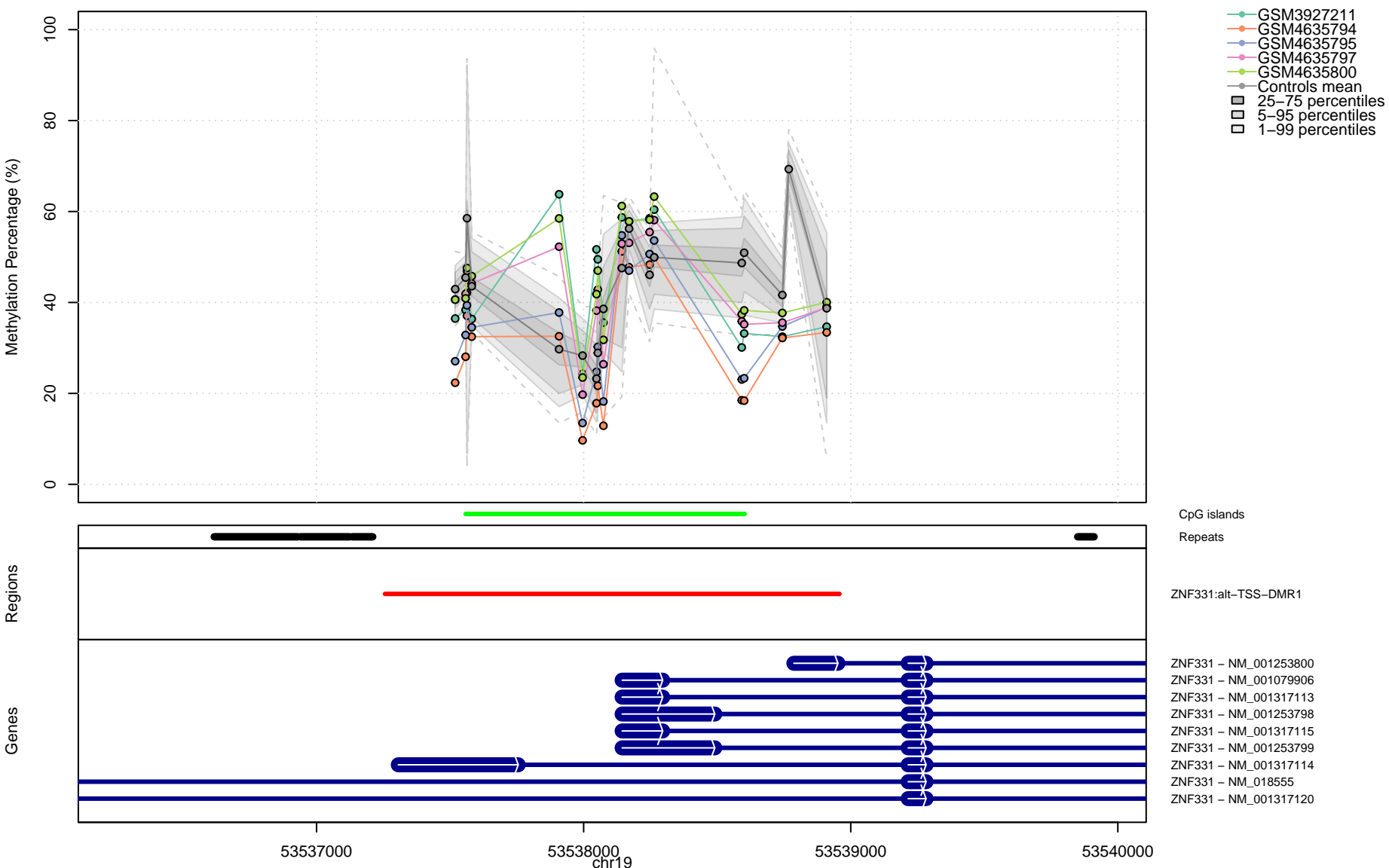

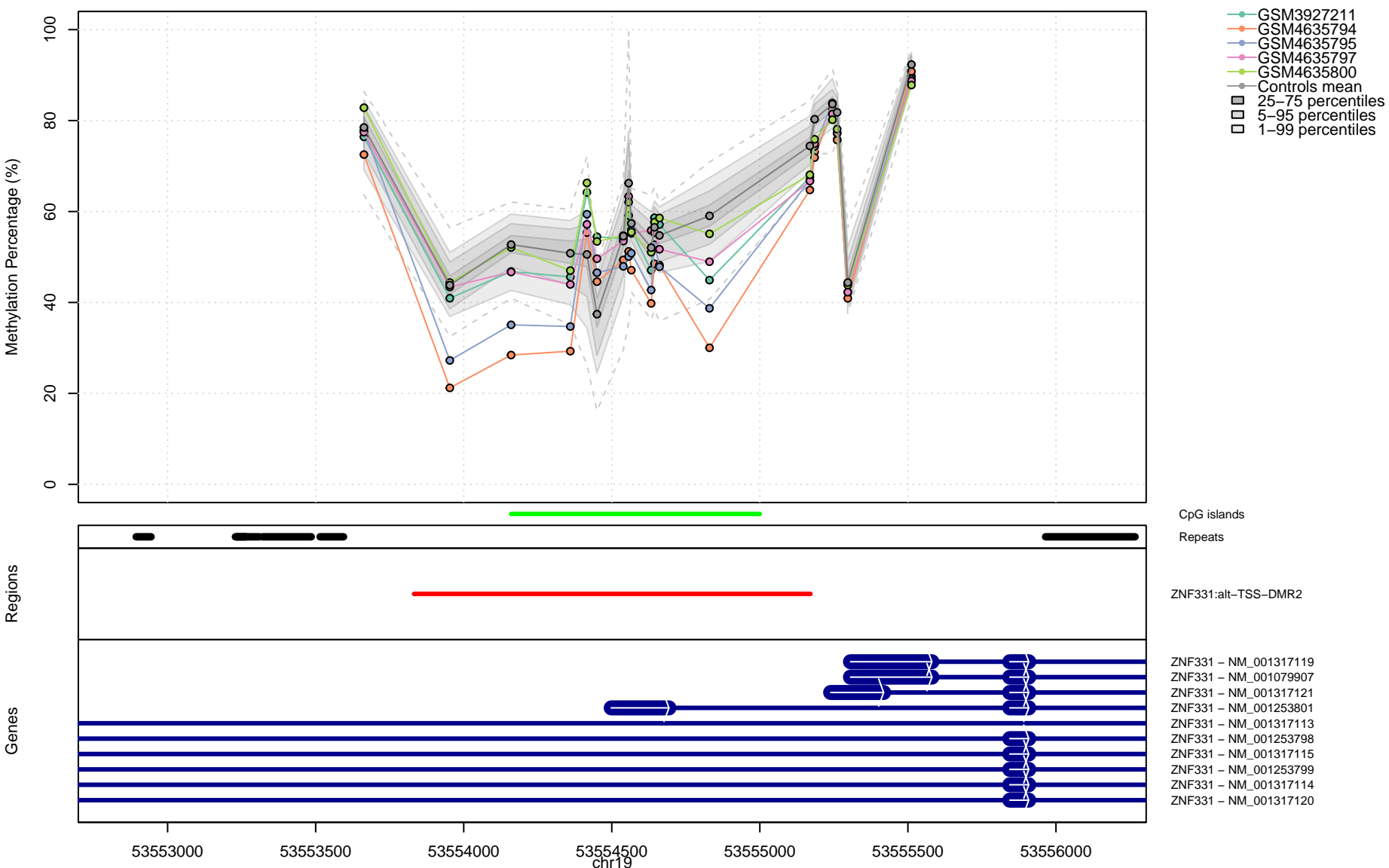

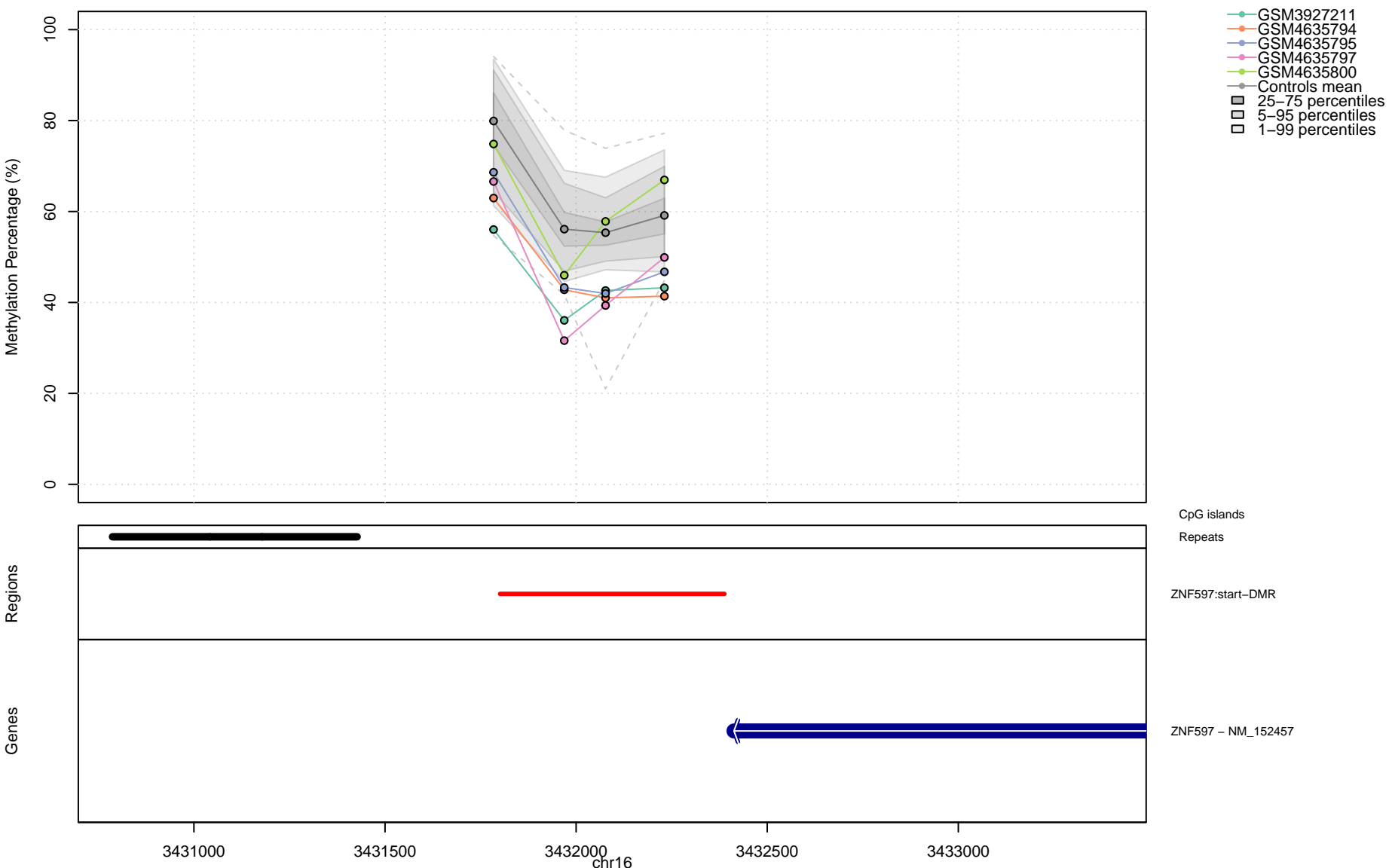

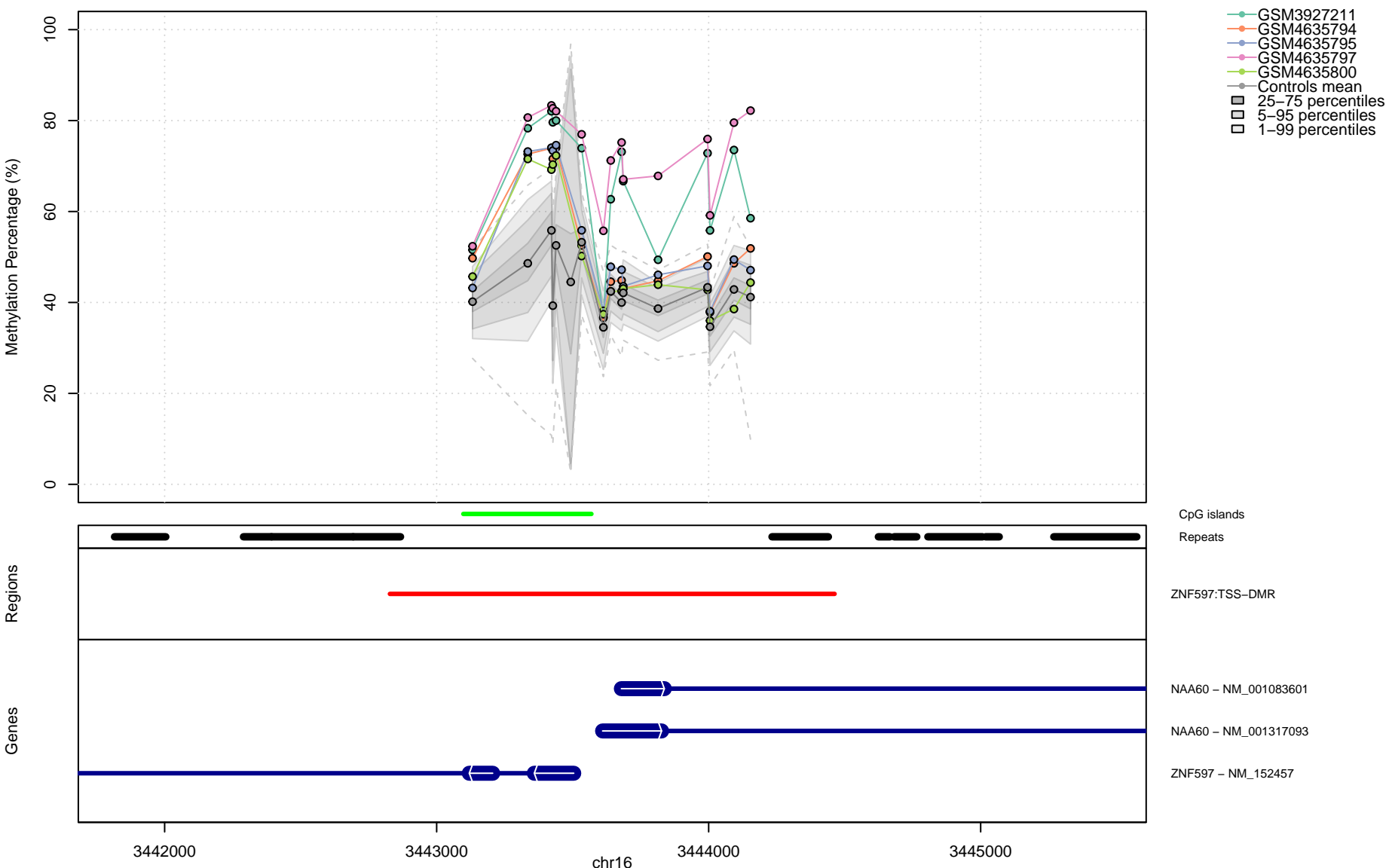

Supplement: Supplementary file 2 — Additional file 2. DNAm profile of imprinted regions in BWS patients. [file 13148_2022_1403_MOESM2_ESM.pdf]
