# Supplementary material for: Identification of differentially methylated regions in rare diseases from a single-patient perspective
Source: Clin Epigenetics. 2022 Dec 16;14:174. doi: 10.1186/s13148-022-01403-7 (PMC9758859; doi:10.1186/s13148-022-01403-7)
Supplement: Supplementary file 5 — Additional file 5. Supplementary Figures and Tables. Boxplots for mean Pearson correlation, standard deviation and entropy; table of semi-simulated DMRs; AUC and precision/recall curves; normalization of BWS patients; DMRs identified in BWS patients; influence of age on DMR identification. [file 13148_2022_1403_MOESM5_ESM.pdf]

Identification of differentially methylated regions in rare diseases from a single patient perspective

Robin Grolaux, Alexis Hardy, Catharina Olsen, Sonia Van Dooren, Guillaume Smits, Matthieu Defrance

Supplementary Figures and Tables

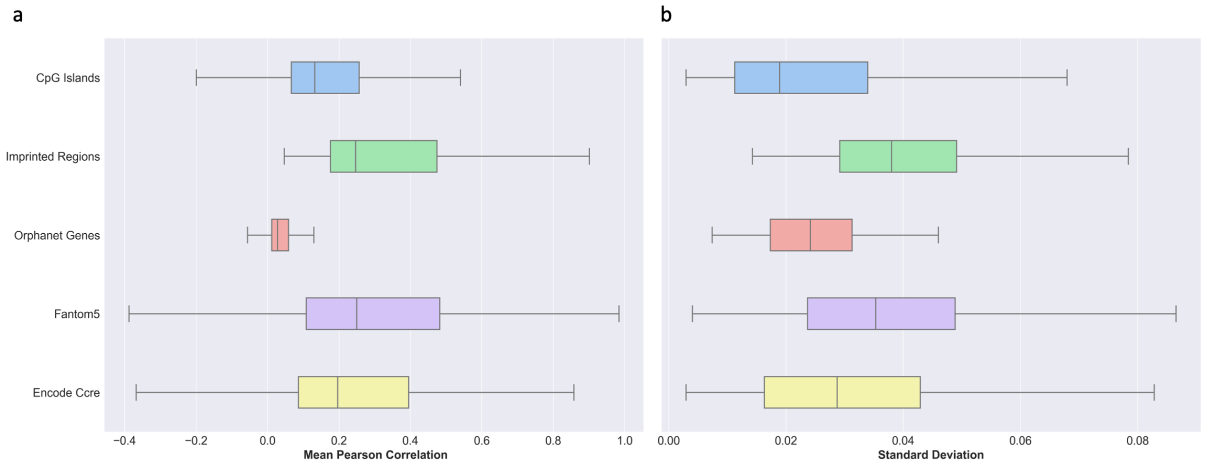

Figure S1. a) Distribution of mean pairwise Pearson correlation of CpGs in different regions of biological interest. b) Distribution of the standard deviation of the Beta values of CpGs in the same regions. Values were computed using a cohort of 521 unaffected patients.

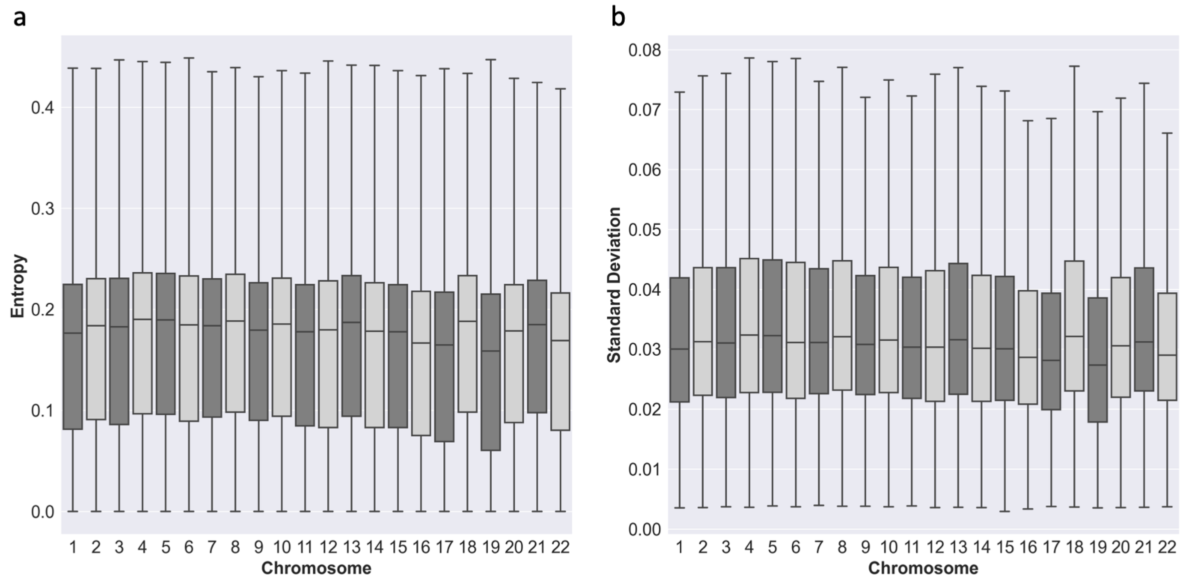

Figure S2. a) Distribution of Shannon's entropy level of CpGs at the autosome scale. b) Distribution of the standard deviation of the Beta values of at the autosome level. Values were computed using a cohort of 521 unaffected patients.

Window category	1 CpG	2 CpGs	3 CpGs	4 CpGs	5 CpGs	6 CpGs	7 CpGs
Total number of windows	423090	213287	118506	74790	50549	34573	24308
Initial number of windows selected	31496	15988	11412	7141	4813	3250	2265
Number of post-filters windows to be modified	30260	14869	10483	6461	4321	2899	2000

Table S1. Number of windows per category of fixed CpG count.

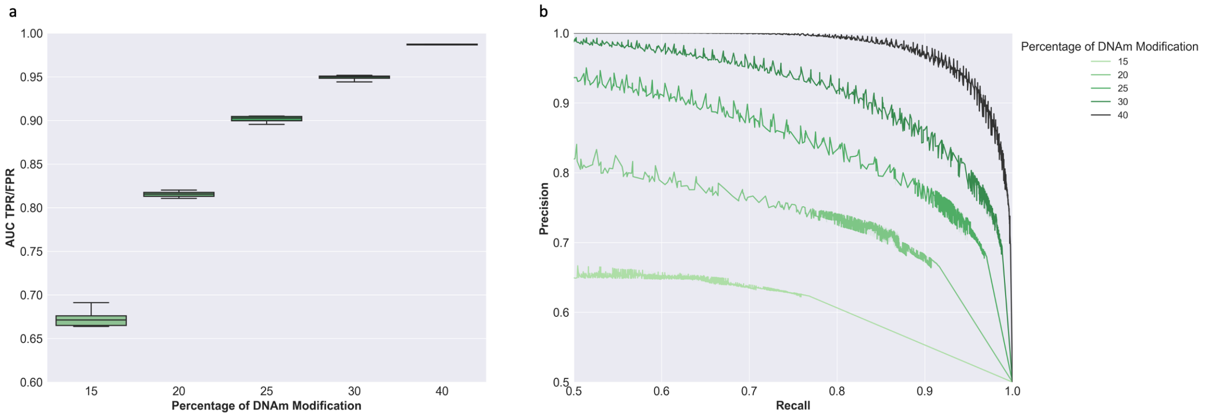

Figure S3. Area under the curve of the True positive and False positive rates (a) and precision recall curves (b) in low noise data (5% noise) at different level of added methylation (True signal).

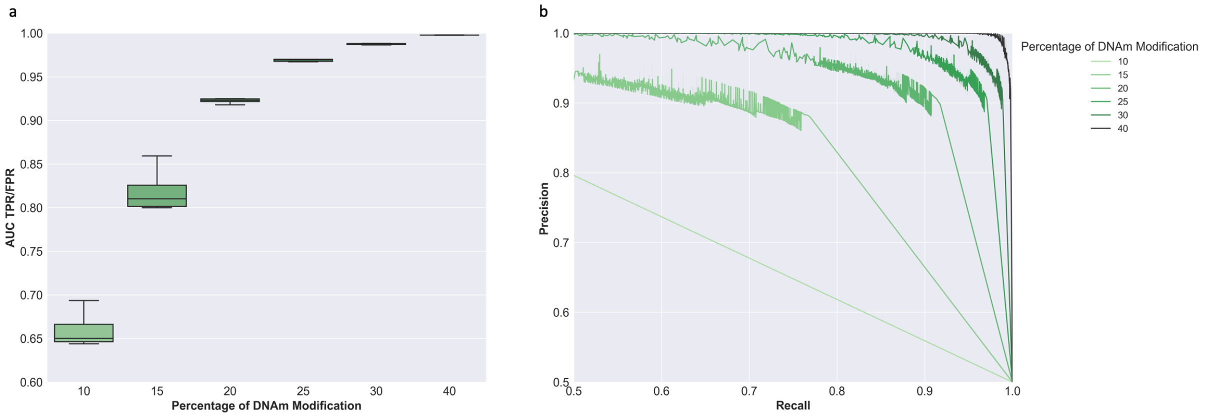

Figure S4. Area under the curve of the True positive and False positive rates (a) and precision recall curves (b) in high noise data (10% noise) at different level of added methylation (True signal).

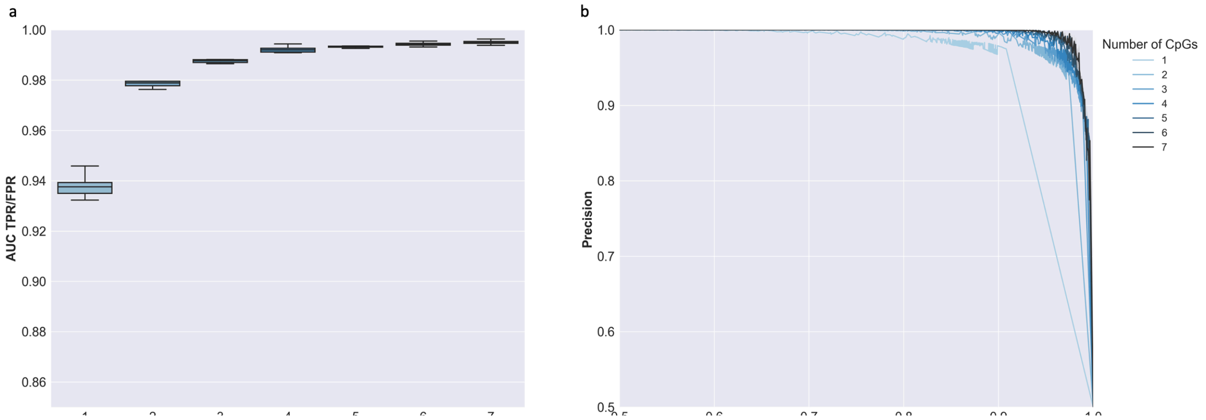

Figure S5. Area under the curve of the True positive and False positive rates (a) and precision recall curves (b) in low noise data (5% noise) for different number of CpGs per window (30% of True signal).

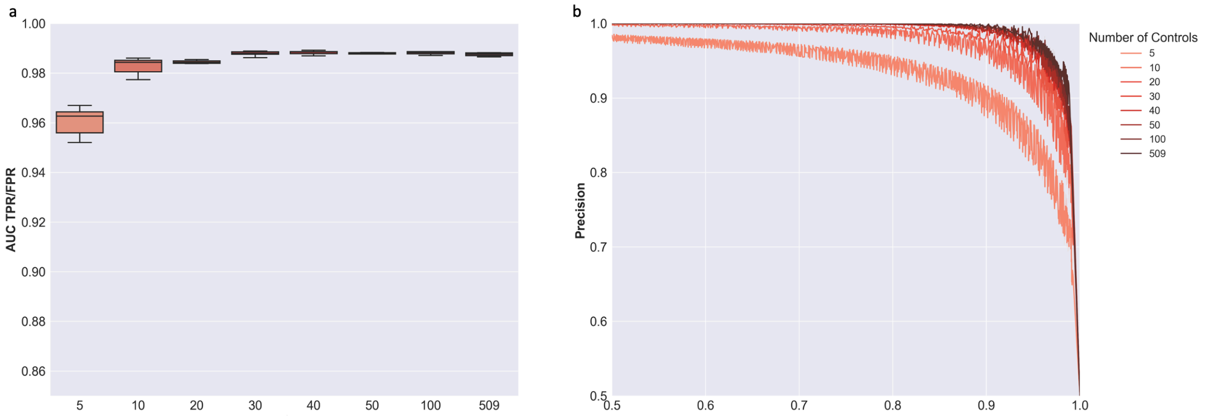

Figure S6. Area under the curve of the True positive and False positive rates (a) and precision recall curves (b) in low noise data (5% of noise) for different number of samples in the control population (30% of True signal).

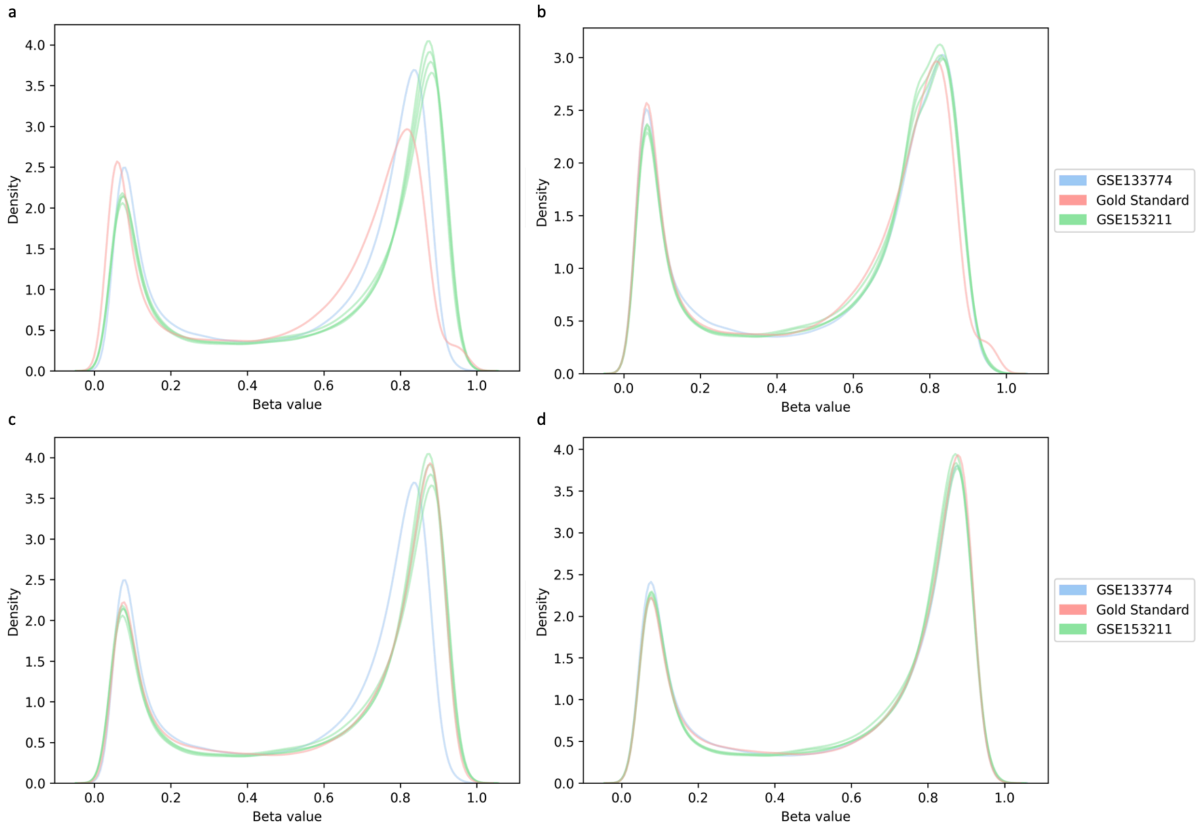

Figure S7. a) Distribution of beta values before normalization of samples from GSE133774 and GSE153211 with the gold standard constituted by controls of the same study. b) Distribution of beta values after normalization of samples from GSE133774 and GSE153211 with the gold standard constituted by the mean of controls of the same study. c) Distribution of beta values before normalization of samples from GSE133774 and GSE153211 with the gold standard constituted by the mean of GSE152026. d) Distribution of beta values after normalization of samples from GSE133774 and GSE153211 with the gold standard constituted by the mean of GSE152026.

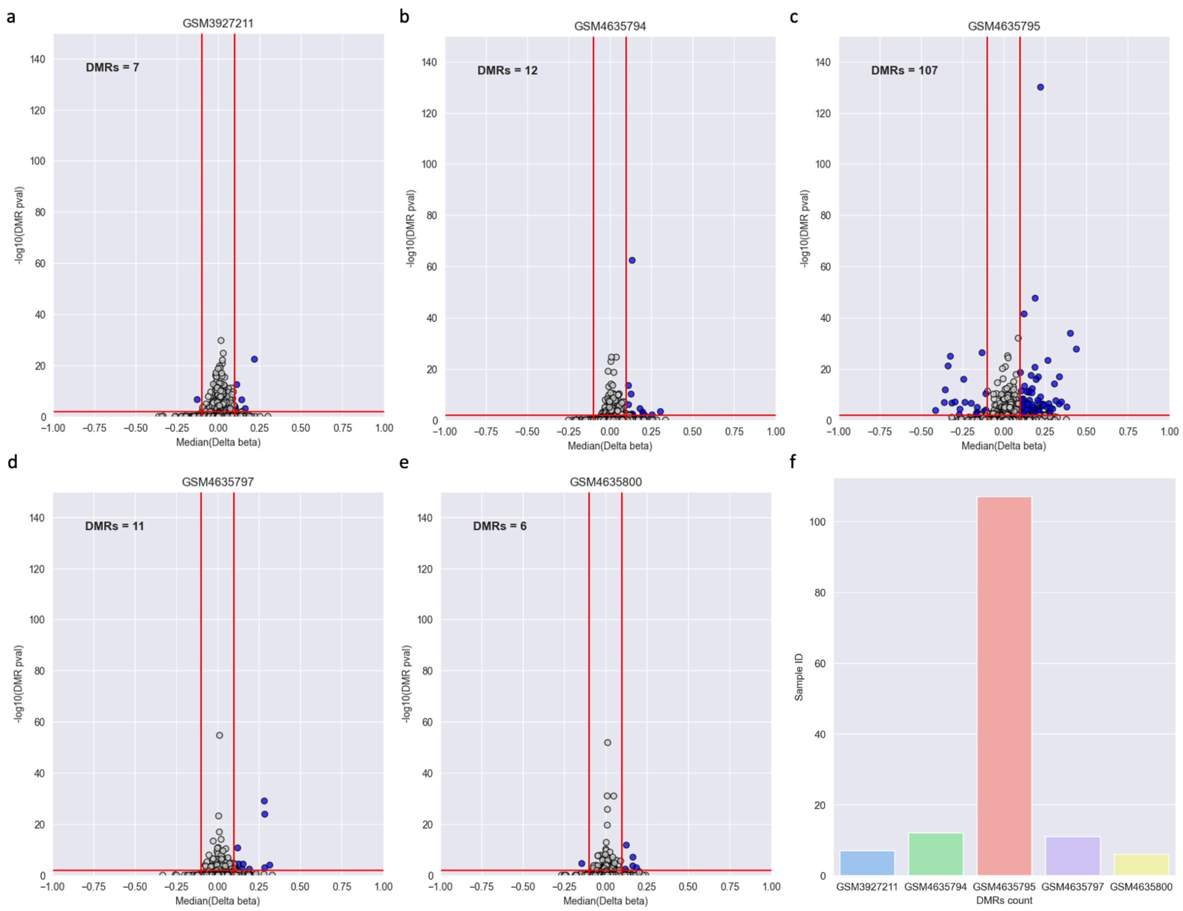

Figure S8. a-e) DMRs identification in the five patients suffering from BWS and MLIDs. Points represent windows tested for differential methylation. Red lines represent the threshold on the -log10(aggregated p-value) at 2 and on the median methylation difference between CpGs of the patient and the controls for a given region at -15 and 15. Grey point do not pass the thresholds, blue points do. The number of positives regions is indicated by "DMRs=") Count of the number of DMRs identified per patients.

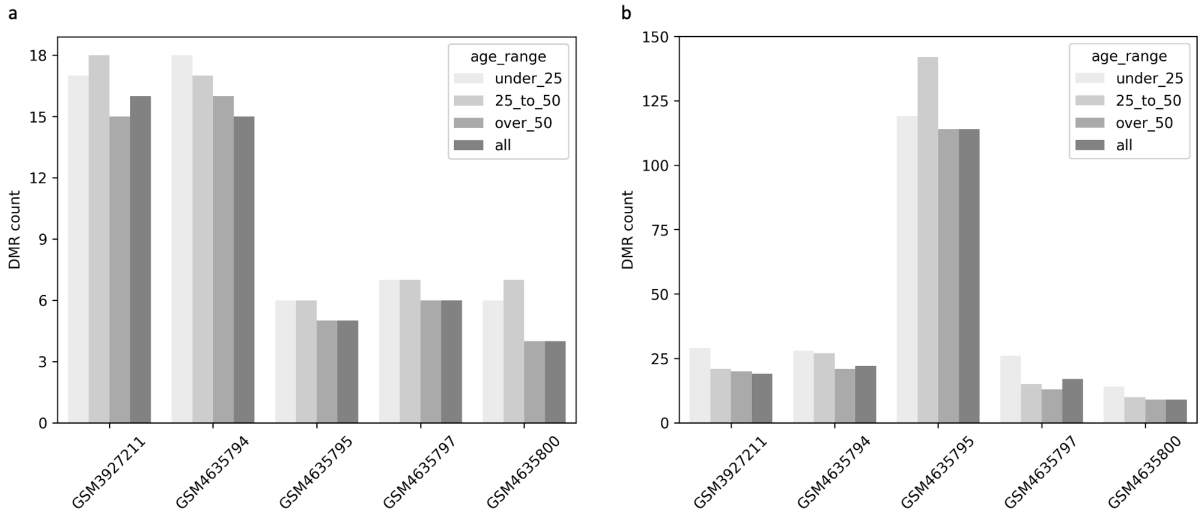

Figure S9. a Number of DMRs identified in known imprinted regions in the five patients suffering from BWS and MLIDs using different subsets of the control population to represent different age categories. b Number of DMRs identified in the whole array-based epigenome in the five patients suffering from BWS and MLIDs using different subsets of the control population to represent different age categories. Mean age under_25: 21.4 yo ; mean age 25_to_50: 37.8yo ; mean age over_50: 57.8 yo; mean age all: 38.68 yo
